# Supplementary material for: Bipolar androgen therapy plus nivolumab for patients with metastatic castration-resistant prostate cancer: the COMBAT phase II trial
Source: Nat Commun. 2024 Jan 2;15:14. doi: 10.1038/s41467-023-44514-2 (PMC10762051; doi:10.1038/s41467-023-44514-2)
Supplement: Supplementary file 1 — Supplementary Information [file 41467_2023_44514_MOESM1_ESM.pdf]

A.

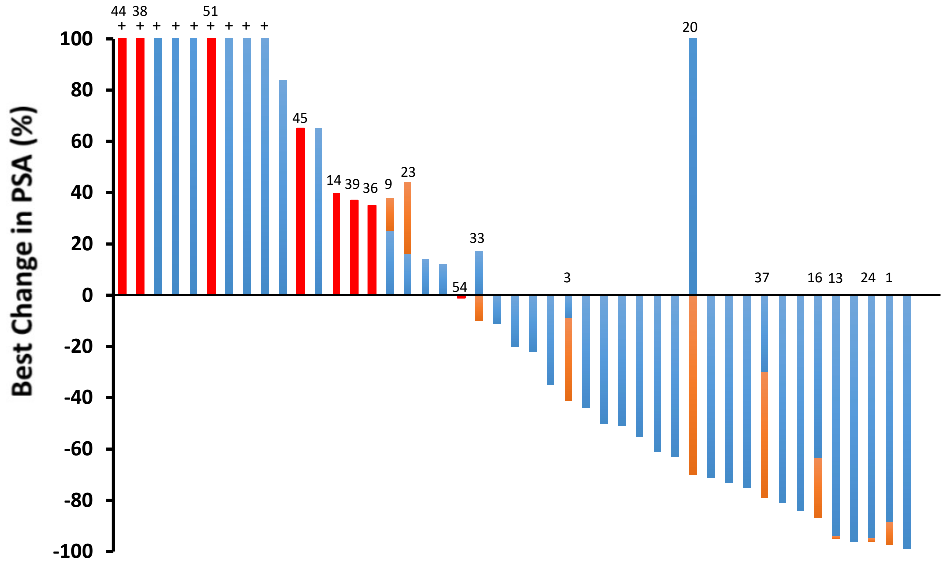

C.

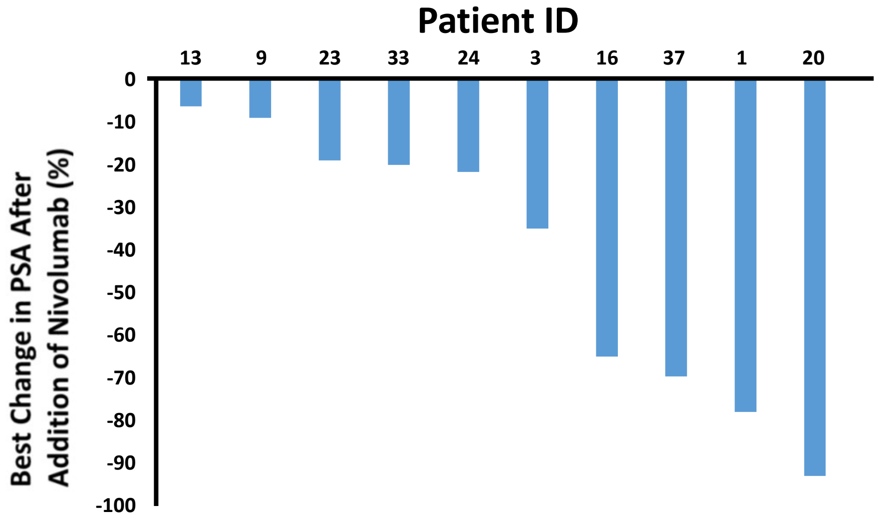

B.

Patient ID	Sequencing	Genomic/Molecular Alteration
14	NGS	EGFR amplification, PTEN (splice site 635-1G>A), CDKN1B (p.S125fs*1)
36	NGS	MYC amplification
38	N/A	N/A
39	WGS	PTEN loss, ZNF292 Loss, RNF (p.P231L), 2.19 muts/MB
44	WGS	AR (p.H875_L881Del), BRCA2 (p. E2183Vfs*5), CHD7 (p.Q2426Sfs*42), PI3K3CB (p. Y962N), TMPRSS2-ERG, 3.36 mut/MB
45	NGS	AR amplification, PTEN loss
51	NGS	AR (p.T8785S), SETD2 (p.N2526Kfs*8), TMPRSS2-ERG
54	NGS	AR amplification, ESR1 amplification, EGFR amplication, TP53 (p.S127C), TP53 (p. R273C)

D.

Patient ID	Sequencing	Genomic/Molecular Alteration
20	WGS	MLH1 (p.T206Hfs*23), KMT2D (p.P2277H), SPOP (p.Y83C), BRCA2 (p.I605Nfs*11), SMARCA1 (p.K834Rfs*50), PIK3R1 (p.N602Tfs*60), RNF43 (p.G659Vfs*41), TMPRSS2-ERG, TMB 13.97 muts/Mb
37	NGS	BRCA2 loss, TP53 (p.R175H), TMPRSS2-ERG
3	N/A	N/A
23	NGS	RB1 loss, PI3KA (p.N345Y)
33	NGS	ATM (H1264*fs), BRCA2 (A938fs*21)
16	NGS	AR amplification, PTEN loss
9	WGS	AR amplification, PTEN loss, TMPRSS2-ERG fusion, TMB: 1.37 muts/MB
1	WGS	AR amplification, BRAF amplification, EZH2 amplification, TMPRSS2-ERG, TMB 0.83 muts/Mb
13	WGS	ZFHX3 loss, CHD6 amplification, IDH1 amplification, TMPRSS2-ERG, TMB 1.85 muts/Mb
24	WGS	AR amplification, ATM (p.Q2724H), APC (p.D1425Wfs*10), ARID1A (p.E1780Kfs*2), TMB 1.71 muts/Mb

Supplementary Figure 1. Differential Effect of BAT and Nivolumab with Associated Molecular and Genomic Alterations.
A. Waterfall plot of best PSA response in N=45 patients . Blue bar represents the PSA response on BAT (C4D1). Any patient with new or further decline in PSA with the addition of nivolumab is noted in orange. Patients that received only BAT (no addition of nivolumab) are shown in red. De-identified patient number listed for specific patients of interest. + denotes >100% increase as best PSA response. B. Molecular and genomic alternations in patients treated with BAT alone. NGS = clinical grade next generation sequencing, WGS = whole genome sequencing, N/A = no data available. C. Ten patients had a new or continued decline in PSA with the addition of nivolumab. Waterfall plot of best PSA change in these 10 patients using cycle 4 day 1 (prior to addition of nivolumab) as baseline. De-identified patient number is provided. D. Molecular and genomic alternations in patients with a declining PSA on nivolumab. NGS = clinical grade next generation sequencing, WGS = whole genome sequencing, N/A = no data available. Source data are provided as a Source Data file.

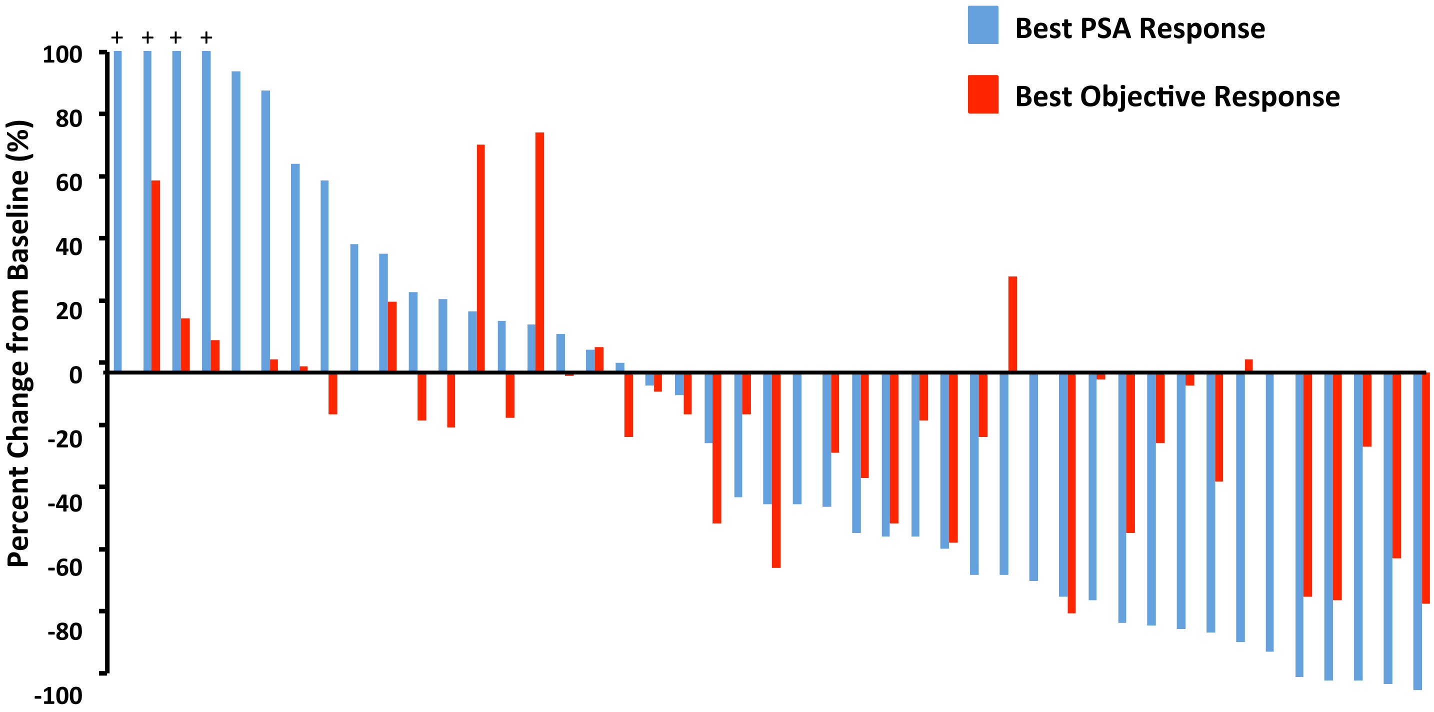

Supplementary Figure 2. Best Change in PSA Level and Target Lesions Stratified by Patient Following Treatment with BAT and Nivolumab. Waterfall plot showing best change in PSA (blue) and sum of target lesions (red) paired for each individual patient (N=45). + denotes >100% increase as best PSA response. Source data are provided as a Source Data file.

A.

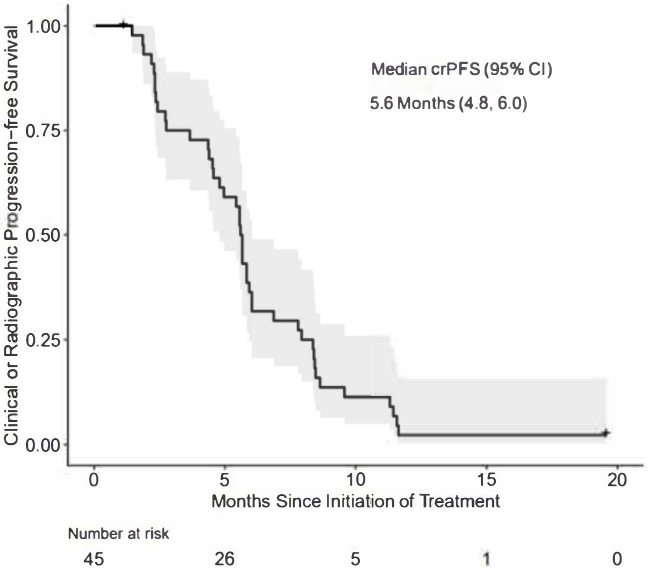

B.

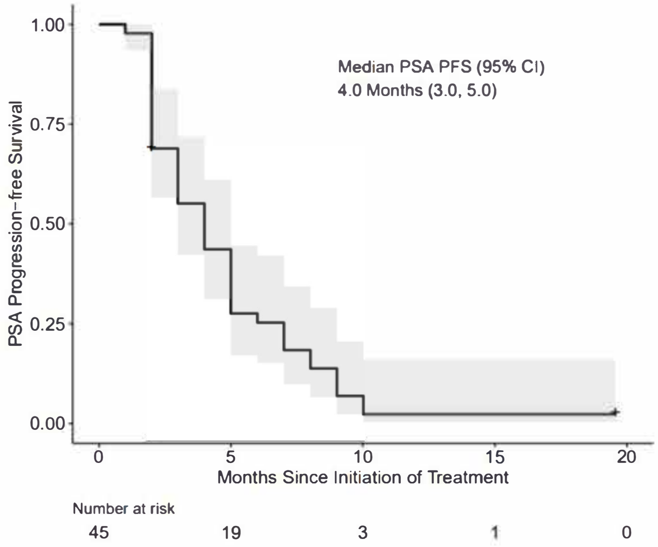

Supplementary Figure 3. Clinical or Radiographic Progression-Free Survival , PSA Progression-Free Survival, and Median Duration of Treatment Estimates in Patients with mCRPC Treated with BAT in Combination with Nivolumab. Kaplan-Meier curves of (A) clinical or radiographic progression-free survival (rPFS), (B) PSA progression-free survival (PSA PFS) and (C) duration of treatment estimates are shown. The median crPFS was estimated at 5.6 months (95% Confidence interval: 4.8-6.0 months). The median PSA PFS was estimated at 4.0 months. (95% Confidence interval: 3.0 – 5.0 months). The median duration of treatment was estimated at 5.6 months (95% Confidence interval: 5.6-8.4 months). The shaded area represents 95% confidence region.

C.

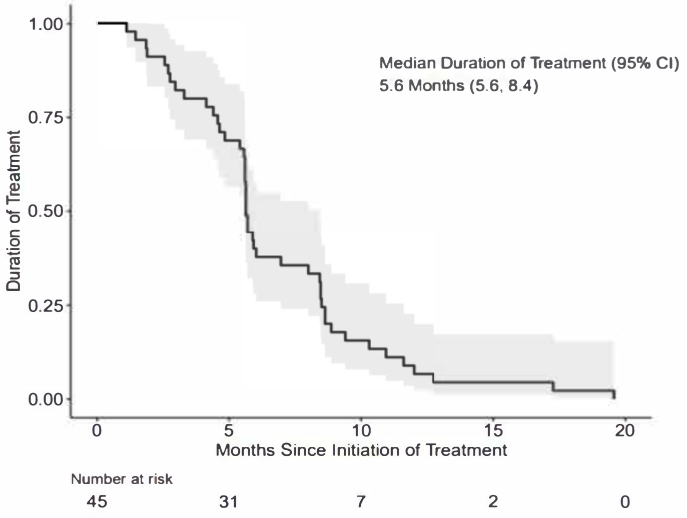

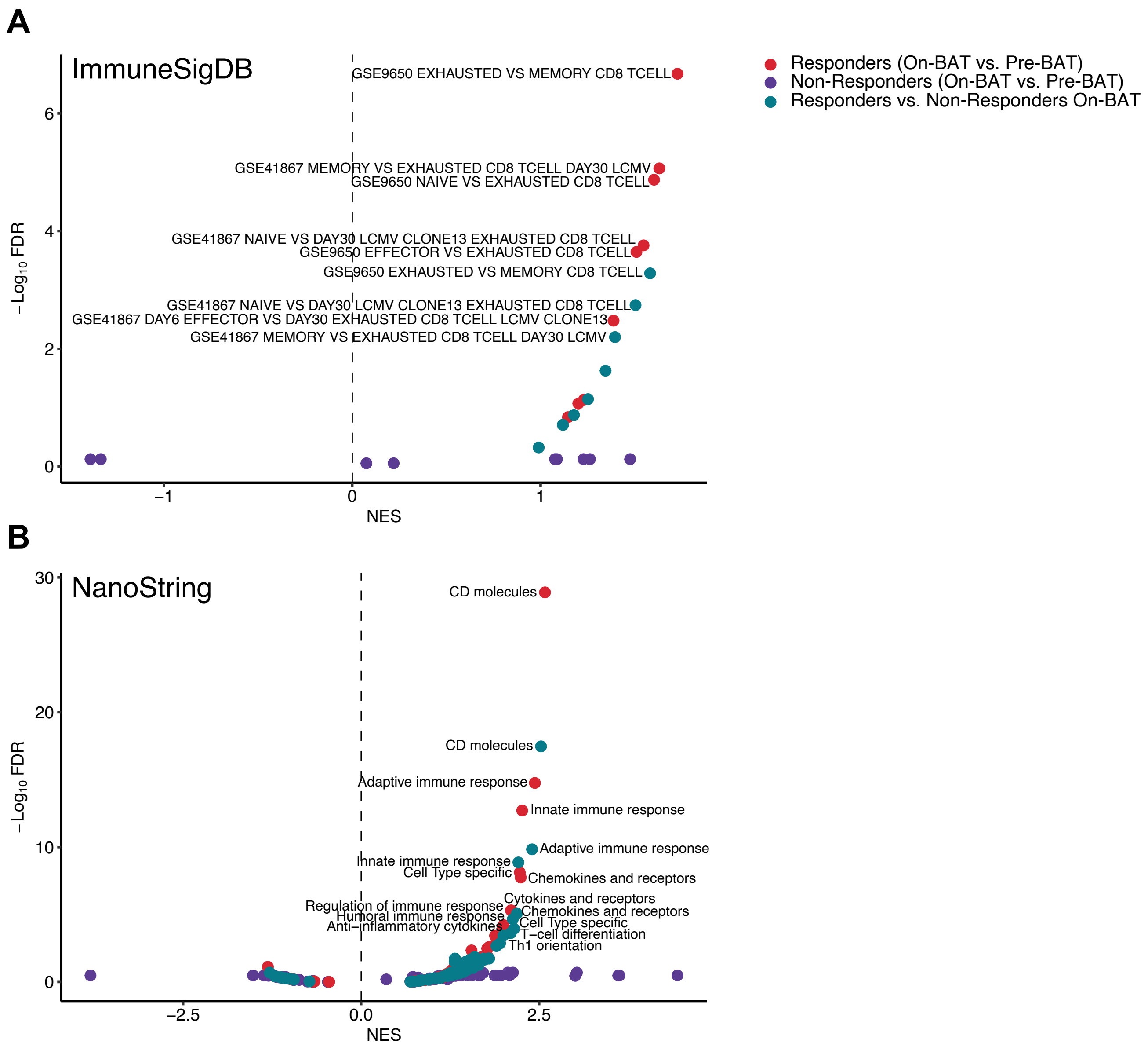

Supplementary Figure 4. BAT Induced Changes in Gene Set Enrichment Analysis.
Gene set enrichment analyses of (A) ImmuneSigDB associated with exhaustion and (B) Nanostring PanCancer Immune profiling gene sets comparing baseline (pre-BAT) to C4D1 (On-BAT) in responders (red dots) and non-responders (purple dots) or responders to non-responders on C4D1 (On-BAT) (green dots). N=12 patients analyzed. FDR adj p, p value adjusted for false discovery rate. NES, normalized enrichment score.

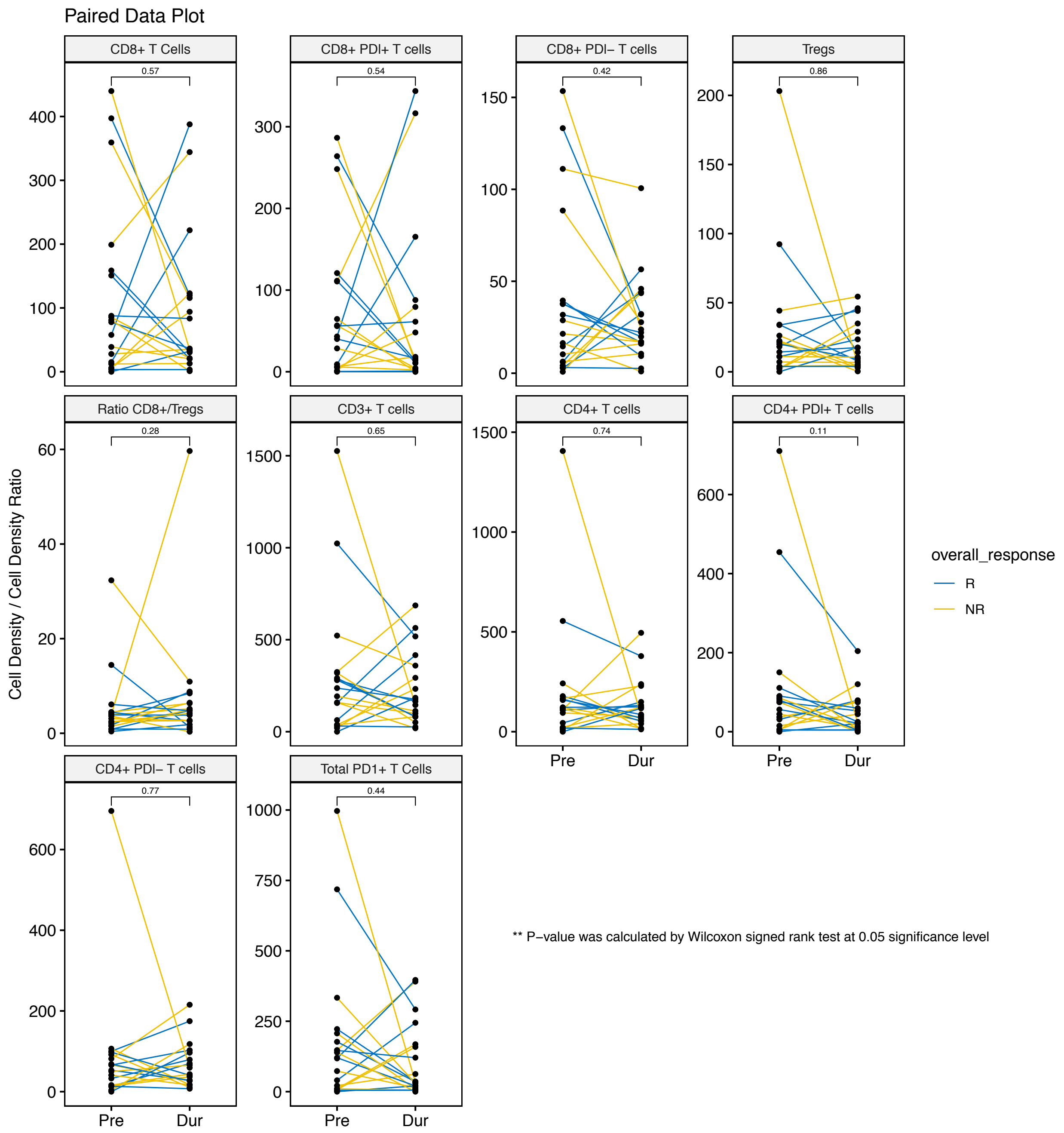

Supplementary Figure 5. Effect of BAT on Change in T cell Densities in Individual Patients Stratified by Response to Treatment. The densities of different T cell subtypes are shown for individual patients. “Pre” denotes the density of T cells prior to the initiation of BAT. “Dur” is the density of T cells at C4D1 (i.e. on BAT). For individual patients, “pre” and “dur” values are connected via a yellow (non-responder) or blue (responder) line. No significant change in cell subtype was induced by BAT irrespective of treatment response. N=19 patients had paired samples and were included in this analysis. Source data are provided as a Source Data file.

Number	Cell types	Phenotype
1	T Cells All CD3+	CD3+
2	CD8+ T Cells All	CD3+CD8+
3	CD4+ CD4+ Cells All	CD3+CD4+
4	Regulatory T Cells	CD3+CD4+Foxp3+
5	CD8+ T Cells - PD1 Positive	CD3+CD8+PD1+
6	CD8+ T Cells - PD1 Negative	CD3+CD8+PD1-
7	CD4+ Cells - PD1 Positive	CD3+CD4+PD1+
8	CD4+ Cells - PD1 Negative	CD3+CD4+PD1-

Supplementary Table 1: Cell Phenotypes Evaluated in T Cell Panel

Odds ratio, their 95% Condifidence Interval, and p-value

	Odds Ratio (95% CI)	P Value
**Pathway**		
AR Pathway	0.25 (0.05, 1.01)	0.060
PI3K Pathway	2.67 (0.63, 12.75)	0.194
Tumor Suppressors	3.06 (0.76, 13.43)	0.123
WNT Pathway	NA (0.00-NA)	0.995
HRR Pathway	1.81 (0.36, 10.40)	0.478
Chromatin Pathway	3.03 (0.66, 16.99)	0.170

[1] P-values from univariate logistic regression

Supplementary Table 2: Association of Key Molecular Alterations With PSA50 Response Rate Following BAT+ Nivolumab

AR pathway = AR, FOXA; PI3K pathway = PTEN, PIK3CA, AKT1; WNT pathway = APC, CTNNB1; HRR pathway = ATM, BRCA2; Tumor suppressors = TP53, RB1, PTEN; Chromatin regulation = ARID1A, KMT2C, EZH2, KDM6A, KMT2B, KMT2D. P values are derived by univariate logistic regression. Source data are provided as a Source Data file.

	rPFS			OS		
	Median Months (95% CI)	HR (95% CI)	P Value	Median Months (95% CI)	HR (95% CI)	P Value
**Prior Taxane Chemotherapy**						
No	5.8 (4.9, 7.8)	REF	0.55	28.2 (17.3, NA)	REF	0.82
Yes	5.5 (4.7, 8.3)	1.22 (0.64, 2.33)		17.9 (17.2, NA)	1.09 (0.53, 2.25)	
**Number of Prior Lines of AR Therapy**						
1	5.5 (4.9, 6.0)	REF	0.11	24.4 (12.9, NA)	REF	0.6
2 or more	5.8 (4.7, 8.4)	0.58 (0.30, 1.13)		27.6 (17.4, NA)	0.83 (0.4, 1.7)	

Supplementary Table 3: Association of Prior Taxane Therapy and Prior Lines of AR Therapy with rPFS and OS Based on Univariate Cox Regression
P value of two-sided Wald test is shown. No adjustments for multiple comparisons were applied. rPFS = Radiographic Progression-Free Survival, OS = Overall Survival, 95% CI = Confidence Interval, REF = Reference Group.

Patient Characteristics		HR	95% CI	P-value
Age	≥69 vs. <69	0.83	[0.43 - 1.61]	0.590
Race	White vs. Other	1.04	[0.40 - 2.74]	0.937
Gleason Sum	8-10 vs. 6-7	0.77	[0.38 - 1.59]	0.483
Baseline PSA	≥57.6 vs. <57.6	1.19	[0.63 - 2.25]	0.601
Visceral Mets	Yes vs. No	5.26	[1.96 - 14.1]	0.001
Prior Taxane	Yes vs. No	1.22	[0.64 - 2.34]	0.549
Prior Lines of AR Therapy	≥2 vs. 1	0.57	[0.30 - 1.12]	0.107

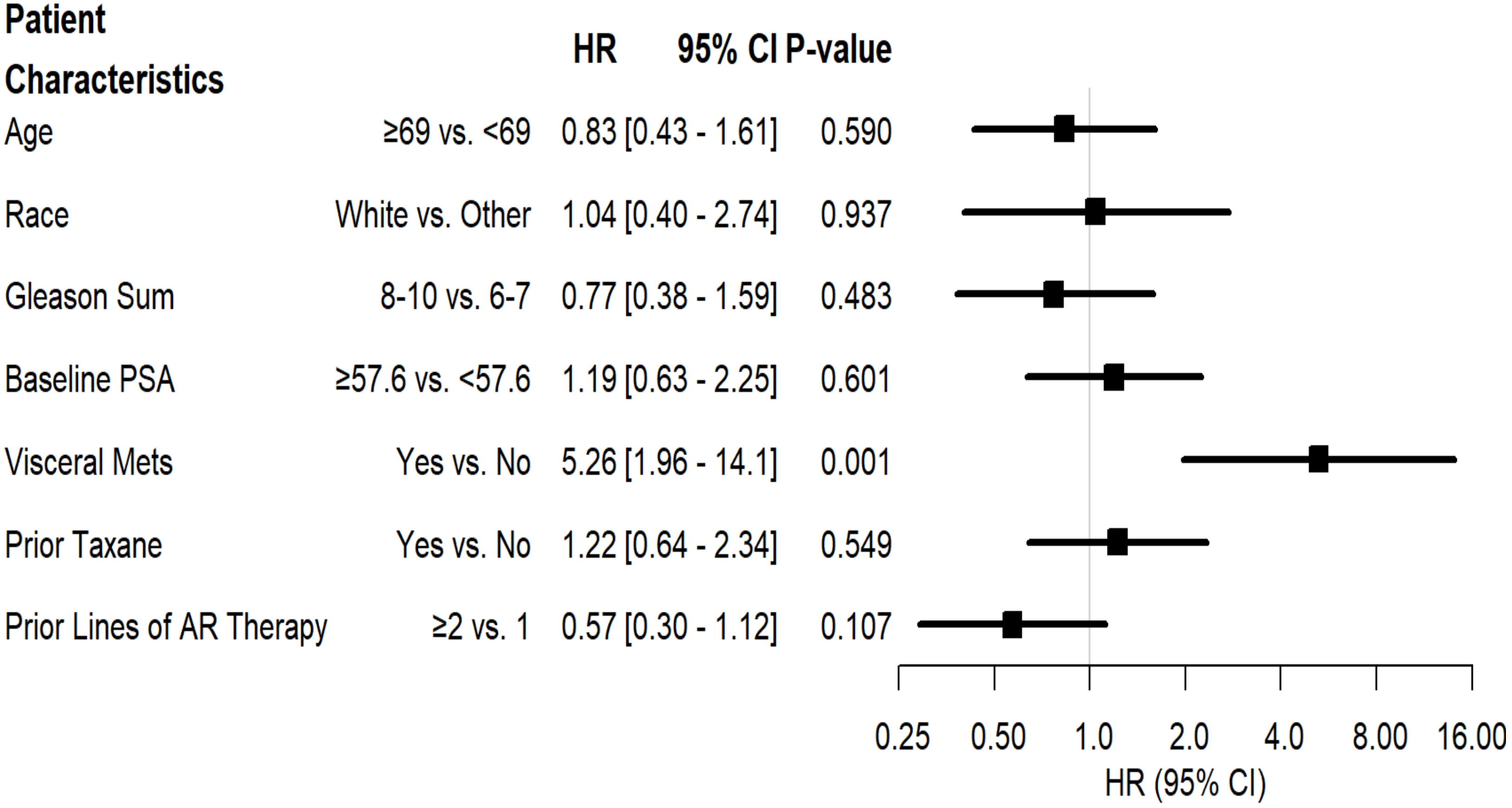

Supplementary Table 4: Baseline Characteristics Forest Plot For Radiographic Progression-Free Survival Based on Univariate Cox Regression Analysis.
P Value of two-sided Wald test is shown. No adjustments for multiple comparisons were applied. HR = Hazard Ratio, 95% CI = 95% Confidence Interval

Patient Characteristics		HR	95% CI	P-value
Age	≥69 vs. <69	1.00	[0.49 - 2.05]	1.000
Race	White vs. Other	1.34	[0.41 - 4.45]	0.628
Gleason Sum	8-10 vs. 6-7	4.63	[1.38 - 15.5]	0.013
Baseline PSA	≥57.6 vs. <57.6	1.37	[0.67 - 2.81]	0.395
Visceral Mets	Yes vs. No	5.62	[2.04 - 15.5]	<0.001
Prior Taxane	Yes vs. No	1.09	[0.53 - 2.25]	0.821
Prior Lines of AR Therapy	≥2 vs. 1	0.82	[0.40 - 1.69]	0.599

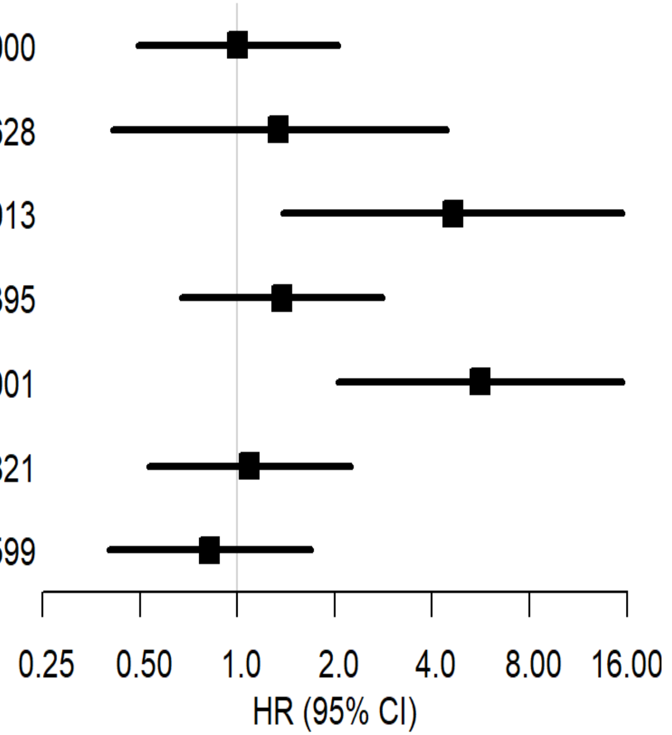

Supplementary Table 5: Baseline Characteristics Forest Plot For Radiographic Overall Survival Based on Univariate Cox Regression Analysis.
P Value of two-sided Wald test is shown. No adjustment for multiple comparisons were applied. HR = Hazard Ratio, 95% CI = 95% Confidence Interval

COMbination of Bipolar Androgen Therapy and Nivolumab in Patients with Metastatic Castration-Resistant Prostate Cancer [COMBAT-CRPC]

PCCTC LOI #: c18-219
BMS Protocol #: CA209-8CE
IND #: IND 138431

Lead Site and Sponsor: Johns Hopkins University

Principal Investigator
Mark C. Markowski, M.D., Ph.D.
Prostate Cancer Program
The Skip Viragh Outpatient Cancer Building
Johns Hopkins University
201 N. Broadway, Room 9160, Mailbox 7
Baltimore, MD 21287
Tel 410-614-0567
Fax 410-367-2668
Email: mmarko12@jhmi.edu

Co-Principal investigator
Emmanuel S. Antonarakis, M.D.
Prostate Cancer Program
The Skip Viragh Outpatient Cancer Building
Johns Hopkins University
201 N. Broadway, Room 9129, Mailbox 7
Baltimore, MD 21287
Tel 410-502-7528
Fax 410-367-2668
Email: eantona1@jhmi.edu

Statistician
Hao Wang, Ph.D.
Wei Fu, MS
Division of Biostatistics & Bioinformatics
Sidney Kimmel Comprehensive
Cancer Center at Johns Hopkins
Johns Hopkins University, School of Medicine
Baltimore, MD, USA
Tel 410-614-3426
Tel 410-955-4884
Email: hwang76@jhmi.edu; wfu@jhu.edu

Lead Study Pathologist
Angelo De Marzo M.D., Ph.D.
Department of Pathology
Johns Hopkins University,
School of Medicine
1650 Orleans Street
CRB2, Room 144
Baltimore, MD 21231
Tel 410-614-5686
Email: ademarz@jhmi.edu

Project Manager
Aliya Lalji
The Skip Viragh Outpatient Cancer Building
Johns Hopkins University
201 N. Broadway, Mailbox 7
Baltimore, MD 21287
Tel 410-502-5101
Fax 410-367-2668
Email: alalji1@jhmi.edu

Protocol Version 8.0 Protocol Date: June 9, 2021

CONFIDENTIALITY STATEMENT

The information in this document is provided to you as an investigator, potential investigator, consultant, or contractor, for review by you, your staff, and the appropriate Institutional Review Board or Ethics Committee. By accepting this document, you agree that the information contained herein will not be disclosed to others without written authorization from the lead site/sponsor, except to the extent necessary to initiate the study or conduct study-related activities.

INVESTIGATOR APPROVAL OF PROTOCOL

COMbination of Bipolar Androgen Therapy and Nivolumab in Patients with Metastatic Castration-Resistant Prostate Cancer

[COMBAT-CRPC]

Protocol Version: **V. 8.0**

Version Date: **6/9/2021**

Principal Investigator Signature: ______________________________

Principal Investigator Name: ______________________________

Date of Signature: ______________________________

SYNOPSIS

Title
COMbination of Bipolar Androgen Therapy and Nivolumab in Men with Metastatic Castration-Resistant Prostate Cancer [COMBAT-CRPC]

Lead site
Johns Hopkins University

Sponsor
Bristol-Myers Squibb

IND holder
Mark C. Markowski, MD, Ph.D

Investigational agent
Testosterone Cypionate 400mg IM every 28 days; Nivolumab 480mg IV every 4 weeks

Phase
2

Target population

- Adult male > 18 years of age
- Histologic or cytologic diagnosis of adenocarcinoma of the prostate.
- Known castration-resistant disease, defined according to PCWG3
- Metastatic disease as defined for ≥ 1 bone metastases confirmed by bone scintigraphy or radiographic soft tissue metastasis. Pelvic lymph nodes will be considered as metastatic disease if they measure ≥ 15 mm in short-axis diameter.
- Soft tissue lesion available for biopsy collection.
- PSA and/or radiographic progression on at least ONE prior second generation AR-targeted therapies (i.e. abiraterone acetate, enzalutamide); and up to ONE prior chemotherapy agent for mCRPC is permitted.
- Serum PSA at screening ≥ 2.0 ng/mL
- Serum testosterone level ≤ 50 ng/dL at time of screening
- Life expectancy ≥ 6 months
- ECOG performance status ≤ 2
- Adequate bone marrow, renal and liver function (ANC > 1.0K, Plt > 100K, Hgb ≥ 9 g/dL; CrCl ≥ 40 mL/min; AST/ALT WNL; Total Bilirubin WNL).

Study centers
Two participating sites in the United States, parts of the PCCTC network.

Start date/Duration
First patients are expected to be enrolled in Q3/Q4 2018. Accrual is estimated to last 18 months with up to 24 months of follow-up after the last patient has been entered.

Expected enrollment
44 patients

Rationale
Rationale for Combination of High-Dose Testosterone with Nivolumab in Metastatic Castration Resistant Prostate Cancer (mCRPC) Patients

Bipolar Androgen Therapy (BAT) alone: BAT is a paradoxical approach for the treatment of mCRPC whereby testosterone levels are rapidly cycled between supraphysiologic and near-castrate concentrations. Based on prior studies, the incidence of germline mutations in DNA-repair genes in mCRPC patients is ~12% with somatic DNA-repair mutations approaching 20-25%. Interestingly, we have identified a correlation between long-term ("exceptional") responders to BAT and the presence

of mutations in homologous-recombination DNA repair genes. In Teply et al. (*Eur Urol*, 2017), we report the case of a patient with mCRPC who achieved a durable (>24 mo) complete radiographic and serologic response following treatment with BAT. DNA sequencing found inactivating mutations in both *BRCA2* and *ATM*, which were confirmed on germline DNA testing. We are currently sequencing tumor/germline DNA from other "exceptional" responders to BAT, and remarkably, we have found DNA repair mutations in 5/6 extreme responders. These unpublished findings suggest a relationship between responses to BAT and the presence of mutations in DNA-repair genes.

Checkpoint Blockade alone: Two randomized phase III trials involving single-agent checkpoint (CTLA-4) inhibitors in mCRPC patients did not meet their primary endpoints. However, a small subset (~20%) of mCRPC patients did derive clinical benefit. In pilot studies conducted at Johns Hopkins, we have observed dramatic responses to checkpoint inhibition in those patients with DNA-repair defects. For instance, two patients (#1: *MSH6*-mutated; #2: *BRCA2*-mutated) achieved a PSA_{50} response following combined PD-1/CTLA-4 checkpoint inhibition. A third mCRPC patient (#3: *ATM*-mutated) achieved a partial radiographic RECIST response. A fourth patient (#4: *POLH*-mutated) also had a RECIST response. Similar to the efficacy of checkpoint inhibitors in patients with MSI-High colon cancer, we speculate that a benefit of immunotherapy may be observed in those mCRPC patients with germline and/or somatic mutations in DNA-repair genes.

BAT/Immunotherapy Combination: Even more notably, we have observed two mCRPC patients with intact DNA repair processes (i.e. no repair gene mutations) that were treated initially with BAT and subsequently achieved dramatic PSA/RECIST responses to PD-1 checkpoint inhibition. Patient #1 achieved a PSA_{50} response to BAT which lasted 11 months before developing subsequent PSA progression. Upon starting nivolumab in combination with an anti-CD73 inhibitor, his PSA level dropped from 692 ng/mL to 3 ng/mL in the first 12 weeks with an associated clinical benefit. Patient #2 also achieved a PSA_{50} response to BAT lasting 6 months. Following subsequent treatment with checkpoint inhibition, his PSA dropped from 56 ng/mL to 4 ng/mL with a partial radiographic response after only 3 months of therapy. As mentioned, neither patients had a DNA-repair mutation although both benefited dramatically from PD-1 inhibition. Further study of BAT with nivolumab is warranted.

Objectives

Primary:
To estimate the PSA_{50} response rate, defined as a $\geq$ 50% decline in PSA from baseline, confirmed with a second measurement at least 4 weeks apart (PCWG3).

Secondary:
- To estimate the median PSA progression-free survival (PSA-PFS)(PCWG3).
- To estimate the median progression-free survival (PFS)(PCWG3).
- To estimate the durable progression-free survival (PFS > 6 months)
- To estimate the objective response rate (ORR)
- To estimate median overall survival (OS)
- To determine the safety and tolerability of BAT/Nivolumab in the mCRPC population.

Correlatives/Tertiary:

- To estimate the percentage of patients with somatic (tumor) or germline (inherited) mutations in homologous repair (HR) and/or mismatch repair genes.
- To measure IHC-based changes in gamma-H2AX, RAD51, 53BP1 (DNA damage markers) following treatment with BAT and at progression on BAT/nivolumab.
- To measure changes in CD4, CD8, FOXP3, Ki-67, PD-1, PD-L1 tumor levels with BAT alone and at progression following BAT/Nivolumab.
- To investigate any association between PD-1 and PD-L1 expression in tumor specimens and PSA_{50} response rates.
- To explore the relationship between mutation-associated neoantigen (MANA) and tumor-associated neoantigen (TAA) assays and clinical outcomes.
- To characterize the effect of BAT on production of inflammatory chemokines and cytokines.

Study design

The study is an open-label, single arm, Phase II trial. Eligible patients are men with mCRPC. All patients must have a rising PSA and/or radiographic progression and prior treatment with at least one novel AR targeted therapy (i.e. abiraterone acetate, enzalutamide), and up to one prior chemotherapy agent for mCRPC is permitted. A mandatory tumor biopsy will be performed prior to the start of therapy in all patients. A second, mandatory tumor biopsy will take place after three months of therapy with BAT alone. A third, OPTIONAL biopsy will be performed following PSA and/or radiographic progression on BAT/Nivolumab.

After enrollment, patients will be treated with testosterone cypionate 400mg IM every 4 weeks for a lead-in period of 12 weeks. Patients will be followed monthly with clinic visits, safety labs (including CBC w/ Diff, CMP), PSA, and toxicity assessments. After the lead-in period, all patients will be treated with nivolumab 480mg IV every 4 weeks and maintained on testosterone cypionate 400mg IM every 4 weeks. Treatment [with a minimum drug exposure of 12 weeks] will be continued until PSA progression (PCGW3 criteria) or clinical/radiographic progression (whichever comes first), or until unmanageable toxicity requiring drug cessation. Patients will continue a GnRH analogue during the study or have undergone orchiectomy.

The trial schema:

Criteria for evaluation

Primary Endpoint
- PSA_{50} response rate, defined as a ≥ 50% decline in PSA from baseline, confirmed with a second measurement at least 4 weeks later (PCWG3).

Secondary Endpoints
- Safety/Tolerability, defined as incidence of CTCAE Grade ≥ 3 toxicities experienced by patients on the trial.
- PSA progression-free survival (PSA-PFS), defined as the time from initiation on testosterone therapy until PSA increase of 25%, confirmed with another measurement at least 4 weeks later (PCWG3).
- Progression–free survival (PFS)(PCWG3), defined as the time to radiographic or clinical progression or death, whichever comes first. Based on RECIST version 1.1 and PCWG3 definitions including: Progression of soft tissue lesions according to RECIST 1.1; Progression of bone lesions detected with bone scan according to PCWG3 criteria; Radiologically-confirmed spinal cord compression or pathological fracture due to malignant progression, or other clinical event deemed to be cancer-related.
- Objective response rate (ORR), defined as the proportion of patients achieving a complete/partial response in target lesions (RECIST 1.1).
- Durable Progression-free survival (Durable PFS), defined as a lack of clinical/radiographic progression for ≥ 6 months.
- Median Overall Survival (OS), defined as the time from study enrollment to death from any cause up to 2 years after the last dose of study treatment received.

Exploratory Endpoints

- PSA_{50} response, in patients with and without a somatic or germline HR or MMR gene mutation.
- To associate PSA response to BAT/Nivolumab with changes in gamma-H2AX, RAD51, 53BP1 formation in tumor tissue compared to baseline.
- To associate PSA_{50} response to BAT/Nivolumab with changes in CD4, CD8, FOXP3, PD-1, PD-L1 levels in tumor tissue compared to baseline.
- To associate clinical responses to BAT +/- Nivolumab with the generation of mutation-associated neoantigens. (MANAs) or tumor-associated neoantigens (TAAs).
- To characterize the effect of BAT on production of inflammatory chemokines and cytokines.

Exploratory Analyses

Estimate the Percentage of Patients with a Pathogenic Somatic or Germline Mutation in a HR or MMR Gene Mutations:
The somatic DNA mutations present in the tumor will be identified through Foundation Medicine sequencing. Germline DNA mutation testing per standard of care practice will be done using clinical-grade testing in a CLIA-certified laboratory prior to the start of treatment for consenting subjects only. Patients with a germline and/or somatic deleterious alteration in the pre-specified gene list (BRCA1, BRCA2, ATM, CHEK2, NBN, RAD50, RAD51C, RAD51D, PALB2, MRE11, FANCA, FANCB, FANCC, FANCD2, FANCE, FANCF, FANCG, FANCI, FANCL, FANCM, MLH1, MLH3, MSH2, MSH3, MSH6, PMS1 and PMS2) will be considered biomarker positive. For biomarker positive and biomarker negative subjects, we will calculate PSA_{50} response rates with confidence intervals for hypothesis generation.

PD-1, PD-L1, FOXP3, CD4, CD8, γH2AX, RAD51, 53BP1 Protein Levels:
Subjects will have pretreatment, on-treatment (and at progression) tumor biopsy specimens analyzed. IHC staining for PD-1 and PD-L1 will be performed on the pretreatment samples and scored as <1%, 1-5%, 5-10%, >10% and the results will be associated with responses for both proteins, separately, using descriptive statistics and Fisher's Exact Tests. Immunofluorescence will be used to quantify the median intensity of each gamma-H2AX, RAD51 and 53BP1 foci per cell as well as determine the median number of foci per cell on the pretreatment biopsies. Using analytical microscopy, Drs. Meeker and Heaphy have developed imaging software, which can isolate single nuclei and quantify both the intensity and number of immunofluorescent foci per nucleus (i.e. per cell). Using FFPE tissue obtained at biopsy, we will examine the change in DNA damage foci number and intensity (reported as median percent change) induced by BAT/Nivolumab in PSA_{50} responders versus non-responders using a T-test. Using imaging software, we will count the number of CD4+ and CD8+ lymphocytes in pre-treatment and on-treatment biopsies. We will correlate PSA_{50} responses with CD4+ and CD8+ lymphocyte tumor infiltration.

Interrogate for Mutation-associated Neoantigens (MANAs) and Tumor-associated Neoantigens (TAAs):
Whole-exome sequencing will be performed on pre-treatment samples, after 12 weeks of BAT lead-in therapy, and post-progression tumor and matched normal samples. Exome data will be applied in a neoantigen prediction pipeline that evaluates antigen processing, MHC binding, and gene expression to generate neoantigens specific to the patient's HLA haplotype. Truncal neoantigens will be identified by correcting for tumor purity and ploidy. Putative-neoantigens will then be used to generate peptides and stimulate autologous T cells, followed by TCR next-generation sequencing.

To Characterize the Effect of BAT on Production of Inflammatory Chemokines and Cytokines:
Milliplex human cytokine/chemokine Immunology Multiplex Assay (Millipore-Sigma) will be used to assay acute effects of BAT on production of cytokines and chemokines. Plasma samples are obtained at screening, 3 hours after the first testosterone injection, 3 days after the first testosterone injection, Cycle 2 Day 1, and Cycle 7 Day 1. Analysis will be performed by the Immune Processing Core Lab at Johns Hopkins.

Statistical method

Primary Analysis
The primary endpoint of this study is PSA_{50} response, defined as a decrease in the PSA to ≥ 50% less than the baseline PSA upon enrollment in the trial. The decrease must be confirmed by a second measurement at least 4 weeks apart. For purposes of meeting the primary endpoint, patients will be considered to have done so if they have a PSA_{50} response either while on therapy with BAT alone (during lead-in) or BAT in combination with Nivolumab (combination phase). PSA values will be measured monthly during the trial. All patients who take at least one dose of BAT (during lead-in) will be considered evaluable for the primary endpoint. If patients do not have at least one follow-up PSA after initiation of BAT due to stopping therapy for toxicity or withdrawing consent, then they will be replaced. PSA_{50} response rate will be estimated along with 95% confidence interval.

Secondary Analysis
Safety:
Patients will be assessed for toxicities at each clinical evaluation. Toxicities will graded according to current CTCAE standardized grading scales. The incidence of grade 3-5 toxicities will be reported to the lead site and to BMS. Patients will be assessed for toxicity as long as they are taking BAT alone or BAT in combination with Nivolumab, and patients will continue to be followed if treatment is discontinued for toxicity until the toxicities improve to grade 1 or resolve. Toxicities will be reported as a tabulated table by type and grade.

PSA Progression-free Survival (PSA-PFS):
A standard definition of PSA progression per PCWG3 will be used. PSA-PFS will be defined as an increase in 25% over a nadir value, confirmed by a follow-up PSA at least 4 weeks later. If patients are removed from the study prior to PSA progression, then they will be censored at that time. We will use the Kaplan-Meier method to summarize the median PSA-PFS.

Progression-free Survival (PFS):

Progression-free survival will be measured from the time of the first dose to objective clinical or radiographic tumor progression as defined by PCWG3 for progressive disease or death, and summarized using a Kaplan-Meier curve. Progression will be assigned to the earliest observed time. Patients whose disease has not progressed at follow-up will be censored at the date when the last tumor assessment determined a lack of progression. We will use the Kaplan-Meier method to summarize the median PFS.

Objective Response Rate (ORR):
The objective response rate is defined as the percentage of patients who achieve an objective response by RECIST 1.1 criteria (i.e. Complete response or Partial Response) to BAT alone or BAT in combination with Nivolumab. We will estimate the objective response rate, along with the exact 95% confidence interval, for the population of patients with RECIST-evaluable disease.

Durable Progression-free Survival (durable-PFS):
Durable PFS will be defined as the proportion of patients without clinical or radiographic progression, as defined by PCWG3, or death for at least 24 weeks from the start of treatment.

Overall Survival (OS):
Overall survival will be defined as the time from study enrollment to death from any cause up to 2 years after the last dose of study treatment. This will be summarized using a Kaplan-Meier curve.

Sample Size/Power Calculation:
The sample size is calculated to detect an improved PSA_{50} response rate from 25% (null hypothesis) to 45% (alternative). A minimax Simon two stage design is planned. A total of 23 patients will be entered in the first stage. A planned analysis will be performed 12 weeks after the last patient in the first stage receives the first dose of nivolumab. If ≤ 5 subjects have a PSA_{50} response, the study will be terminated and we will conclude the regimen is ineffective (i.e. not more effective than BAT alone). If ≥ 6 subjects respond in the first stage, then an additional 16 patients will be enrolled, for a total of 39 patients. If a total of ≤ 13 subjects respond in stage one and two combined, we consider this regimen ineffective. If a total of ≥ 14 respond, we will conclude that the regimen is promising and warrants further study. A total of 44 patients will be enrolled to account for a 10% possible dropout rate and unevaluable patients.

This design provides 90% power to detect an absolute 20% increase in PSA response rate with a one-sided type I error of 0.1. The chance of early stopping for futility is 0.49 if PSA_{50} response rate is 25%.

Safety analysis

Standard safety summaries will be provided for treatment exposure, patient disposition, adverse events leading to discontinuation, serious adverse events, and all events resulting in death, including those up to 100 days after treatment discontinuation. The incidence of adverse events will be tabulated and reviewed for potential significance and clinical importance.

TABLE OF CONTENTS

1. INTRODUCTION **13**
1.1 Disease Background 13
1.2 Treatment Background 14
1.3 Rationale 24
2.1 Primary Objective 28
2.2 Secondary Objectives 28
2.3 Correlative/Exploratory/Tertiary Objectives 28
3. PATIENT SELECTION **29**
3.1 Target Population 29
3.2 Expected Enrollment 29
3.3 Inclusion Criteria 29
3.4 Exclusion Criteria 30
4. PATIENT REGISTRATION AND ENROLLMENT PLAN **33**
4.1 Registration Procedure 33
5. TREATMENT/INTERVENTION PLAN **33**
5.1 Screening Assessment 36
5.2 Testosterone Lead-in Period 38
5.3 Testosterone/Nivolumab Combination Period 39
5.4 End of Treatment/Treatment Discontinuation Visit 40
5.5 Follow-up 40
5.6 Correlative/Special Studies 41
6. STUDY DRUGS 41
6.1 Description of Treatments 41
6.2 Administration, Supply, and Storage 41
7. DOSE ADJUSTMENT AND DELAY, TREATMENT DISCONTINUATION, WITHDRAWAL, AND TERMINATION CRITERIA **42**
7.1 Dosing and Dose Modifications 42
7.2 Dose Delay and Treatment Discontinuation 43
7.3 Dose Delay Criteria 43
7.4 Criteria to Resume Treatment 43
7.5 Discontinuation Criteria 44
7.6 Treatment of Nivolumab-Related Infusion Reactions 45
7.7 Treatment Beyond Disease Progression 46
7.8 Immunotherapy Adverse Event Management 47
7.9 Removal of Subjects from the Study, Therapy Assessment 47
7.10 Treatment Compliance 48
7.11 Destruction of Study Drug 48
7.12 Return of Study Drug 48
8. ADVERSE EVENTS **49**
8.1 Definitions 49
8.2 Expectedness 52
8.3 Recording and Grading 52
8.4 Reporting Adverse Events 53
9. CRITERIA FOR OUTCOME ASSESSMENT/THERAPEUTIC RESPONSE **56**
9.1 Outcome Assessment 56
9.2 Therapeutic Response 57
9.3 Response Criteria for Primary and Secondary Endpoints 59
9.4 Criteria for Progressive Disease 61
10. DATA REPORTING AND REGULATORY REQUIREMENTS **61**
10.1 Data Entry 62
10.2 Data Management 63
10.3 Study Monitoring and Quality Assurance 63

10.4 Clinical Trial Agreement 64
11. STATISTICAL CONSIDERATIONS 64
11.1 Study Endpoints 65
11.2 Analysis Populations 67
11.3 Safety Analysis 68
12. PROTECTION OF HUMAN SUBJECTS 68
12.1 *Ethical Considerations* 68
12.2 *Protocol Amendments* 68
12.3 *Written Informed Consent* 68
12.4 *Protection of Privacy* 69
12.5 *Terminating or Modifying the Study* 69
13. REFERENCES 71

APPENDIX A: MANAGEMENT ALGORITHM FOR IMMUNO-ONCOLOGY AGENTS 75
APPENDIX B: PERFORMANCE STATUS CRITERIA 83
APPENDIX C: LABORATORY MANUAL 84
APPENDIX D: GLOSSARY OF ABBREVIATIONS AND ACRONYMS 87
APPENDIX E: ACCEPTABLE BIRTH CONTROL METHODS 93
APPENDIX F: HY'S LAW 94
APPENDIX G: TUMOR SAMPLE INFORMATION FORM 97

List of Tables:

Table 1 Somatic Gene Analysis of 7 mCRPC Patients Treated with Bipolar Androgen Therapy 25
Table 2 Study Calendar 34
Table 3 Relationship of Adverse Event to Study Drug 53
Table 4 RECIST 1.1 Response Criteria for Target Lesions 59
Table 5 RECIST 1.1 Response Criteria for Nontarget Lesions 60
Table 6 Categories for Response to Treatment 67

List of Figures:

Trial Schema 7
Figure 1 Clinical States of Prostate Cancer 13
Figure 2 Androgen Induced Double Strand DNA Breaks in Prostate Cancer Cell Lines Stimulated with High Levels of Androgen. 15
Figure 3 Castrated NOG Mice Inoculated with LNCaP/A-cells Were Either Exposed to BAT Therapy or Left in a Permanently Castrate State. 16
Figure 4 Clinical Trial of BAT Plus Etoposide. 19
Figure 5 Results from the Pilot BAT Study in Men with CRPC. 20
Figure 6 PSA Values of Extreme Responder Patients Treated with Bipolar Androgen Therapy 24
Figure 7 Waterfall Plot of Maximal PSA Change in mCRPC Patients Treated with Combination Checkpoint Inhibitor Therapy 26
Figure 8 CT Imaging of Post BAT mCRPC Patients Before and During Treatment with Checkpoint Inhibition 27

1. INTRODUCTION

1.1 Disease Background

Prostate cancer is the most commonly diagnosed non-cutaneous malignancy in men, with an estimated 180,000 cases annually in the United States (1). It is the second most common cause of cancer mortality in the United States as well, with over 26,000 deaths in 2016 (1). The discrepancy between the incidence and mortality numbers demonstrate its potential curability if treated while disease is local, as well as the non-lethal nature of some cancers, even if not treated definitively. While many men are cured of their disease, many others will unfortunately progress to incurable and lethal metastatic disease.

1.1.1 Clinical States of Prostate Cancer

The course of prostate cancer from diagnosis to death is best categorized as a series of clinical states (Fig. 1). These states are defined by the extent of disease and status of responsiveness to hormonal therapy. Therapies have been developed for specific states, as each state presents unique risks to the patient and different responsiveness of the disease to therapy.

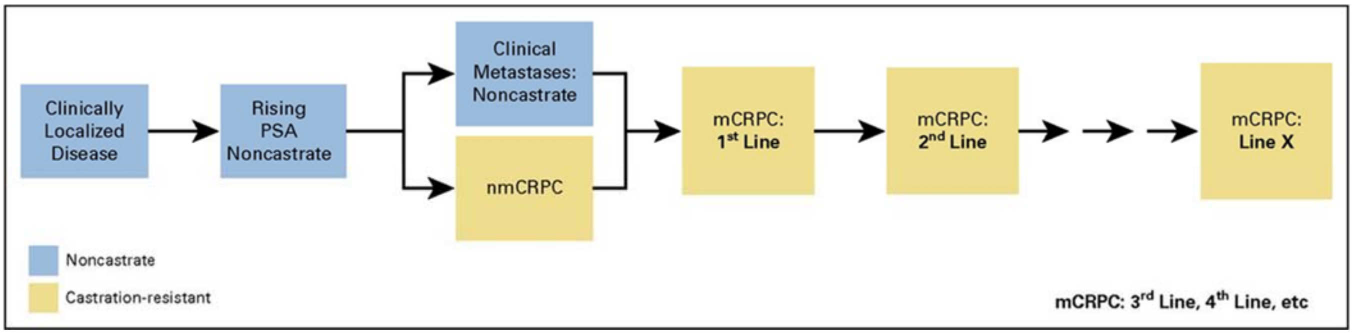

Figure 1. Clinical states of prostate cancer (2)

1.1.2 Metastatic Castration-Resistant Disease State

Androgen deprivation therapy (ADT) is the backbone of treatment for metastatic prostate cancer, and while ADT is initially highly effective, prostate cancers invariably adapt to a low-testosterone environment, leading to castration resistance. Studies have demonstrated that castration-resistant prostate cancer (CRPC) remains dependent on AR signaling. Based on this understanding, several drugs have been developed and Food and Drug Administration (FDA)-approved for the treatment of mCRPC, including abiraterone acetate and enzalutamide. However, we have observed in multiple clinical studies a paradoxical effect of high dose testosterone resulting in both PSA and radiographic responses in patients with mCRPC.

1.2 Treatment Background

1.2.1 Description and Mechanism of Action

Testosterone cypionate is the oil-soluble form of the androgenic hormone, testosterone. A paradoxical inhibition of cell growth has been observed in both androgen-sensitive and CRPC cell lines following the addition of high-dose testosterone.(3,4) Several mechanisms underlying this paradoxical response to androgens have been identified. First, our group and others have showed that AR may be involved in DNA relicensing during cell cycle progression.(5-7) Under normal conditions, AR is degraded during the cell cycle. Our group found that the AR protein is stabilized in the presence of supraphysiologic levels of androgen and remains bound to origins of replication.(5-7) This binding inhibits progression through the cell cycle, leading to cell death. Second, androgen-starved prostate cancer cells up-regulate both full-length AR and AR splice variants as a survival mechanism. The addition of high-dose androgen to the growth media results in the down-regulation of these AR splice variants (including AR-V7), potentially re-sensitizing these cells to ADT.(8,9) Third, we have shown that following androgen starvation, exposure of CRPC cells to high-dose androgen induces double-strand (ds) DNA breaks, resulting in cell growth inhibition and decreased clonogenic survival.(10) Mechanistically, high-dose androgen has also been shown to induce structural genomic rearrangements/translocations in CRPC cells, including the TMPRSS2-ERG fusion.(11)

Nivolumab (also referred to as BMS-936558, MDX1106, or ONO-4538) is a human monoclonal antibody (HuMAb; immunoglobulin G4 [IgG4]-S228P) that targets the programmed death-1 (PD-1) cluster of differentiation 279 (CD279) cell surface membrane receptor. PD-1 is a negative regulatory molecule expressed by activated T and B lymphocytes. Binding of PD-1 to its ligands, programmed death–ligands 1 (PD-L1) and 2 (PD-L2), results in the down-regulation of lymphocyte activation. Inhibition of the interaction between PD-1 and its ligands promotes immune responses and antigen-specific T-cell responses to both foreign antigens as well as self-antigens. Nivolumab is expressed in Chinese hamster ovary (CHO) cells and is produced using standard mammalian cell cultivation and chromatographic purification technologies. The clinical study product is a sterile solution for parenteral administration.

OPDIVO (nivolumab) is approved for the treatment of several types of cancer in multiple regions including the United States (US, Dec-2014), the European Union (EU, Jun-2015), and Japan (Jul-2014). Nivolumab is also being investigated in various other types of cancer as monotherapy or in combination with other therapies, and as single-dose monotherapy for the treatment of sepsis.

1.2.3 Preclinical Studies

High-dose testosterone, also referred to as Bipolar Androgen Therapy (BAT):
The mechanisms underlying the growth-suppressive effects of high levels of androgens in prostate cancer cells in vitro and in vivo is likely highly complex. Recent evidence from our group suggests that one mechanism may involve the formation of androgen-induced Topoisomerase II beta (TOP2B)-mediated double strand breaks at AR target genes (Figure 2).(10) Studies have shown that estrogen signaling in breast cancer cells involves the co-recruitment of Estrogen receptor and TOP2B to estrogen receptor target sites, where

TOP2B introduces transient double strand breaks. We hypothesized that such a mechanism may be involved in androgen signaling in prostate cancer cells and that at high doses of androgens, such breaks may persist and ultimately lead to growth suppression. In support of this, we observed that stimulation of androgen-deprived LNCaP cells with dihydrotestosterone (DHT) led to recruitment and catalytic activity of TOP2B at AR target sites in the TMPRSS2 enhancer as well as at other known AR target sites. At high doses of DHT, this TOP2B recruitment and catalytic activity was associated with significant formation of AR and TOP2-dependent persistent double strand breaks at the TMPRSS2 gene, as observed by fluorescence in situ hybridization (FISH) assay which is capable of detecting genomic breaks on an individual cell basis.(10) Such breaks likely occurred throughout the genome at AR target sites since we observed numerous γH2A.x foci, a marker for double strand break formation, throughout the nucleus in response to stimulation of LNCaP cells with high-dose DHT (Figure 2). In further confirmation of this, we also observed recruitment of ATM, a double strand break repair signaling protein, to AR target sites in PSA and TMPRSS2, genes present on different chromosomes in the cell. These findings suggest that exposure of prostate cancer cells from patients with CRPC to high doses of testosterone may induce growth suppression due to the accumulation of androgen-mediated, TOP2-induced double strand DNA breaks.(10)

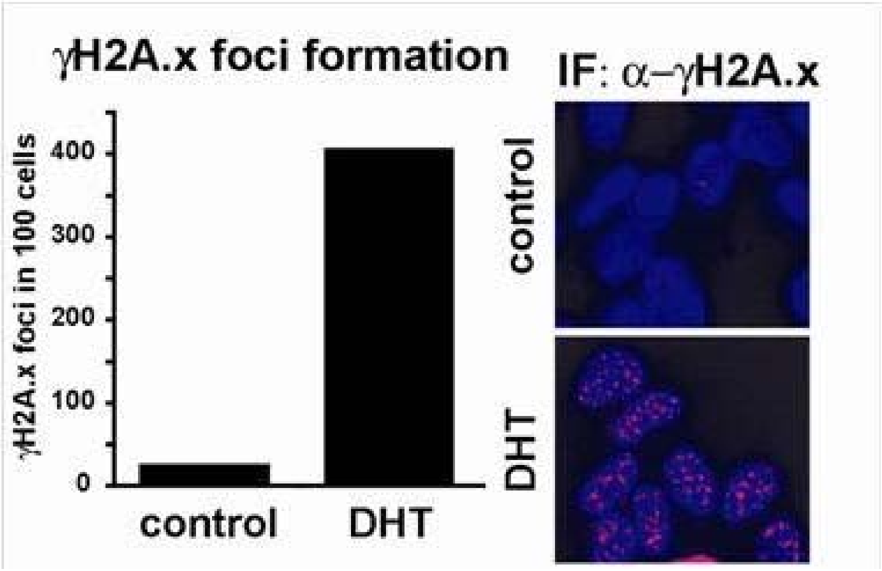

Figure 2. Androgen-induced double strand DNA breaks in prostate cancer cell lines stimulated with high levels of androgen. Stimulation of androgen-deprived LAPC4 cells (control) to high levels of DHT (DHT) leads to numerous double strand breaks throughout the nucleus as evidenced by the accumulation of numerous γH2A.x foci, a marker for formation of double strand breaks.

Recently, the Denmeade laboratory has explored a mechanism for growth inhibition by testosterone (T) in CRPC cells. These studies document that the increase in AR expression observed in these cells in the low T environment creates a unique therapeutic vulnerability to selectively kill CRPC cells. This is based upon the fact that AR is involved in DNA relicensing and DNA replication and AR must be degraded in each cell cycle for proper relicensing to occur. Overstabilization of the increased levels of AR observed in CRPC with supraphysiologic testosterone prevents complete degradation of AR via the proteasome during mitosis. This was demonstrated by in vivo treatment of resistant human LNCaP prostate cancer xenografts with T implants to achieve supraphysiologic serum T- levels.(5) This treatment resulted in significant growth inhibition (Figure 3a). These growth-inhibited cells had similar amount of cells with nuclear AR in the nucleus and Ki-67 positivity (Figure 3a). However, in xenografts treated with supraphysiologic T, the Cell Death Index was ~ 3-fold higher.(5) More strikingly, the percent of cells staining positive for AR in mitosis was approximately 10-fold higher in cells exposed to supraphysiologic T versus castrate-only animals (Figure 3b). This data suggests that CRPC cells that have not properly relicensed DNA, can die when they attempt to proceed through a subsequent cell cycle. Thus, based on this proposed mechanism, prostate cancer cells that maintain high AR levels will be vulnerable to cell death when exposed to supraphysiologic T conditions due to their inability to rapidly auto-regulate AR to lower levels. Due to the bipolar cycling between high and low serum T achieved with BAT, those cells that do manage to survive the high T environment through adaptive down-regulation of AR will become vulnerable to cell death when suddenly exposed to low T conditions that occur over the cycle of BAT.

A.

	Castrate	Castrate + T implants
Tumor Volume (cc)	0.98 ± 0.17	0.32 ± 0.06*
PSA/gram tumor	24.5 ± 9.2	95.5 ± 5.4*
Ki-67	>75%	>75%
Mitotic Index	0.51 ± 0.15%	0.32 ± 0.23%
AR in Nucleus	>90%	>90%
Cell Death Index	15 ± 8%	39 ± 9%*
AR in Mitosis	7 ± 6%	77 ± 8%*

* p<0.05

B.

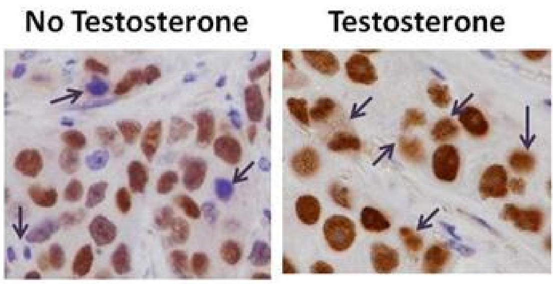

Figure 3. Castrated NOG mice inoculated with LNCaP/A-cells were either exposed to BAT therapy (via an implanted testosterone filled capsule that was placed and removed at two week intervals) or left in a permanently castrate state (diamond versus box). (A) Evaluation of indicated parameters in LNCaP/A- cells growing in castrate mice vs. castrate mice supplemented with subcutaneous testosterone-filled silastic implants. (B) Immunohistochemical staining for AR in harvested LNCaP/A- xenografts growing in castrate vs. castrate + T-pelleted mice. Blue arrows indicate mitotic figures.

Nivolumab:

Tumors can express tumor-specific antigens as a result of mutational burden, and ongoing immune surveillance is believed to control the development of many tumors. Tumor progression may depend on the acquisition of mechanisms that permit them to evade an effective immune response. One such mechanism of evasion may be the expression of ligands, which engage inhibitory receptor(s) on anti-tumor T cells of many tumors. PD-L1

expression has been found on a number of tumors and may be a mechanism by which tumors can directly engage PD-1 to evade an effective anti-tumor immune response.(12-14) Expression of IFN-γ by activated T cells is known to induce PD-L1 expression in tumors.(15) PD-L1 expression has been associated with poor prognoses in renal,(16-18) esophageal,(19) gastric,(20) ovarian,(14) pancreatic,(21) and lung cancers.(22) PD-1 engagement on T cells by PD-L1-positive APC or PD-L1-positive tumor cells in the tumor microenvironment may limit effective immune responses. Conversely, PD-L1 expression may be a positive prognostic factor as it may indicate infiltration of tumor-specific T cells that secrete IFN-γ, which up-regulates PD-L1 expression. Consistent with this hypothesis is the co-localization of lymphoid cell infiltrates and PD-L1 staining observed in human melanoma lesions.(23)

Studies in multiple tumor models using a chimeric murine anti-mouse PD-1 antibody showed that PD-1 blockade has anti-tumor activity.(24) Blocking PD-1 in PD-L1-positive tumors may reverse the inactivation of tumor-specific effector T cells at the tumor site, as well as activate anti-tumor responses that are limited by PD-L1 expression on "host" DC or APC. The anti-tumor effects of anti-PD-1 observed in several murine models suggest that both PD-L1-positive and PD- L1-negative tumors may be targeted using this approach. In addition, in several tumor models in which anti-PD-1 has proved ineffective, PD-1 blockade can be combined with vaccines or other immunomodulatory antibodies for improved therapeutic efficacy.(25-27) PD-1 blockade by Nivolumab is a promising avenue to pursue as an anti-tumor therapy for recurrent or treatment-refractory malignancies.

1.2.4 Clinical Studies

BAT:
Up until recently, there had been very limited clinical experience in the PSA-era treating CRPC patients with testosterone. Brendler et al. (1949) at the Brady Urological Institute reported in the Archives of Surgery on the use of parenteral testosterone in several men with advanced CRPC.(28) They observed considerable improvement in several men that included decreased pain, decreased prostate size, and decreases in acid and alkaline phosphatase. In a second study, Prout and Brewer (1967) reported in Cancer on the treatment of men who had been either untreated or recently castrated or long term castrates with parenteral testosterone.(29) Five relapsed patients in the long term castrate group received testosterone for at least one month and 4 of 5 had subjective improvement. Five remaining patients in the long term castrate group received testosterone for 1-19 days, with each progressing and subsequently coming off therapy. Acid phosphatase declined in 2/5 men receiving a longer course of testosterone. Remarkably, one man in this group admitted to hospital with severe back pain, weakness, and anorexia had a 10 month response with complete cessation of pain, excellent appetite, and weight gain with a decrease in acid phosphatase from 50 to 5 units.

In contrast, a number of studies during the 1960-70s evaluated the use of T-priming in combination with 32P-sodium phosphate to treat men with CRPC and severe pain due to widespread bony metastases.(30,31) In these studies, initial T-priming using a variety of parenteral dosing regimens was associated with transient increase in bone pain during the first week followed by excellent pain palliation following administration of 32P. Similar results were observed in studies led by Manni who evaluated T- priming with chemotherapy in the 1980's.(32) These studies were also conducted in men with CRPC and

pain due to widely metastatic disease. In these studies, increased bone pain was also observed in men upon initial treatment with oral androgens. The increased pain in these studies typically occurred within days of T administration. Thus, given this time frame, it is likely the increased pain was due to T-stimulation of inflammation/cytokine release within sites of bone metastases rather than a direct effect on tumor growth. Such rapid change is also seen in men with bone pain upon initially starting ADT. Marked improvement in pain after ADT often occurs within hours of treatment, an effect not due to tumor death but rather a rapid change in expression of pain-promoting gene products.

Two contemporary Phase I studies were reported describing the results of the use of transdermal T as therapy for men with CRPC who had minimal to moderate disease burden and no pain due to prostate cancer. In the first study, Szmulewitz et al. (2009) evaluated the effect of increasing doses of transdermal T in 15 men with early CRPC (rising PSA and minimal bone disease).(33) Five men each were treated with 2.5, 5.0 or 7.5 mg/day of transdermal T which brought the median concentration of T from castrate to 305, 308, and 297 ng/dL respectively. In this study, no grade 3 or 4 toxicities were observed with the exception of one man who was taken off study at week 53 for grade 4 cardiac toxicity. Only one patient had symptomatic progression and three patients (20%) had a decrease in PSA (largest was 43%). Patients treated at the highest T dose had a prolonged time to progression that did not reach statistical significance, most likely due to the small cohort size. In the second study, Morris et al. (2016) evaluated the effect of transdermal T at a dose of 7.5 mg/day administered for 1 week (n=3), 1 month (n=3) or until disease progression (n=6) in 12 patients with CRPC.(34) They observed no grade 3 or 4 toxicities and no pain flares. Eugonadal serum T levels were reported for this study. No objective responses were observed. Four patients had at least 20% declines and one achieved a > 50% PSA decline.

Neither of these Phase I studies achieved the supraphysiologic levels of serum T that can be reached with FDA-approved doses of T administered as an intramuscular depot.(35) However, the levels of serum T achieved were in the high-end of the eugonadal range. Remarkably, although the studies were considered "negative" from the standpoint of disease response, in both studies the administration of parenteral T to men with CRPC was very well-tolerated and did not result in significant worsening of disease or symptoms, including pain flares. While only one patient out of 27 from the combined studies had a reported > 50% decline in PSA, smaller PSA declines were observed in a few of the patients on these two studies with a trend toward a dose-responsive effect, suggesting a potential for therapeutic benefit in some patients.(33,34)

A.

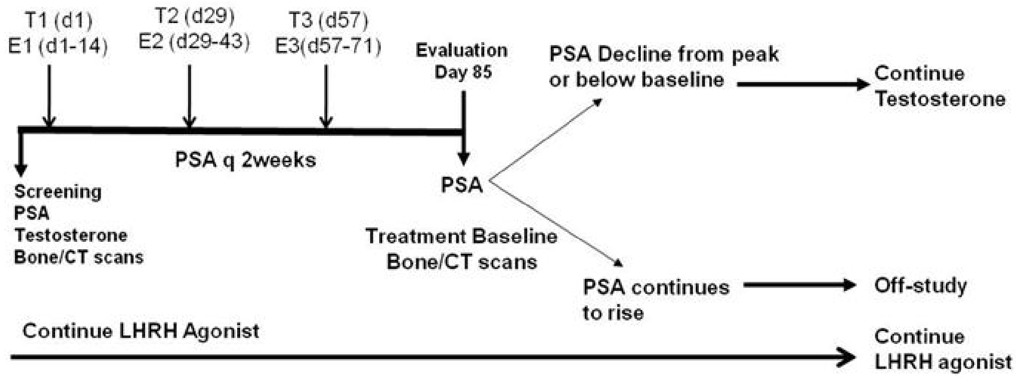

B.

Median age, yrs (range)	71 (56-87)
Median PSA, ng/mL (range)	20.0 (1.4-819.1)
Median testosterone, ng/dL (range)	<20 (<20-41)
Gleason score, N (%)	
6	4 (25)
7	7 (43.8)
8	3 (18.8)
9	2 (12.5)
Median length of continuous ADT, mos (range)	45.5 (12-146)
ECOG performance status, N (%)	
0	15 (93.8)
1	1 (6.3)
Number of 2nd line hormone therapies, N (%)	
0	2 (12.5)
1	9 (56.3)
2	3 (18.8)
3	2 (12.5)
Race, N (%)	
Caucasian	12 (75)
African-American	4 (25)
Patients with bone metastases, N (%)	3 (18.8)
Patients with RECIST evaluable soft tissue metastases, N (%)	10 (62.5)

C.

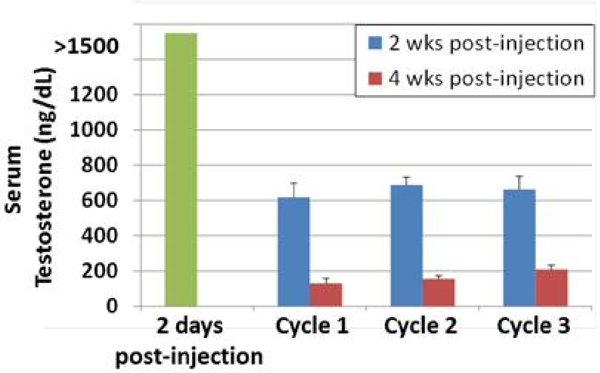

Figure 4. Clinical trial of BAT plus etoposide. (A) Schematic of study design. (B) Baseline characteristics of patients on study. (C) Mean serum testosterone levels at indicated time points for patients on study.

Based on the preclinical results and potential mechanisms for growth inhibition that include androgen-induced double strand breaks and the stabilization of AR preventing relicensing, we have conducted a pilot study evaluating the efficacy and safety of pharmacologic doses of testosterone to produce supraphysiologic T levels in conjunction with oral etoposide (E) in chronically castrated men with rising PSA and CRPC (Figure 4a).(36) Patients who had been continuously castrate for more than one year with minimal metastatic disease burden (≤ 5 total bone metastases and ≤ 10 total sites of metastases), and/or rising PSA were eligible (Figure 4b). To achieve rapid cycling between supraphysiologic and near-castrate serum T, patients received an intramuscular injection of 400 mg testosterone cypionate (i.e. BAT) every 28 days. For the first 3 cycles of therapy, patients received BAT plus oral etoposide 100 mg PO/day, days 1-14 of a 28 day cycle. After 3 cycles, PSA and objective responses were assessed. Those patients with a PSA that was declining from peak levels and no objective evidence of disease progression or worsening pain were continued on therapy. Given the toxicity associated with etoposide and the lack of clinical response in an earlier

trial,(37) patients who were responding after 3 cycles of testosterone plus etoposide were continued on testosterone alone based on protocol amendment.

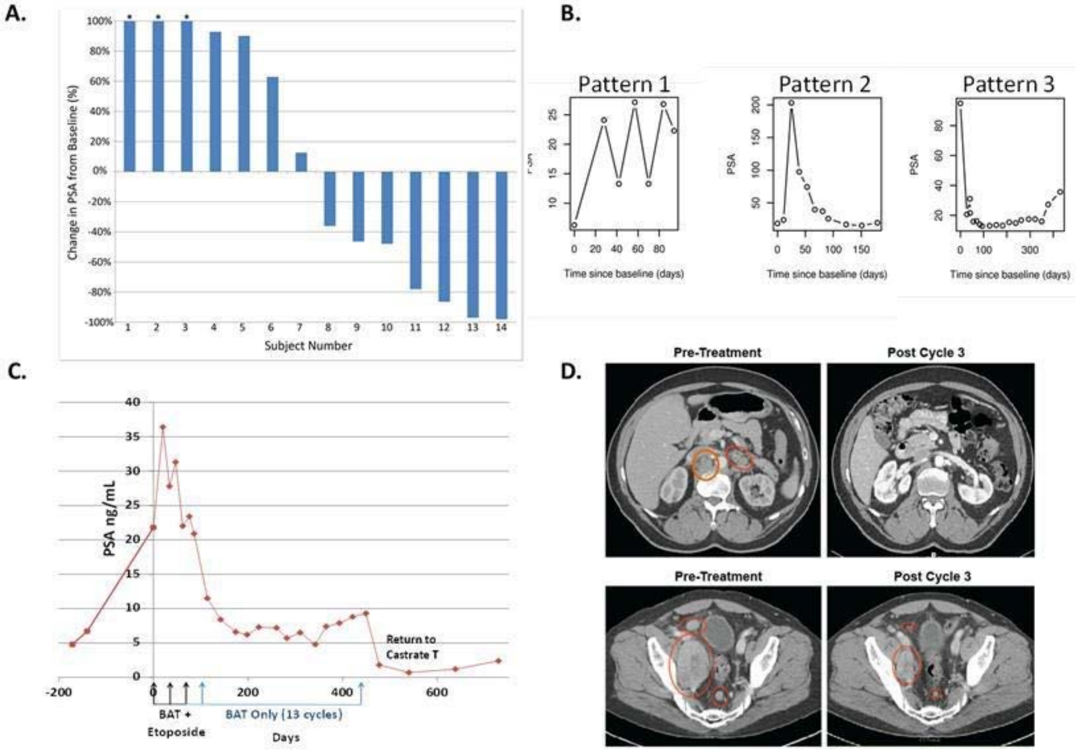

Figure 5. Results from the pilot BAT study in men with CRPC. (A) Waterfall plot of best PSA response with 7/14 men and 4/14 men achieving PSA declines > 30% and > 50%, respectively; (B) Representative patterns of PSA response in pilot study; PSA and serum T level in non-responder; (C) PSA response in patient receiving 3 cycles of T + E and then 13 additional cycles of T alone. Patient demonstrated renewed sensitivity to ADT after progressing on BAT; (D) Objective lymph node complete and partial response after 3 cycles of BAT + E.

Seven of fourteen patients had a decline in PSA from baseline value (Figure 5a). An eighth patient progressing after testosterone treatment for 6 months, had a decline in PSA upon reaching castrate-level testosterone. Non-responders came off trial after 3 cycles due to PSA progression. Overall, three patterns of PSA response were observed (Figure 5b). For the seven patients that had a PSA decline, the median time to PSA progression was 221 days (range, 95 to 454 days).

The dose of 400 mg testosterone cypionate produces supraphysiologic levels > 1500 ng/dL within 2 days post injection (Figure 4c). At baseline, ten subjects had RECIST-evaluable soft tissue metastases (Figure 4b). Of these patients, two (20%) had progressive disease (PD), three (30%) had stable disease (SD) after a median follow-up of 91 days (range, 87 to 92 days), four (40%) had partial responses (PRs), and one (10%) had a complete response

(CR) (Figure 5d). None of the 14 patients completing 3 months of therapy developed new bone metastases. One patient with > 50% decrease in PSA had intensification of an isolated tibial metastases on bone scan and was removed from study despite decline in PSA levels. No other patient developed worsening pain on study.

Two clinical trials involving high-dose T are ongoing at Johns Hopkins: 1. RESTORE Trial (J1416) - A Phase II Study to Determine Sequential Response to Bipolar Androgen Therapy (BAT) followed by Enzalutamide or Abiraterone Post-BAT in Men with Prostate Cancer Progressing on Combined Androgen Ablative Therapies; 2. TRANSFORMER trial (J14146) - A Randomized Phase II Study Comparing Bipolar Androgen Therapy vs. Enzalutamide in Asymptomatic Men with Castration-Resistant Metastatic Prostate Cancer.

<u>Nivolumab</u>
This updated Investigators Brochure (IB) references the most recent USPI and EU SmPC as the basis for the current state of knowledge on Nivolumab for use in humans with cancer. The approved USPI and SmPC are provided in Appendix 1 and Appendix 2 of the IB, respectively. The USPI and SmPC summarize Nivolumab monotherapy clinical data for melanoma (based on CA209037 and CA209066), SQ NSCLC (based on CA209063 and CA209017), NSQ NSCLC (based on CA209057), RCC (based on CA209025), Classical Hodgkin Lymphoma (cHL; based on CA209205 and CA209039), and urothelial carcinoma (UC; based on CA209275 and CA209032), as well as clinical data for Nivolumab in combination with Ipilimumab for melanoma (based on CA209004, CA209069, and CA209067). In addition, the USPI describes Nivolumab monotherapy clinical data for SCCHN (based on CA209141). Data from clinical studies that are relevant to ongoing clinical investigations in oncology that are not in the approved USPI and SmPC or to subjects with sepsis are included in this updated IB.
The PK, clinical activity, and safety of Nivolumab have been assessed in approximately 75 clinical studies sponsored by BMS or ONO. The description and status of studies with reference safety information are provided in Appendix 4 of the IB. Across those studies, approximately 16,900 subjects have received Nivolumab monotherapy in single- or multiple-dose Phase 1/2/3 studies or studies with Nivolumab in combination with other therapeutics (Ipilimumab, cytotoxic chemotherapy, anti-angiogenics, and targeted therapies). Results from the ongoing studies are preliminary and are subject to change.

Nivolumab has demonstrated clinical activity in NSCLC, melanoma, RCC, cHL, SCCHN, UC (approved indications) and other tumor types (Section 5.4 of the IB) as monotherapy or in combination with Ipilimumab or other therapeutics. The majority of responses were durable and exceeded 6 months. In randomized-controlled studies, Nivolumab monotherapy demonstrated statistically significant improvement in OS over standard-of-care in subjects with advanced or metastatic melanoma, subjects with advanced or metastatic NSCLC, subjects with advanced RCC, and subjects with recurrent or metastatic SCCHN. In randomized-controlled studies, Nivolumab in combination with Ipilimumab demonstrated statistically significant improvement in PFS and ORR over Ipilimumab monotherapy in subjects with advanced or metastatic melanoma.

All available data suggest that Nivolumab monotherapy has a consistent AE profile across tumor types. The safety profile is generally consistent across completed and ongoing clinical trials, with no maximum tolerated dose (MTD) reached at any monotherapy dose tested up to 10 mg/kg. There was no pattern in the incidence, severity, or causality of AEs to

Nivolumab dose level. The safety profile of Nivolumab in combination with Ipilimumab was consistent with the mechanisms of action of Nivolumab and Ipilimumab. The nature of the AEs was similar to that observed with either agent used as monotherapy; however, both frequency and severity of most AEs were increased with the combination. A dose of 3 mg/kg Nivolumab/3 mg/kg Ipilimumab exceeded the MTD, and both 1 mg/kg Nivolumab/3-mg/kg Ipilimumab and 3 mg/kg Nivolumab/1 mg/kg Ipilimumab were identified as the MTD. Across all studies conducted to date, drug-related AEs have included pulmonary toxicity, renal toxicity (including acute renal failure), endocrine abnormalities, GI toxicity, dermatologic toxicity (including rash), and hepatotoxicity. For Nivolumab monotherapy and combination therapy, the majority of these AEs have been managed successfully with supportive care and, in more severe cases, a combination of dose delay, permanent discontinuation, and/or use of corticosteroids or hormone replacement therapy (endocrinopathies) as instructed in the management guidelines provided in Appendix 3 of the IB.

In addition to BMS-sponsored ongoing studies, 22 studies sponsored by ONO Pharmaceuticals, Ltd. and conducted in Japan, Korea, and/or Taiwan are included in the reference safety information in Section 5.6 of the IB. Brief descriptions of these studies are provided in Appendix 4 of the IB. The studies are not under any US investigational new drug application (IND). Efficacy and safety information from ONO studies (ONO-4538-01, ONO-4538-04, ONO-4538-07, ONO-4538-12, ONO-4538-13, and ONO-4538-14) are provided in Section 5.4 and Section 5.5 of the IB.

All studies were conducted in accordance with Good Clinical Practice, as defined by the International Conference on Harmonisation and in accordance with the ethical principles underlying European Union Directive 2001/20/EC and the US Food and Drug Administration Code of Federal Regulations (CFR), Title 21, Part 50 (21CFR50).

1.2.5 Clinical Safety Summary

<u>BAT:</u>
The majority of adverse events (AEs) occurred during the initial phase of treatment and were largely consistent with known side effects of etoposide. Initial-phase side effects were mostly low grade (i.e. ≤ Grade 2) and included: nausea (N=10), fatigue (N=9), alopecia (N=9), edema (N=8), and neutropenia (N=3), as listed in the table below. Two patients had a grade 3 asymptomatic, subsegmental pulmonary embolism. Two subjects did not complete the initial treatment phase, one individual was taken off study after developing grade 2 priapism, and a second individual expired due to pneumonia/neutropenic sepsis. AEs occurring during the BAT monotherapy phase of the trial were rare and low grade. Only four subjects experienced an AE during this phase, and all but three AEs were grade 1. Grade 2 events included alopecia and an elevated creatinine in one subject and grade 2 nausea in a separate subject.

None of the 14 patients developed new pain, skeletal events or urinary obstruction due to prostate cancer. Although quality of life was not formally evaluated in the study, most subjects reported enhanced well-being and increased functional activity. Patients with intact sexual function prior to ADT had return of sexual function and libido on BAT.

Adverse events occurring in >15% of subjects and severe (grade ≥4) events.

Adverse Event	Grade 1-2 N (%)	Grade 3-4 N (%)	Any Grade N(%)
Anemia	3 (18.8)	0	3 (18.8)
Dysgeusia	3 (18.8)	0	3 (18.8)
Weight gain	3 (18.8)	0	3 (18.8)
Anorexia	4 (25)	0	4 (25)
Breast sensitivity	4 (25)	0	4 (25)
Neutropenia	3 (18.8)	1 (6.3)	4 (25)
Edema	8 (50)	0	8 (50)
Alopecia	9 (56.3)	0	9 (56.3)
Fatigue	9 (56.3)	0	9 (56.3)
Nausea	10 (62.5)	0	10 (62.5)
Pulmonary embolism	0	2 (12.5)	2 (12.5)
Death	0	1 (6.3)	1 (6.3)

Nivolumab:
The overall safety experience with Nivolumab as a monotherapy, is based on experience in approximately 11,000 subjects treated to date. For monotherapy, the safety profile is similar across tumor types. The only exception is pulmonary inflammation adverse events (AEs), which may be numerically greater in subjects with NSCLC, because in some cases, it can be difficult to distinguish between Nivolumab-related and unrelated causes of pulmonary symptoms and radiographic changes. There is no pattern in the incidence, severity, or causality of AEs to Nivolumab dose level. A detailed list of AEs for patients treated with Nivolumab as monotherapy on clinical trials can be found in Table 5.6.1-1 in the IB.

In several ongoing clinical trials, the safety of Nivolumab in combination with other therapeutics such as Ipilimumab, cytotoxic chemotherapy, anti-angiogenics, and targeted therapies is being explored. Most studies are ongoing and, as such, the safety profile of Nivolumab combinations continues to evolve.

1.3 Rationale

1.3.1 Rationale for Conducting the Study

<u>BAT and DNA Repair:</u>
We have discovered a provocative association between germline and/or somatic DNA-repair mutations and response to BAT. Based on prior studies, the incidence of germline mutations in DNA-repair genes in mCRPC patients is 8-12%, with somatic DNA-repair mutations approaching 20-25%. As mentioned, we have recently identified a correlation between long term ("exceptional") responders to BAT and the presence of mutations in homologous recombination DNA repair genes. Our index case was an enzalutamide-pretreated patient with mCRPC who achieved a durable (> 24 mo) complete radiographic and serologic response following treatment with BAT (Figure 6).(38) Tumor DNA sequencing found inactivating mutations in both BRCA2 (with LOH) and ATM (mono-allelic), which were then confirmed on germline DNA testing (i.e. these were both inherited mutations). We have since systematically sequenced tumor/germline DNA from patients receiving BAT and, remarkably, have detected DNA repair mutations in the majority of responders (Table 1). Overall, our preliminary unpublished findings suggest an 80% response rate to BAT (4/5) in men with DNA repair mutations, implying a provocative association between DNA repair-deficient prostate cancer and BAT responsiveness. This is a rational inference based on the fact that BAT may induce dsDNA breaks that may go unrepaired in the setting of homologous recombination defects.

Immune Checkpoint Blockade and DNA Repair:

Two randomized phase III trials involving single-agent checkpoint (CTLA-4) inhibitors in mCRPC patients did not meet their primary endpoints.(39,40) However, a small subset (~20%) of mCRPC patients did derive clinical benefit, although the molecular characteristics of these patients are unknown. In pilot studies conducted at Johns Hopkins, we have observed significant responses to checkpoint inhibition in several heavily pretreated mCRPC patients with DNA repair defects (Figure 7). For instance, two patients (#1: MSH6-mutated; #2: BRCA2-mutated) achieved a PSA_{50} response following combined PD-1/CTLA-4 checkpoint inhibition using Ipilimumab plus Nivolumab. Patient #1 also had a partial radiographic RECIST response and remains progression-free for > 6 months. Patient #2 had over 20 sites of symptomatic bone metastases at that start of therapy. Following combination checkpoint inhibition, his pain disappeared (pain score: 6/10 → 0/10) and his clinical response is ongoing after 9 months. A third mCRPC patient (#3: ATM-mutated) achieved a partial radiographic RECIST response without a PSA response. A fourth patient (#4: POLH-mutated) also had a RECIST response without a PSA response. Moreover, those patients with a DNA repair mutation had significantly improved radiographic-PFS (HR 0.28, p=0.027) and PSA-PFS (HR 0.14,P=0.001) compared to DNA repair-proficient patients. Similar to the efficacy of checkpoint inhibitors in patients with MSI-High (mismatch repair-deficient) colon cancer, we speculate that a benefit of immunotherapy may be observed in mCRPC patients with germline and/or somatic mutations in DNA repair genes, including homologous recombination genes (i.e. not just MMR genes), thus potentially expanding the pool of mCRPC patients that may be susceptible to immune checkpoint blockade.

BAT/Immunotherapy Combination:

Even more notably, we have observed intriguing tumor responses in two mCRPC patients with intact DNA repair processes (i.e. no repair gene mutations) that were treated sequentially (on two different trials) with BAT followed by PD-1 inhibition, who achieved dramatic PSA/RECIST responses to PD-1 blockade (Figure 8). Patient #1 initially achieved a PSA_{50} response to BAT, which lasted 11 months, before developing subsequent progression. Upon starting checkpoint inhibition following BAT, his PSA level dropped from 56 ng/mL to 4 ng/mL in the first 12 weeks, accompanied by a dramatic soft tissue response (Figure 8A). Patient #2 also initially achieved a PSA_{50} response to BAT lasting 6 months, but eventually progressed. Following subsequent treatment with Pembrolizumab, his PSA dropped from 692 ng/mL to 3 ng/mL, with a partial radiographic response after only 3 months of anti-PD-1 therapy (Figure 8B). Importantly, neither patient had a DNA repair mutation although both benefited dramatically from PD-1 inhibition following BAT. Based on these preliminary data, we believe that further study of BAT with a checkpoint inhibitor is warranted.

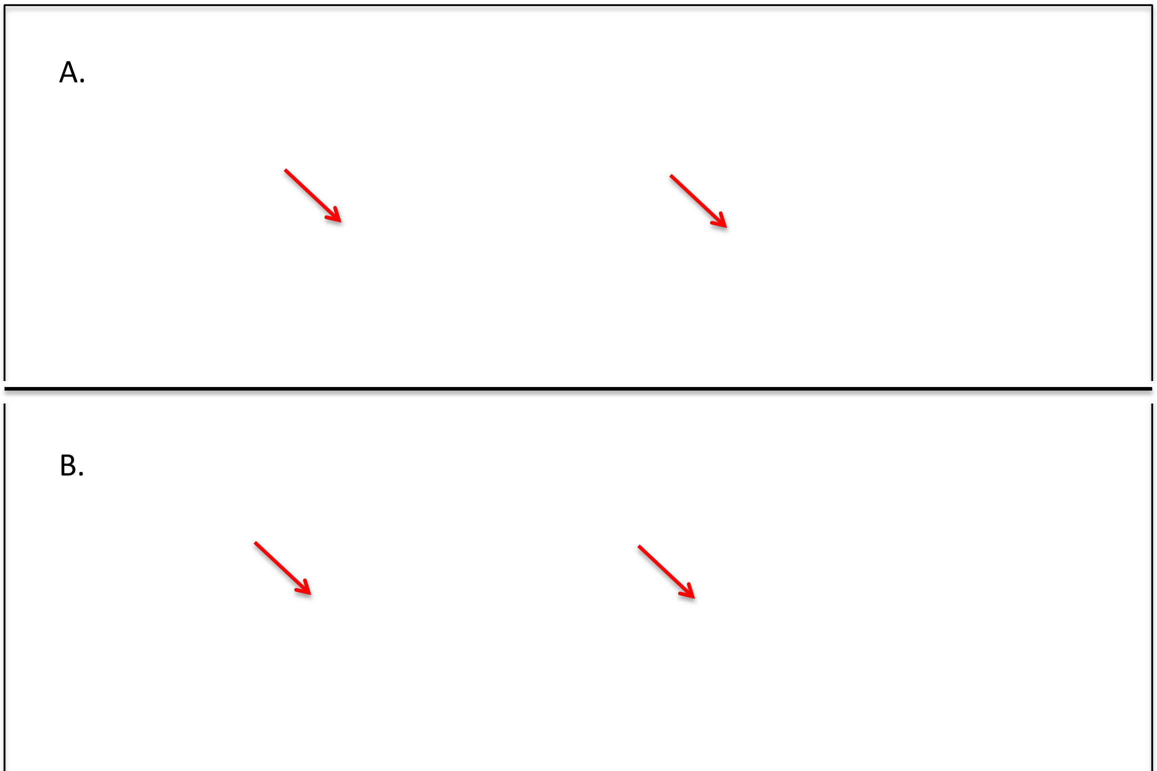

Figure 8. CT Imaging of Post-BAT mCRPC Patients Before and During Treatment With Checkpoint Inhibition. *Red arrows indicate target lesion.*

In this trial, we will adopt a timed-sequential approach, whereby patients will first receive BAT and followed 12 weeks later by the addition of Nivolumab. We hypothesize that (1) BAT-induced dsDNA breaks/rearrangements will result in neoantigen formation and increased sensitivity to Nivolumab (primary hypothesis); that (2) mCRPC patients with germline/somatic mutations in DNA repair genes will derive the greatest clinical benefit from both BAT and Nivolumab; and (3) that the combination of BAT with Nivolumab will be safe and well-tolerated in mCRPC patients resulting in minimal grade 3/4 toxicities.

1.3.2 Rationale for Dosage Selection

Nivolumab will be administered at a flat dose of 480mg IV given every 4 weeks until progression. Nivolumab has been extensively studied in humans at doses ranging from 1-10mg/kg every 2 weeks to 0.3-10mg/kg every 3 weeks. Using these doses, the clinical pharmacology of Nivolumab is well-established. Based on the exposure-response relationships for efficacy and safety, the benefit-risk profile of Nivolumab 480mg given every 4 weeks is predicted to be similar to the 3mg/kg dosing across multiple tumor types *(Zhao X et al. A Model-Based Exposure–Response (E–R) Assessment of a Nivolumab (NIVO) 4-Weekly (Q4W) Dosing Schedule Across Multiple Tumor Types. AACR 2017: CT101.)*

Testosterone cypionate will by administered at a dose of 400mg IM every 28 days (4 weeks). This dose of testosterone has been shown to be both safe and efficacious in mCRPC patients.

2. OBJECTIVES

2.1 Primary Objective

The primary objective is to estimate the PSA response rate (PSA_{50}) in patients with metastatic castration-resistant prostate cancer (mCRPC). This will serve as an initial exploration of this drug-drug combination's activity in this disease state.

2.2 Secondary Objectives

- Safety/Tolerability, defined as incidence of CTCAE grade ≥ 3 toxicities experienced by patients on the trial.
- PSA progression-free survival, defined as the time from initiation on BAT until PSA increase of 25%, confirmed with another measurement at least 4 weeks later (PCWG3).
- Progression-free survival, defined as the time to radiographic or clinical progression or death, whichever comes first. Based on RECIST version 1.1 and PCWG3 definitions including: 1. Progression of soft tissue lesions according to RECIST 1.1; 2. Progression of bony lesions detected by bone scan according to PCWG3 criteria; 3. Radiographically-confirmed spinal cord compression or pathological fracture due to malignant progression, or other clinical event deemed to be cancer-related.
- Objective response rate, defined as the proportion of patients achieving a complete/partial response in target lesions (RECIST 1.1).
- Durable progression-free survival (Durable-PFS), defined as the proportion of patients without clinical or radiographic progression (as defined by PCWG3) or death at 24 weeks from the start of treatment.
- Overall survival will be defined as the time from study enrollment to death or up to 2 years after the last dose of study treatment.

2.3 Correlative/Exploratory/Tertiary Objectives

- To estimate the percentage of patients with somatic (tumor) or germline mutations in homologous repair (HR) and mismatch DNA repair mutations.
- To measure changes in gamma-H2AX, RAD51, 53BP1 (DNA damage markers) following treatment with BAT and at progression on BAT/Nivolumab.
- To measure changes in CD4, CD8, FOXA3, Ki-67, PD-1, PD-L1 tumor levels with BAT alone and at progression following BAT/Nivolumab.
- To investigate any association between PD-1 and PD-L1 expression in tumor specimens and PSA_{50} response rates.
- To explore the relationship between mutation-associated neoantigens (MANAs) and tumor-associated neoantigens (TAAs) and clinical outcomes.
- To characterize the effect of BAT on production of inflammatory chemokines and cytokines.

3. PATIENT SELECTION

3.1 Target Population

The target population is men with metastatic castration-resistant prostate cancer with prior treatment of at least ONE novel AR-targeted therapy such as abiraterone acetate or enzalutamide. One prior chemotherapy agent for mCRPC will be allowed but is not required.

3.2 Expected Enrollment

A total of 44 patients will be included in this study. The first patients are expected to be enrolled in Q3/Q4 2018. Accrual is expected to be completed in 18 months once the protocol has been approved by the IRB at each participating institution.

3.3 Inclusion Criteria

To be included in this study, patients should meet all of the following criteria:

- Willing and able to provide signed informed consent.
- Males aged 18 years of age and above.
- Histological or cytologic proof of adenocarcinoma of the prostate.
- Known castration-resistant disease, defined according to PCWG3 criteria as:
    - Castrate serum testosterone level ≤ 50 ng/dL (≤ 1.7 nmol/L).
    - Subjects who have failed initial hormonal therapy, either by orchiectomy or by using a GnRH agonist in combination with an anti-androgen, must first progress through anti-androgen withdrawal prior to being eligible. The minimum timeframe to document failure of anti-androgen withdrawal will be 4 weeks.
    - Serum PSA progression defined as 2 consecutive increases in PSA over a previous reference value within 6 months of the first study treatment, each measurement at least 1 week apart.

      Or
    - Documented bone lesions by the appearance of ≥ 2 new lesions by bone scintigraphy or dimensionally-measureable soft tissue metastatic lesion assessed by CT or MRI.
- Absolute PSA ≥ 2.0 ng/mL at screening.
- Must have PSA and/or radiographic progression on AT LEAST ONE novel AR-targeted therapy (abiraterone acetate, enzalutamide). One prior chemotherapy agent for mCRPC will be allowed.
- Prior treatment with abiraterone, enzalutamide, bicalutamide, and/or ketoconazole is allowed. There is no limit on the maximum number or types of prior hormonal therapies received.
- Must be maintained on a GnRH analogue or have undergone orchiectomy.
- Radiographic evidence of metastatic disease by CT scan and/or bone scan, performed within the prior 4 weeks of the screening visit.

- Must have a soft tissue lesion available for biopsy collection to perform tumor tissue analysis.
- Karnofsky Performance Status (KPS): ≥ 70% within 28 days before start of study treatment (ECOG ≤ 2) (See Appendix B).
- Participants must have normal organ and bone marrow function measured within 28 days prior to administration of study treatment as defined below:
    - Hemoglobin ≥ 9.0 g/dL with no blood transfusion in the past 28 days
    - Absolute neutrophil count (ANC) ≥ 1.0 x 10^9/L
    - Platelet count ≥ 100 x 10^9/L
    - Total bilirubin within institutional upper limit of normal (ULN) (In patients with Gilbert's syndrome, total bilirubin < 1.5x institutional ULN will be acceptable)
    - Aspartate aminotransferase (AST) (Serum Glutamic Oxaloacetic Transaminase (SGOT)) / Alanine aminotransferase (ALT) (Serum Glutamic Pyruvate Transaminase (SGPT)) within institutional upper limit of normal
    - Participants must have Creatinine Clearance estimated using the Modified Cockcroft-Gault equation of ≥ 40 mL/min:

      $$\text{Estimated Creatinine Clearance} = \frac{(140\text{-age [years]}) \times \text{weight (kg)}}{\text{serum creatinine (mg/dL)} \times 72}$$

- Participants must have a life expectancy ≥ 6 months.
- Male participants and their partners, who are sexually active and of childbearing potential, must agree to the use of two highly effective forms of contraception in combination [see appendix E for acceptable methods], throughout the period of taking study treatment and for 7 months after the last dose of Nivolumab to prevent pregnancy in a partner.
- No evidence (within 5 years) of prior malignancies (except successfully treated basal cell or squamous cell carcinoma of the skin).

3.4 Exclusion Criteria

Patients that meet any of the criteria listed below will not be eligible for study entry:

- Has received external-beam radiotherapy within the last 2 weeks prior to start of study treatment.
- Prior oral anti-androgen (e.g. bicalutamide, nilutamide, enzalutamide, apalutamide), or androgen synthesis inhibitor (e.g. abiraterone, orteronel) within the past 2 weeks is not permitted. 5-alpha reductase inhibitor therapy (e.g. finasteride, dutasteride) is allowed, as long as subject has been stable on medication for past 6 months.
- Prior treatment with chemotherapy for the treatment of metastatic hormone-sensitive prostate cancer is allowed if the last dose of chemotherapy was ≥ 6 months prior to enrollment. In addition, one prior chemotherapy agent for mCRPC will be allowed after a minimum wash-out period of 4 weeks prior to enrollment.

- Patients who have received prior treatment with bipolar androgen therapy (e.g. high-dose testosterone, BAT) for castrate-resistant prostate cancer (CRPC); permitted if used for hormone-sensitive prostate cancer (HSPC).
- Pain due to metastatic prostate cancer requiring opioid therapy.
- Patients with an intact prostate AND urinary obstructive symptoms are excluded (which includes patients with urinary symptoms from benign prostatic hyperplasia (BPH), except where the urinary tract obstruction has been medically or surgically resolved.
- Patients receiving anticoagulation therapy with Coumadin are not eligible for study. [Patients on non-coumadin anticoagulants (Lovenox, Xarelto, etc.) are eligible for study. Patients on Coumadin who can be transitioned to Lovenox or Xarelto prior to starting study treatments will be eligible] .
- Patients with prior history of an arteriovenous thromboembolic event that occurred within the last 12 months are excluded.
- Patients allergic to sesame seed oil or cottonseed oil are excluded.
- Involvement in the planning and/or conduct of the study (applies to both BMS staff and/or staff at the study site).
- Participation in another clinical study with an investigational product during the last 4 weeks/28 days.
- Patients should be excluded if they have had prior systemic treatment with an anti-PD-1, anti-PD-L1, anti-PD-L2, anti-CTLA-4 antibody, or any other antibody or drug specifically targeting T cell co-stimulation or immune checkpoint pathways (e.g. immune checkpoint antagonists).
- Evidence of disease in sites or extent that, in the opinion of the investigator, would put the patient at risk from therapy with testosterone (e.g. femoral metastases with concern over fracture risk, severe and extensive spinal metastases with concern over spinal cord compression, extensive liver metastases).
- Concurrent use of other anticancer agents or treatments, with the following exceptions:
    - Ongoing treatment with LHRH agonists or antagonists, denosumab (Prolia) or bisphosphonate (e.g. zoledronic acid) is allowed. Ongoing treatment should be kept at a stable schedule; however, if medically required, a change of dose, compound, or both is allowed.
- Any treatment modalities involving major surgery within 4 weeks prior to the start of study treatment.
- Symptomatic nodal disease, i.e. scrotal, penile or leg edema (CTCAE ≥ Grade 3).
- Patients are excluded if they have active, known brain metastases or leptomeningeal metastases. Subjects with brain metastases are eligible if metastases have been treated and there is no magnetic resonance imaging (MRI) evidence of progression for ≥ 4 weeks after treatment is complete and within 28 days prior to the first dose of testosterone administration. There must also be no requirement for

immunosuppressive doses of systemic corticosteroids (> 10 mg/day prednisone equivalents) for ≥ 2 weeks prior to study drug administration.

- Patients should be excluded if they have an active, known or suspected autoimmune disease (e.g. inflammatory bowel disease, rheumatoid arthritis, autoimmune hepatitis, lupus, celiac disease). Subjects are permitted to enroll if they have vitiligo, type I diabetes mellitus, residual hypothyroidism due to autoimmune condition only requiring hormone replacement, psoriasis not requiring systemic treatment, or conditions not expected to recur in the absence of an external trigger.
- Patients should be excluded if they have a condition requiring systemic treatment with either corticosteroids (> 10 mg/day prednisone equivalents) or other immunosuppressive medications within 14 days of study drug administration. Inhaled or topical steroids and adrenal replacement doses > 10 mg/day prednisone equivalents are permitted in the absence of active autoimmune disease.
- Permitted therapies include topical, ocular, intra-articular, intranasal, and inhalational corticosteroids (with minimal systemic absorption). Physiologic replacement doses of systemic corticosteroids are permitted, even if > 10 mg/day prednisone equivalents. A brief course of corticosteroids for prophylaxis (e.g. contrast dye allergy) or for treatment of non-autoimmune conditions (e.g. delayed-type hypersensitivity reaction caused by contact allergen) is permitted.
- As there is potential for hepatic toxicity with Nivolumab, drugs with a predisposition to hepatotoxicity should be used with caution in patients treated with Nivolumab-containing regimen.
- Patients should be excluded if they have a positive test for hepatitis B virus surface antigen (HBV sAg) or hepatitis C virus ribonucleic acid (HCV antibody) indicating acute or chronic infection.
- Patients should be excluded if they have known history of testing positive for human immunodeficiency virus (HIV) or known acquired immunodeficiency syndrome (AIDS).
- History of allergy to study drug components.
- History of severe hypersensitivity reaction to any monoclonal antibody.
- Other primary tumor (other than CRPC) including hematological malignancy present within the last 5 years (except non-melanoma skin cancer or low-grade superficial bladder cancer).
- Has imminent or established spinal cord compression based on clinical findings and/or MRI.
- Any other serious illness or medical condition that would, in the opinion of the investigator, make this protocol unreasonably hazardous, including, but not limited to:
    - Any uncontrolled major infection.
    - Cardiac failure NYHA (New York Heart Association) III or IV.
    - Crohn’s disease or ulcerative colitis.
    - Bone marrow dysplasia.
    - Known allergy to any of the compounds under investigation.

  - Unmanageable fecal incontinence.
- Persistent toxicities (CTCAE > Grade 2) caused by previous cancer therapy, excluding alopecia.
- Poor medical risk due to a serious, uncontrolled medical disorder, non-malignant systemic disease, or active, uncontrolled infection. Examples include, but are not limited to, uncontrolled ventricular arrhythmia, recent (within 6 months) myocardial infarction, uncontrolled major seizure disorder, extensive interstitial bilateral lung disease, or any psychiatric disorder that prohibits obtaining informed consent.

4. PATIENT REGISTRATION AND ENROLLMENT PLAN

4.1 Registration Procedure

After eligibility screening and confirmation that a patient is eligible, patients who are selected to participate will be registered with the Lead Center, Johns Hopkins University, with their local study site/institution, and if applicable, in the online centralized PCCTC database. A record of patients who fail to meet entry criteria (i.e. screen failures) will be maintained. Patient registration must be complete before beginning any treatment or study activities. A complete, signed study consent is required for registration.

4.1.1 Registration at Johns Hopkins University

Confirm eligibility as defined in ***Section 3. Patient Selection***.

Obtain informed consent, by following procedures in ***Section 12.3 Written Informed Consent***.

Patient will be entered into the CRMS system and enrolled in the trial.

4.1.2 Multicenter/Participating Site Registration

Central registration for this study will take place at Johns Hopkins University.

Patient registration at each study site/institution will be conducted according to the institution's established policies. Before registration, patients will be asked to sign and date an Institutional Review Board (IRB)-approved consent form. Patients must be registered with their local site/institution and also with the Lead Site before beginning any treatment or study activities.

5. TREATMENT/INTERVENTION PLAN

The following assessments and procedures will occur during the study. A schedule of assessments is provided in Table 2.

Table 2. *Study Calendar*

		**Study Period**								
	**Screening**	**On Treatment Assessments (Every 28 days) BAT Alone**				**On Treatment Assessments (Every 28 days) Nivolumab + BAT**		**End of Treatment Visit**	**Off study follow-up**	**Survival follow-up**
	Day -28 to -1	C1D1 (±5 d)	JHU only C1D4 (±5 d)	C2D1 (±5 d)	C3D1 (±5 d)	C4D1 (±5 d)[c]	CnD1 (±5 d)	28 days after last dose (±5 d)[c]	Every 28 days [e] / 100 days after last dose (±5 d)	Up to 1 year after last dose or AE resolution date[e] (Q6mo.)
Informed Consent	X[a]									
Demographics	X									
Complete Medical History	X									
EKG	X[k]									
Testosterone cypionate		X		X	X	X	X			
Nivolumab						X	X			
Focused Medical History		X		X	X	X	X	X		
Physical Exam	X[l]	X[l]		X[l]	X[l]	X[l]	X[l]	X[l]		
Vital Signs (P, BP, RR, T)	X[l]	X[l]		X[l]	X[l]	X[l]	X[l]	X[l]		
Height	X[l]									
Weight	X[l]	X[l]		X[l]	X[l]	X[l]	X[l]	X[l]		
ECOG Performance Status (ECOG)	X			X	X	X	X	X		
Toxicity Assessment	X			X	X	X	X	X		
Concomitant Medications	X	X		X	X	X	X	X		
CBC w/ Diff	X	X[g]		X	X	X	X	X		
CMP	X	X[g]		X	X	X	X	X		
PSA	X	X[g]		X	X	X	X	X		

Serum Testosterone	X	X[g]	X	X	X	X	X	X		
Serum Amylase/Lipase/ CK; TSH w/ Reflex T3, T4	X					X	X	X		
Hepatitis B/C; PTT, PT/INR; Urinalysis	X									
Radiologic Tests	X[b]					X[bc]	X[b]	X[bc]		
Core Tumor Biopsy	X[f]					X[f]		X[f]		
Rectal Swab		X[i]				X[i]	X[i]			
Germline DNA Testing		X[h]								
Plasma for MANAs/TAAs		X[d]				X[d]		X[d]		
Plasma for Chemokines		X[j]	X[j]	X[j]			X[j]			
Adverse Events				X	X	X	X	X	X[e]	
Survival Contact										X[e]

Abbreviations: CBC, complete blood count; CT, computerized tomography; MRI, magnetic resonance imaging; PSA, prostate-specific antigen; CK, creatine kinase; TSH, thyroid stimulating hormone

[a] Informed consent should be obtained within 4 weeks (28 days) of study start date (C1D1).

[b] Radiographic evaluations (CT C/A/P) and NM Bone Scan every 3 months while enrolled in the study) are needed within 4 weeks of the screening visit; if previously performed within the last 4 weeks they do not need to be repeated. CT C/A/P and NM Bone Scan are to be performed at the End of Treatment (EOT) visit if they have not been performed within the prior month.

[c] For visits C4D1 and EOT only, the window for radiographic evaluations will be extended to 2 weeks before the visit occurs to account for biopsy scheduling availability.

[d] Plasma for MANAs/TAAs assay will be drawn at C1D1, C4D1, and at progression (EOT) for JHU patients ONLY.

[e] Subjects will be followed after withdrawal of BAT +/- Nivolumab therapy due to toxicity, monthly until toxicities resolve to grade ≤ 1. Otherwise, subjects will be followed at 100 days after last dose for AE reassessment. Survival follow-up will continue for up to 1 year through telephone contact every 6 months, to begin after the 100-day follow-up contact or from the AE resolution date if it exceeds the 100 day follow-up timepoint.

[f] MANDATORY core tumor biopsy performed during the Screening period (prior to C1D1) (Day -28 to -1) and on C4D1 (+/- 7 days). An OPTIONAL tumor biopsy will be performed at the End of Treatment visit (+/- 7 days).

[g] Labs do not need to be repeated if done within 7 days of screening.

[h] Germline DNA testing per standard of care practice is optional. To be submitted after obtaining informed consent and prior to C2D1.

[i] Rectal Swabs to be performed before treatment with testosterone (C1D1), prior to starting Nivolumab (C4D1), and after 3 cycles of Nivolumab (C7D1) for JHU patients ONLY.

[j] Plasma sample for Chemokines analysis (optional) will be obtained at C1D1 prior to the first injection of BAT, 3 hours after the first injection of BAT, C1D4, C2D1, and C7D1 for JHU patients ONLY.

[k] EKG's must be collected within 14 days of receiving the first study treatment.

[l] Physical exam (including vital signs/height/weight) may be missed if not clinically indicated or in the event of a telemedicine visit. In order to minimize the need for research-only in-person visits, telemedicine visits may be substituted for in-person clinical trial visits or portions of clinical trial visits where determined to be appropriate and where determined by the investigator not to increase the participants risks. Prior to initiating telemedicine for study visits the study team will explain to the participant, what a telemedicine visit entails and confirm that the study participant is in agreement and able to proceed with this method. Telemedicine acknowledgement will be obtained in accordance with the Guidance for Use of Telemedicine in Research. In the event telemedicine is not deemed feasible, the study visit will proceed as an in-person visit. Telemedicine visits will be conducted using HIPAA compliant method approved by the Health System and within licensing restrictions.

5.1 Screening Assessment (Day -28 to Day -1)

Before initiating any screening activities, the scope of the study should be explained to each patient. Patients should be advised of any known risks inherent in the planned procedures, any alternative treatment options, their right to withdraw from the study at any time for any reason, and their right to privacy. After this explanation, patients should be asked to sign and date an IRB-approved informed consent that meets the requirements of the Code of Federal Regulations (Federal Register Vol. 46, No. 17, January 27, 1981, part 50).

The screening visit will determine patient eligibility according to the inclusion and exclusion criteria (Sections 3.3 Inclusion Criteria & 3.4 Exclusion Criteria). The following assessments will be performed at this visit:

- Obtain informed consent and research authorization.
- Record demographics (including age) and medical history (including prior treatment for prostate carcinoma).
- Conduct physical exam (including vital signs, height/weight). *The physical exam may be missed if screening visit is taking place over telemedicine.*
- Obtain histologic and radiologic confirmation of disease. If radiographic studies have not been performed in prior 4 weeks of the screening visit, they must be obtained as part of screening.
- Obtain history regarding prior treatment history for prostate cancer (including history of ADT, history of radiation therapy, or other local therapy).
- Perform laboratory tests (Complete blood count w/ Diff, PSA, Comprehensive metabolic panel, urinalysis, PT/INR, PTT, serum testosterone, TSH with reflexive T3, T4 testing, serum Creatine kinase (CK), serum amylase/lipase, and Hepatitis B/C panel).
- Assess performance status (ECOG). (Appendix B).
- Perform 12-lead EKG. ECGs are required within 14 days prior to starting study treatment and when clinically indicated.
    - Twelve-lead ECGs will be obtained after the patient has been rested in a supine position for at least 5 minutes in each case. The Investigator or designated physician will review the paper copies of each of the timed 12-lead ECGs on each of the study days when they are collected.
    - ECGs will be recorded at 25 mm/sec. All ECGs should be assessed by the Investigator as to whether they are clinically significantly abnormal or not clinically significantly abnormal. If there is a clinically significant abnormal finding, the Investigator will record it as an AE on the eCRF. The original ECG traces must be stored in the patient medical record as source data.
- Determine suitability for Testosterone + Nivolumab.

- Obtain image-guided biopsy of a site of disease (i.e. soft tissue, lymph node) – further described in section 5.1.1.
- Discuss concurrent medications.
- Provide instructions for obtaining a CLIA-certified laboratory (i.e. COLOR genomics) kit for germline DNA testing per standard of care practice (Optional).

Relevant information should be documented. The institutional registration should be finalized, and appropriate documents (i.e. signed informed consent and supporting source documentation for eligibility questions) emailed to the Lead site.

Information for patients who do not meet the eligibility criteria to participate in this study (i.e. screening failures) should be captured in the CRMS database at the screening assessment.

5.1.1 Surgical Procedures

A core needle biopsy of a site of disease (soft tissue, lymph node) will be performed during the Screening period (prior to C1D1). Patients will be referred to their respective biopsy service for an image-guided core biopsy. At least 4 standard core biopsies should be attempted to be obtained from the site (soft tissue, lymph node). *Fine needle aspiration is unlikely to yield adequate tumor tissue for molecular analysis, and should only be attempted when core biopsy is not feasible.* The unique subject number given by the Lead site during registration should be used to identify the biopsy samples to protect the subject's identity.

For each biopsy timepoint, ≥ 4 fresh cores will be collected from the **same** site of disease chosen during the Screening period (prior to C1D1); ≥ 2 fresh cores will be collected for immediate flash-freezing in liquid nitrogen and ≥ 2 cores will be formalin-fixed and paraffin-embedded. For soft-tissue biopsies, 18-gauge core biopsy needles are recommended.

To minimize tissue autolysis, a dedicated tissue technician should be appointed and called/paged to the biopsy room to initiate processing steps including FFPE preparation, which should be done within 30 minutes of biopsy collection, and the flash-freezing of the other designated cores with liquid nitrogen. FFPE specimens should be processed according to standard institutional FFPE procedures. All samples will be sent to the De Marzo Laboratory for analysis.

Note. If site(s) do not have the capability to perform flash-freezing of tumor tissue cores with liquid nitrogen, all available tumor tissue core specimens should be prepared and processed according to standard institutional FFPE procedures.

5.1.1.1 *Potential Biopsy Complications*

Potential complications of a research biopsy include: infection, bleeding, and pain at the biopsy site.

5.2 Testosterone Lead-in Period (Day 1 of each 28 day cycle +/- 5 days, (C1D1, C2D1, C3D1))

Patients will be seen on Day 1 of each cycle of testosterone (consisting of 28 days, +/- 5 days). The following assessments will be performed at each visit:

- Conduct physical exam (including vital signs, weight). *The physical exam may be missed if visit is taking place over telemedicine.*
- Obtain any medical history changes from prior assessment.
- Assess performance status (ECOG). (Appendix B).
- Review concurrent medications.
- Confirm germline DNA sample submitted per standard of care practice, if applicable (To be submitted prior to C2D1).
- Obtain Rectal swab for microbiome analysis for JHU patients ONLY.

5.2.1 Clinical and Laboratory Assessments

On Day 1 of each cycle, patients will have a non-fasting blood draw for the following values: (Repeat labs are not needed on C1D1 if within 7 days of screening values.)

- CBC w/ Diff
- Comprehensive metabolic panel
- PSA
- Serum Testosterone
- MANAs/TAAs assay blood draw (100mL; C1D1 only) for JHU patients ONLY
- Chemokines plasma blood draw (10mL; C1D1 predose, 3 hours after first testosterone injection, C1D4, C2D1) for JHU patients ONLY (Optional).

Laboratory Safety Assessments:

Full hematology assessments for safety (hemoglobin, red blood cells [RBC], platelets, mean cell volume [MCV], mean cell hemoglobin concentration [MCHC], mean cell hemoglobin [MCH], white blood cells [WBC], absolute differential white cell count (neutrophils, lymphocytes, monocytes, eosinophils and basophils) and absolute neutrophil count or segmented neutrophil count and Band forms should be performed at each visit and when clinically indicated. If absolute differentials not available, will check % differentials. Coagulation [activated partial thromboplastin time (APTT) and international normalized ratio (INR)] will be performed at baseline and if clinically indicated.

Biochemistry assessments for safety (sodium, potassium, calcium, glucose, creatinine, total bilirubin, alkaline phosphatase [ALP], aspartate transaminase [AST], alanine transaminase [ALT], urea or blood urea nitrogen [BUN], total protein, albumin.

Urinalysis should be performed at baseline and then only if clinically indicated. Microscopic analysis should be performed by the hospital's local laboratory if required.

These tests will be performed by the hospital's local laboratory. Additional analyses may be performed if clinically indicated.

Any clinically significant abnormal laboratory values should be repeated as clinically indicated and recorded on the eCRF.

In case a subject shows an AST **or** ALT ≥ 3xULN **or** total bilirubin ≥ 2xULN, please refer to Appendix F *"Actions required in cases of combined increase of Aminotransferase and Total Bilirubin – Hy's Law"*, for further instructions.

5.2.3 *Safety Assessments*

Adverse events (AEs) will be monitored at each scheduled visit and throughout the study. Toxicity will be assessed using the most recent National Cancer Institute (NCI) guidance: the most recent version of Common Terminology Criteria for Adverse Events (CTCAE).

5.3 Testosterone/Nivolumab Combination Period (Day 1 of each 28 day cycle +/- 5 days, (C4D1, C5D1, CnD1, continuous))

Patients will be seen on D1 of each cycle of Testosterone/Nivolumab (consisting of 28 days, +/- 5 days). The following assessments will be performed at each visit:

- Conduct physical exam (including vital signs, weight). *The physical exam may be missed if visit is taking place over telemedicine.*
- Obtain any medical history changes from prior assessment.
- Assess performance status (ECOG). (Appendix B).
- Review concurrent medications.

5.3.1 *Clinical and Laboratory Assessments*

On Day 1 of each cycle (beginning C4D1), patients will have a non-fasting blood draw for the following values:

- CBC w/ Diff
- Comprehensive metabolic panel
- PSA
- Serum Testosterone
- TSH w/ reflexive T3, T4
- Serum Amylase/Lipase
- Serum Creatine kinase (CK)
- MANAs/TAAs assay blood draw (C4D1, End of Treatment) for JHU patients ONLY
- Rectal swab will be obtained prior to C4D1 and C7D1 for JHU patients ONLY
- Chemokines plasma blood draw (10mL; C7D1) for JHU patients ONLY (Optional)

Every 3 cycles, patients will have radiographic studies beginning C4D1:

- CT Chest, Abdomen, and Pelvis with contrast.
- NM Bone scan.

Note: For visits C4D1 and EOT only, the window for obtaining radiographic evaluations will be extended to 2 weeks before the visit occurs to account for biopsy scheduling availability.

5.3.2 Surgical Procedures
A core needle biopsy will be performed on C4D1 (+/- 7 days) at the **same** site of disease previously biopsied during the Screening period. This biopsy is considered mandatory. However, patients can remain on study if biopsy is refused. Further description of this biopsy can be found in section 5.1.1.

5.4 End of Treatment/Treatment Discontinuation Visit (28 days after last dose +/- 5 days)

- Conduct physical exam (including vital signs, weight). *The physical exam may be missed if visit is taking place over telemedicine.*
- Assess performance status (ECOG). (Appendix B).
- Review concurrent medications.
- Assess AEs.
- If patient is discontinuing participation in study, perform radiographic tests: CT C/A/P and NM Bone Scan (if not performed within the prior month).
- The following non-fasting blood draw will include:
    - CBC w/ Diff
    - Comprehensive metabolic panel
    - PSA
    - Serum Testosterone
    - TSH w/ reflexive T3, T4
    - Serum Amylase/Lipase
    - Serum Creatine kinase (CK)
    - MANAs/TAAs assay blood draw (if not done within the prior 90 days) for JHU patients ONLY

5.4.1 Surgical Procedures
A core needle biopsy will be performed at progression (EOT) (+/- 7 days) at the **same** site of disease previously biopsied during the Screening period. This biopsy is considered optional. Further description of this biopsy can be found in section 5.1.1.

5.5 Follow-up (Every 28 days while active AEs/100 days after last dose & Survival status +/- 5 days)

Patients that received Nivolumab and discontinue due to progression, will be followed and contacted at 100 days after last dose.

Patients that have discontinued the study due to AEs will be followed every 28 days (monthly) beyond the end of treatment (EOT) visit. They will be followed until the AE has either resolved or stabilized (Grade ≤ 1). If the event resolves or stabilizes before they reach 100 days after last dose, they will be contacted at the 100 day timepoint. Reasons for premature withdrawal should be determined and noted.

To be performed at each contact will be:

- Reassess AEs.

Following completion of the 100 day follow-up OR the resolution/stabilization of the AE(s) responsible for premature withdrawal (if beyond 100 days after last dose), patients will be followed for overall survival every 6 months for 1 year.

To be performed at each contact will be:

- Reassess for survival.

5.6 Correlative/Special Studies

5.6.1. DNA Sequencing
FFPE biopsy tissue (collected during the Screening period) will be sent to Foundation Medicine for next generation sequencing, based upon their commercially available platform.

5.6.2. PD-1, PD-L1, FOXP3, CD4, CD8, γH2AX, RAD51, 53BP1 Protein Levels
A sample of biopsy (from each biopsy done on study) will be sent to the De Marzo Laboratory for analysis.

5.6.3. Whole Exome Sequencing
Flash frozen biopsy tissue collected from the Screening period/C4D1/ progression (EOT) biopsies will be sent to the De Marzo Laboratory for whole exome sequencing in collaboration with the Next Generation Sequencing Core Lab to be performed in concert with the MANAs/TAAs assays.

5.6.4. MANAs/TAAs Assay
Plasma will be obtained at C1D1, C4D1, and at progression (EOT). The MANAs/TAAs assay will be performed by the Immune Processing Core Lab (Appendix C).

5.6.6. Chemokines Analysis
Plasma will be obtained prior to the first BAT injection on C1D1, 3 hours and 3 days (C1D4) after the first testosterone injection, C2D1, and C7D1. Samples will be processed and stored in the Denmeade Laboratory at Johns Hopkins. Analysis will be performed by the Immune Processing Core Lab.

6. Study Drugs

6.1 Description of Treatments

The drugs to be tested in this clinical protocol are Testosterone cypionate and Nivolumab. Nivolumab will be supplied by Bristol-Myers Squibb, Co.

6.2 Administration, Supply, and Storage

Nivolumab will be dosed at 480mg IV over a 30 minute period every 4 weeks (+/- 5 days) beginning on C4D1. Testosterone cypionate will be dosed at 400mg IM every 4 weeks (+/- 5 days) beginning on C1D1. Treatment will be administered on an outpatient basis.

Nivolumab:

PRODUCT INFORMATION TABLE: Please also see Nivolumab Investigator Brochure.

**Table**	**Product Description**				
**Product Description and Dosage Form**	**Potency**	**Primary Packaging (Volume)**	**Secondary Packaging (Qty)/ Label Type**	**Appearance**	**Storage Conditions (per label)**
Nivolumab BMS-936558-01 Solution for Injection	100 mg (10 mg/mL)	10 mL vial	5 vials per carton/Open-label	Clear to opalescent colorless to pale yellow liquid. May contain particles.	2 to 8°C. Protect form light and freezing.

*Nivolumab may be labeled as BMS-936558-01 Solution for Injection

If stored in a glass front refrigerator, vials should be stored in the carton. Recommended safety measures for preparation and handling of Nivolumab include laboratory coats and gloves.

For additional details on prepared drug storage and use time of Nivolumab under room temperature/light and refrigeration, please refer to the BMS-936558 (Nivolumab) Investigator Brochure section for “Recommended Storage and Use Conditions”.

Testosterone cypionate

Testosterone cypionate injection is for intramuscular use only. It should not be given intravenously. Intramuscular injections should be given deep in the gluteal muscle.

Testosterone will be used in the commercially available formulation, it will be purchased by the participant’s site pharmacy; the cost will be covered by the study and will be provided to the patient at no charge.

Vials should be stored at controlled room temperature 20°C to 25°C (68°F to 77°F) [see USP]. Protect from light. Use carton to protect contents from light until used. Warming and rotating the vial between the palms of the hands will redissolve any crystals that may have formed during storage at low temperatures.

7 DOSE ADJUSTMENT AND DELAY, TREATMENT DISCONTINUATION, WITHDRAWAL, AND TERMINATION CRITERIA

7.1 Dosing and Dose Modifications

7.1.1 Dosing

Testosterone cypionate will be given at a dose of 400mg IM every 28 days (4 weeks) approximately (+/- 5 days) beginning on C1D1 until progression or end of study.

Nivolumab 480mg will administered intravenously over a 30 minute period every 4 weeks (+/- 5 days) beginning on C4D1 until progression or end of study.

7.1.2 Dose Modifications

There will be no dose modifications permitted. Dose reductions or dose escalations are not permitted.

7.2 Dose Delay and Treatment Discontinuation

Dose delay criteria apply for all drug-related adverse events (regardless of whether or not the event is attributed to Nivolumab, Testosterone, or both). All study drugs must be delayed until treatment can resume.

7.3 Dose Delay Criteria

Nivolumab and Testosterone administration should be delayed for the following:

- Any Grade ≥ 2 non-skin, drug-related adverse event, with the following exceptions:
  - Grade 2 drug-related fatigue or laboratory abnormalities do not require a treatment delay.
- Any Grade 3 skin, drug-related adverse event.
- Any Grade 3 drug-related laboratory abnormality, with the following exceptions for AST, ALT, or total bilirubin:
  - Delay dosing for drug-related AST, ALT, or total bilirubin grade ≥ 2 toxicity.
- Grade 3 amylase or lipase abnormalities that are not associated with symptoms or clinical manifestations of pancreatitis do not require a dose delay.
- Grade 3 lymphopenia does not require a dose delay.
- Any adverse event, laboratory abnormality, or intercurrent illness, which, in the judgment of the investigator, warrants delaying the dose of study medication.

Because of the potential for clinically meaningful Nivolumab-related AEs requiring early recognition and prompt intervention, management algorithms have been developed for suspected pulmonary toxicity, GI, hepatotoxicity, endocrinopathy, skin toxicity, neurological toxicity and renal toxicity. The recommendations are to follow the Nivolumab IB adverse event algorithm (Appendix A).

7.4 Criteria to Resume Treatment

Missed doses of Nivolumab and/or Testosterone should be administered when subject meets criteria to resume treatment. If a Testosterone and Nivolumab dose has been missed, the subject should wait until the next scheduled dosing date following the originally planned dosing schedule that is consistent with the study calendar.

Subjects may resume treatment with study drug when the drug-related AE(s) resolve to Grade ≤ 1 or baseline value, with the following exceptions:

- Subjects may resume treatment in the presence of Grade 2 fatigue.
- Subjects who have not experienced a Grade 3 drug-related skin AE may resume treatment in the presence of Grade 2 skin toxicity.
- For subjects with Grade 2 AST, ALT, or total bilirubin elevations, dosing may resume when laboratory values return to baseline and management with corticosteroids, if needed, is complete.
- Subjects with combined Grade 2 AST/ALT **<u>AND</u>** total bilirubin values meeting discontinuation parameters should have treatment permanently discontinued.
- Drug-related diarrhea, or colitis, must have resolved to baseline before treatment is resumed.
- Subjects with persistent Grade 1 pneumonitis after completion of a steroid taper over at

least 1 month may be eligible for retreatment if discussed with and approved by the investigator.

- Drug-related endocrinopathies adequately controlled with only physiologic hormone replacement may resume treatment.

If treatment is delayed > 10 weeks, the subject must be permanently discontinued from study therapy.

If the subject is unable to resume both Nivolumab and Testosterone, permanent discontinuation is required.

7.5 Discontinuation Criteria

Treatment with Nivolumab and Testosterone should be permanently discontinued for any of the following:

- Any Grade 2 drug-related uveitis or eye pain or blurred vision that does not respond to topical therapy and does not improve to Grade 1 severity within the re-treatment period **OR** requires systemic treatment.
- Any Grade 3 non-skin, drug-related adverse event lasting > 7 days, with the following exceptions for drug-related laboratory abnormalities, uveitis, pneumonitis, bronchospasm, diarrhea, colitis, neurologic toxicity, hypersensitivity reactions, and infusion reactions:
    - Grade 3 drug-related uveitis, pneumonitis, bronchospasm, diarrhea, colitis, neurologic toxicity, hypersensitivity reaction, or infusion reaction of any duration requires discontinuation.
    - Grade 3 drug-related endocrinopathies adequately controlled with only physiologic hormone replacement do not require discontinuation, except for Grade 3 adrenal insufficiency which requires permanent discontinuation.
    - Grade 3 drug-related laboratory abnormalities do not require treatment discontinuation except:
        - Grade 3 drug-related thrombocytopenia > 7 days or associated with bleeding requires discontinuation.
        - Any drug-related liver function test (LFT) abnormality that meets the following criteria require discontinuation:
            - Grade $\geq$ 3 drug-related AST, ALT, or total bilirubin requires discontinuation.
            - Concurrent AST or ALT > 3xULN and total bilirubin > 2xULN.
- Any Grade 4 drug-related adverse event or laboratory abnormality, except for the following events which do not require discontinuation:
    - Grade 4 amylase or lipase abnormalities that are not associated with symptoms or clinical manifestations of pancreatitis.
    - Isolated Grade 4 electrolyte imbalances/abnormalities that are not associated with clinical sequelae and are corrected with supplementation/appropriate management within 72 hours of their onset.
    - Grade 4 neutropenia $\leq$ 7 days.
    - Grade 4 lymphopenia or leukopenia.
    - Grade 4 drug-related endocrinopathy AEs, such as, hyper- or hypothyroidism, or glucose intolerance, that resolve or are adequately controlled with physiologic hormone replacement (corticosteroids, thyroid hormones) or glucose-controlling agents, respectively, may not require discontinuation after discussion with and

approval from the investigator. Grade 4 drug-related adrenal insufficiency or hypophysitis requires discontinuation regardless of control with hormone replacement.

- Any dosing interruption lasting > 10 weeks unless the investigator is consulted and agrees with the rationale for resuming therapy after a delay > 10 weeks. Note that tumor assessments should continue as per protocol even if dosing is interrupted.
- Any adverse event, laboratory abnormality, or intercurrent illness which, in the judgment of the Investigator, presents a substantial clinical risk to the subject with continued Nivolumab dosing.

7.6 Treatment of Nivolumab-Related Infusion Reactions

Since Nivolumab contains only human immunoglobulin protein sequences, each is unlikely to be immunogenic and induce infusion or hypersensitivity reactions. However, if such a reaction were to occur, it might manifest with fever, chills, rigors, headache, rash, pruritus, arthralgias, hypo- or hypertension, bronchospasm, or other symptoms. Infusion reactions should be graded according to the most current NCI CTCAE guidelines.

Treatment recommendations are provided below and may be modified based on local treatment standards and guidelines as appropriate:

For Grade 1 symptoms: (Mild reaction; infusion interruption not indicated; intervention not indicated)

Remain at bedside and monitor subject until recovery from symptoms. The following prophylactic premedications are recommended for future infusions: diphenhydramine 50 mg (or equivalent) and/or paracetamol 325 to 1000 mg (acetaminophen) at least 30 minutes before additional nivolumab administrations.

For Grade 2 symptoms: (Moderate reaction requires therapy or infusion interruption but responds promptly to symptomatic treatment [e.g. antihistamines, non-steroidal anti-inflammatory drugs, narcotics, corticosteroids, bronchodilators, IV fluids]; prophylactic medications indicated for up to 24 hours).

Stop the Nivolumab infusion, begin an IV infusion of normal saline, and treat the subject with diphenhydramine 50 mg IV (or equivalent) and/or paracetamol 325 to 1000 mg (acetaminophen); remain at bedside and monitor subject until resolution of symptoms. Corticosteroid or bronchodilator therapy may also be administered as appropriate. If the infusion is interrupted, then restart the infusion at 50% of the original infusion rate when symptoms resolve; if no further complications ensue after 30 minutes, the rate may be increased to 100% of the original infusion rate. Monitor subject closely. If symptoms recur then no further Nivolumab will be administered at that visit. Administer diphenhydramine 50 mg IV, and remain at bedside and monitor the subject until resolution of symptoms. The amount of study drug infused must be recorded on the electronic case report form (eCRF). The following prophylactic premedications are recommended for future infusions: diphenhydramine 50 mg (or equivalent) and/or paracetamol 325 to 1000 mg (acetaminophen) should be administered at least 30 minutes before additional Nivolumab or administrations. If necessary, corticosteroids (recommended dose: up to 25 mg of IV hydrocortisone or equivalent) may be used.

For Grade 3 or Grade 4 symptoms: (Severe reaction, Grade 3: prolonged [i.e. not rapidly responsive to symptomatic medication and/or brief interruption of infusion]; recurrence of symptoms following initial improvement; hospitalization indicated for other clinical

sequelae [e.g. renal impairment, pulmonary infiltrates]). Grade 4: (life-threatening; pressor or ventilatory support indicated).

Immediately discontinue infusion of Nivolumab. Begin an IV infusion of normal saline, and treat the subject as follows. Recommend bronchodilators, epinephrine 0.2 to 1 mg of a 1:1,000 solution for subcutaneous administration or 0.1 to 0.25 mg of a 1:10,000 solution injected slowly for IV administration, and/or diphenhydramine 50 mg IV with methylprednisolone 100 mg IV (or equivalent), as needed. Subject should be monitored until the investigator is comfortable that the symptoms will not recur. Nivolumab will be permanently discontinued. Investigators should follow their institutional guidelines for the treatment of anaphylaxis. Remain at bedside and monitor subject until recovery from symptoms. In the case of late-occurring hypersensitivity symptoms (e.g. appearance of a localized or generalized pruritis within 1 week after treatment), symptomatic treatment may be given (e.g. oral antihistamine, or corticosteroids).

7.7 Treatment Beyond Disease Progression

Accumulating evidence indicates a minority of subjects treated with immunotherapy may derive clinical benefit despite initial evidence of PD, as described by irRECIST criteria (Nishino et al, Clin Cancer Res; 19(14) July 15, 2013). Subjects will be permitted to continue treatment beyond initial investigator-assessed progression without reconsent as long as they meet the following criteria:

- Investigator-assessed clinical benefit and subject is tolerating study drug.
- The assessment of clinical benefit should take into account whether the subject is clinically deteriorating and unlikely to receive further benefit from continued treatment.
- Subjects should discontinue study therapy upon evidence of further progression, defined as an additional 10% or greater increase in tumor burden volume from time of initial progression (including all target lesions and new measurable lesions).
- Per irRECIST criteria, new lesions are considered measurable at the time of initial progression if the longest diameter is at least 10 mm by CT scan (except for pathological lymph nodes, which must have a short axis of at least 15 mm). Any new lesion considered non-measurable at the time of initial progression may become measurable and therefore included in the tumor burden measurement if the longest diameter increases to at least 10 mm by CT scan (except for pathological lymph nodes, which must have an increase in short axis to at least 15 mm). In situations where the relative increase in total tumor burden by 10% is solely due to inclusion of new lesions which become measurable, these new lesions must demonstrate an absolute increase of at least 5 mm. Further bone disease progression is defined as additional two or more new bone lesions noted on bone scans compared to the time of PCWG3-defined progression of bone disease.

Testosterone Only Phase:

During the Testostone-only lead-in phase (12 weeks), patients may experience PSA progression in the presence or absence of radiographic progression. Patients will be allowed to remain on study in the following circumstances:

PSA Progression Only:

In the setting of a rising PSA (including patients with PSA progression [PCWG3 criteria]) in the absence of radiographic progression (RECIST 1.1 Criteria), patients without clinical symptoms

of disease progression will be allowed to remain on study. If the patient is not deriving clinical benefit, patients may be removed from the study at the discretion of the treating physician.

Radiographic Progression:
Patients with radiographic progression (per RECIST 1.1 Criteria) determined on post-testosterone, restaging imaging may remain on study except in *ANY* of the following circumstances:

- Patient is not deriving clinical benefit as determined by the treating physician
- Development of symptoms of clinical progression (any of the below):
  - New bony pain.
  - Risk of impending fracture.
  - Pending spinal cord compression.
  - Obstructive urinary symptoms.
  - Decline in performance status from baseline assessment.
  - Development of new liver or brain metastases.

For patients that continue on study after radiographic progression (as documented on initial imaging), restaging must occur between 6-7 weeks +/- 5 days from prior imaging. Subjects should discontinue study therapy upon evidence of further progression, defined as an additional 10% or greater increase in tumor burden volume from time of initial progression (i.e. post testosterone only imaging). Subsequent imaging will continue as per protocol and irRECIST criteria should be used to assess further progression of the Testosterone + Nivolumab combination.

7.8 Immunotherapy Adverse Event Management

Because of the potential for clinically meaningful Nivolumab-related AEs requiring early recognition and prompt intervention, management algorithms have been developed for suspected pulmonary toxicity, GI toxicity, hepatotoxicity, endocrinopathy, skin toxicity, neurological toxicity, and renal toxicity (Nivolumab IB).

These adverse event management algorithms are included in Appendix A.

7.9 Removal of Subjects from the Study, Therapy Assessment

7.9.1 Subject Withdrawal

Single subject termination is by definition when the patient is withdrawn or when the patient has died. The study termination page in the eCRF must be completed.

The Investigator also has the right to withdraw subjects from the study in the event of:

- Occurrence of an exclusion criterion, which is clinically relevant and affects the subject's safety, and discontinuation is considered necessary by the Investigator and/or the Sponsor.
- Therapeutic failure requiring urgent additional medication (if applicable)
- Occurrence of AEs, if discontinuation of study medication is considered necessary by the Investigator and/or Subject (if applicable).
- Intake of non-permitted concomitant medication as defined in Appendix A where the predefined consequence is withdrawal from the study.

- Progression of disease (subjects will only come off study after meeting PCWG3 criteria for radiographic progression and not for PSA) except as described in Section 7.7.
- Lack of subject compliance.
- Protocol violation.

7.10 Treatment Compliance

Trained medical personnel will administer Nivolumab and dispense other study medications. Treatment compliance will be monitored by drug accountability, as well as by recording administration of all medications in the CRF. The date and the exact dosage amount given at each infusion will be recorded. Any missed doses will be recorded. In case the treatment has to be interrupted during an infusion and the dosing is not resumed, the medical personnel should evaluate the percentage of dose received by the patient and document it in the patient record. Any reason for non-compliance should also be documented.

7.11 Destruction of Study Drug

Investigator drug destruction is allowed provided the following minimal standards are met:

- On-site disposal practices must not expose humans to risks from the drug.
- On-site disposal practices and procedures are in agreement with applicable laws and regulations, including any special requirements for controlled or hazardous substances.
- Written procedures for on-site disposal are available and followed. The procedures must be filed with the Sponsor SOPs and a copy provided to BMS upon request.
- Records are maintained that allow for traceability of each container, including the date disposed of, quantity disposed, and identification of the person disposing the containers. The method of disposal, ie, incinerator, licensed sanitary landfill, or licensed waste disposal vendor must be documented.
- Accountability and disposal records are complete, up-to-date, and available for BMS to review throughout the clinical trial period as per the study agreement. A copy of the drug destruction certificate should be maintained for provision to BMS at the end of the study.

If conditions for destruction cannot be met, please contact BMS.

It is the Investigator’s responsibility to arrange for disposal of all empty containers, provided that procedures for proper disposal have been established according to applicable federal, state, local, and institutional guidelines and procedures, and provided that appropriate records of disposal are kept.

7.12 Return of Study Drug

It is the Investigator’s responsibility to arrange for destruction of drug upon completion or termination of the study and disposal of all empty containers, provided that procedures for proper disposal have been established according to applicable federal, state, local, and institutional guidelines and procedures, and provided that appropriate records of disposal are kept.

8. ADVERSE EVENTS

8.1 Definitions

8.1.1 Adverse Event (AE)

An AE is defined as any untoward medical occurrence in a patient administered a medicinal product that does not necessarily have a causal relationship with this treatment. An AE can, therefore, be any unfavorable and unintended sign (including an abnormal laboratory finding), symptom, or disease temporally associated with the use of an investigational product, whether or not related to the investigational medicinal product. This includes an exacerbation of pre-existing conditions or events, intercurrent illnesses, drug interaction, or the significant worsening of the indication under investigation that is not recorded elsewhere on the eCRF under specific efficacy assessments. Anticipated fluctuations of pre-existing conditions, including the disease under study, that do not represent a clinically significant exacerbation or worsening are not considered AEs.

It is the responsibility of the Investigator to document all AEs that occur during the study. AEs should be elicited by asking the patient a non-leading question (e.g. “Have you experienced any new or changed symptoms since we last asked/since your last visit?”). The existence of an AE may be concluded from a spontaneous report of the patient; from the physical examination; or from special tests such as the ECG, laboratory assessments, or other study-specified procedure (source of AE). Symptoms reported spontaneously by the patient during the physical examination would also qualify as an AE (and hence documented on the AE eCRF, not on the physical examination eCRF, which is reserved for physical signs or findings).

8.1.2 Serious Adverse Event (SAE)

An SAE is any untoward medical occurrence that occurs at any dose (or, occurs after informed consent is given and prior to dosing if the SAE is related to a study procedure) that:

- Results in death. Any event resulting in death during the reporting period (from date of first dose of study drug through 100 days after last dose) must be treated as an SAE and reported as such. An event related to a study procedure that occurs after informed consent, but prior to dosing that results in death must also be reported as an SAE.
- Is life-threatening (patient is at immediate risk of death from the event as it occurred).
- Requires in-patient hospitalization (formal admission to a hospital for medical reasons) or prolongation of existing hospitalization.
- Results in persistent or significant disability/incapacity.
- Results in a congenital anomaly or birth defect.

Important medical events that may not result in death, are not life-threatening, or do not require hospitalization may be considered SAEs when, based on appropriate medical judgment, they may jeopardize the patient and may require medical or surgical intervention to prevent one of the outcomes listed in this definition. Examples of such

events include allergic bronchospasm requiring intensive treatment in an emergency room or at home or the development of drug dependency or drug abuse.

8.1.2.1 Events or Outcomes Not Qualifying as Serious Adverse Events

The following are not considered SAEs and therefore do not need to be reported as such:

- Pre-planned or elective hospitalization including social and/or convenience situations (e.g. respite care).
- Hospital visits of less than 24 hours duration (e.g. patient presents to the emergency room, but is not admitted to a ward).
- Overdose of either BMS study drug or concomitant medication unless the event meets SAE criteria (e.g. hospitalization). However, the event should still be captured as a nonserious AE on the appropriate eCRF page.
- Events of progression of the patient's underlying cancer as well as events clearly related to progression of the patient's cancer (signs and symptoms of progression) should not be reported as a serious adverse event unless the outcome is fatal within the safety reporting period. If the event has a fatal outcome within the safety reporting period, then the event of Progression of Disease must be recorded as an AE and as a SAE with CTCAE Grade 5 (fatal outcome) indicated.

8.1.3 Progression of Malignancy

Progression of a patient's malignancy should not be considered an AE or SAE, unless in the Investigator's opinion, study treatment resulted in an exacerbation of the patient's condition. If disease progression results in death or hospitalization while on study or within 100 days of the last dose, progressive disease will be considered an SAE.

8.1.4 Life-threatening Events

A life-threatening event is any AE that places the patient at immediate risk of death from the reaction as it occurs. It is not a reaction that had it occurred in a more severe form, might have caused death.

8.1.5 Hospitalization or Prolongation of Hospitalization

Hospitalization encompasses any inpatient admission (even for less than 24 hours) resulting from a precipitating, treatment-emergent adverse event. For chronic or long-term patients, inpatient admission also includes transfer within the hospital to an acute or intensive care inpatient unit. Hospitalizations for administrative reasons or a non-worsening preexisting condition should not be considered AEs (e.g. admission for workup of a persistent pretreatment laboratory abnormality, yearly physical exam, protocol-specified admission, elective surgery). Pre-planned treatments or surgical procedures should be noted in the baseline documentation. Hospitalization because of an unplanned event will be deemed an SAE.

Prolongation of hospitalization is any extension of an inpatient hospitalization beyond the stay anticipated or required for the original reason for admission.

8.1.6 Significant Disability

Disability is a substantial disruption of the patient's ability to conduct normal life functions.

8.1.7 Pregnancy

Male participants should refrain from fathering a child or donating sperm during the study and for 7 months following the last dose of nivolumab.

Pregnancy of the patient's partners is not considered to be an adverse event. However, the outcome of all pregnancies (spontaneous miscarriage, elective termination, ectopic pregnancy, normal birth or congenital abnormality) should, if possible, be followed up and documented.

The outcome of any conception occurring from the date of the first dose until 7 months *after the last dose* should be followed up and documented.

All outcomes of pregnancy should be reported to BMS.

8.1.8 Medical Significance

An event that is not fatal or life-threatening and that does not necessitate hospitalization may be considered serious if, in the opinion of the Investigator, it jeopardizes the patient's status and might lead to medical or surgical intervention to prevent any of the outcomes described in section 7.1.2. Such medically significant events could include allergic bronchospasm requiring intensive treatment in the emergency room or at home, blood dyscrasias that do not result in inpatient hospitalization, or the development of drug dependency or abuse.

8.1.9 Deaths

All deaths that occur during the study, or within the protocol-defined 100-day post-study follow-up period after the administration of the last dose of study treatment, must be reported as follows:

- Death that is clearly the result of disease progression should be reported to the study monitor at the next monitoring visit, be documented, and reported as an SAE.
- Where death is not due (or not clearly due) to progression of the disease under study, the AE causing the death must be reported to the study monitor as a SAE within **24 hours**. The report should contain a comment regarding the co-involvement of progression of disease, if appropriate, and should assign main and contributory causes of death.
- Deaths with an unknown cause should be reported as a SAE., but followup should be done to determine the cause of death. If a cause of death is determined, the event term of "death" must be updated at that time. A post mortem maybe helpful in the assessment of the cause of death, and if performed a copy of the post-mortem results should be forwarded to BMS within the usual timeframes.

The most recent version of the NCI CTCAE handbook will be used for adverse event descriptions and grading.

All AEs (including SAEs and AESIs) occurring during the study are to be followed up in accordance with good medical practice until resolved; judged no longer clinically significant; or, if a chronic condition, until fully characterized through 100 days after the last dose of study drug. Any SAEs, AESIs, and treatment-related Grade 3/4 AEs must be followed

until resolution or stabilization, or until lost to follow-up. After the 100-day window, treatment-related SAEs and all AESIs, irrespective of causality, do not need to be reported.

8.2 Expectedness

Adverse events can be considered, "expected," or, "unexpected."

8.2.1 Expected Adverse Events

Expected adverse events are those that have been previously identified as resulting from administration of the agent. An adverse event can be considered expected when it appears in the same nature severity and specificity as what is in the current adverse event list of the Investigator's Brochure.

8.2.2 Unexpected Adverse Events

An adverse event can be considered unexpected when the nature, intensity or frequency of which is not consistent with the current adverse event list of the Investigator's Brochure, contact the lead site, principal investigator or sponsor to confirm unexpected adverse events when necessary.

8.3 Recording and Grading

8.3.1 Recording

All observed or volunteered adverse events, regardless of treatment group, severity, suspected causal relationship, expectedness, or seriousness will be documented.

A clinically significant change in a physical examination finding or an abnormal test result (i.e., laboratory, X-ray, EKG) should be recorded as an AE, if it:

- Is associated with accompanying symptoms.
- Is suggestive of organ toxicity.
- Requires additional diagnostic testing or medical or surgical intervention.
- Leads to a change in study dosing or discontinuation from the study.
- Is considered clinically significant by the Investigator.

An abnormal test result that is subsequently determined to be in error does not require recording as an adverse event, even if it originally met one or more of the above criteria.

8.3.2 Grading Severity

All adverse events will be graded for intensity on a scale of 0 to 5. Severity grades will be recorded and based on the most recent version of the NCI CTCAE handbook.

8.3.3 Attributing Causality

Medical judgment should be used to determine the cause of the AE considering all relevant factors such as, but not limited to, the underlying study indication, coexisting disease, concomitant medication, relevant history, pattern of the AE, temporal relationship to the study medication, dechallenge or rechallenge with the study drug (Table 3).

Not Related To Study Drug	• An AE that is clearly due to extraneous causes (e.g. concurrent disease, concomitant medications, disease under study, etc.)<br>• It does not follow a reasonable temporal sequence from administration of the study drug.<br>• It does not follow a known pattern of response to study drug<br>• It does not reappear or worsen when study drug is restarted.<br>• An alternative explanation is likely, but not clearly identifiable.
Related to Study Drug	• An AE that is difficult to assign to alternative causes.<br>• It follows a strong or reasonable temporal sequence from administration of study drug.<br>• It could not be reasonably explained by the patient's clinical state, concurrent disease, or other concomitant therapy administered to the patient.<br>• It follows a known response pattern to study drug.<br>• It is confirmed with a positive rechallenge or supporting laboratory data.

Table 3. *Relationship of Adverse Event to Study Drug*

8.4 Reporting Adverse Events

8.4.1 Reporting Serious Adverse Events

Following the subject's written consent to participate in the study, all SAEs, whether related or not related to study drug, must be collected, including those thought to be associated with protocol-specified procedures. All SAEs must be collected that occur within 100 days of discontinuation of dosing.

All SAEs must be collected that occur during the screening period. If applicable, SAEs must be collected that relate to any protocol-specified procedure (e.g. a follow-up skin biopsy). The Investigator should report any SAE that occurs after these time periods that is believed to be related to study drug or protocol-specified procedure up to and including 100 days after the last dose received.

All SAEs that occur following the subject's written consent to participate in the study through 100 days of discontinuation of dosing must be reported to BMS Worldwide Safety, (Email: worldwide.safety@BMS.com; Fax: 1(609) 818-3804) whether related or not related to study drug. If applicable, SAEs must be collected that relate to any later protocol-specified procedure (e.g. a follow-up skin biopsy).

An appropriate SAE form (e.g. ex-US = CIOMS form or USA = Medwatch form) should be used to report SAEs to BMS. The BMS protocol ID number must be included on whatever form is submitted by the Sponsor/Investigator.

- The CIOMS form is available at: http://www.cioms.ch/index.php/cioms-form-i
- The MedWatch form is available at: https://www.fda.gov/downloads/AboutFDA/ReportsManualsForms/Forms/UCM048334.pdf

SAEs, whether related or not related to study drug, and pregnancies must be reported to BMS within 24 hours. SAEs must be recorded on either CIOMS or MedWatch form & pregnancies must be reported on a Pregnancy Surveillance Form or can be submitted on the aforementioned SAE form to BMS.

The Sponsor will reconcile the clinical database SAE cases (case level only) transmitted to BMS Global Pharmacovigilance (Worldwide.Safety@bms.com).

Frequency of reconciliation should be every 3 months and prior to the database lock or final data summary. BMS Global Pharmacovigilance will email, upon request from the Investigator, the Global Pharmacovigilance reconciliation report.

Requests for reconciliation should be sent to aepbusinessprocess@bms.com. The data elements listed on the Global Pharmacovigilance reconciliation report will be used for case identification purposes. If the Investigator determines a case was not transmitted to BMS Global Pharmacovigilance, the case should be sent immediately to BMS.

IND application sponsors are required to notify FDA in a written safety report of:

- Any adverse experience associated with the use of the drug that is both serious and unexpected or,
- Any findings from tests in laboratory animals that suggest a significant risk for human subjects including reports of mutagenicity, teratogenicity, and carcinogenicity.

Adverse event means any untoward medical occurrence associated with the use of a drug in humans, whether or not considered drug related.

Suspected adverse reaction means any adverse event for which there is a reasonable possibility that the drug caused the adverse event. For the purposes of IND safety reporting, "reasonable possibility" means there is evidence to suggest a causal relationship between the drug and the adverse event. A suspected adverse reaction implies a lesser degree of certainty about causality than an adverse reaction.

Adverse reaction means any adverse event caused by a drug. Adverse reactions are a subset of all suspected adverse reactions where there is reason to conclude that the drug caused the event.

Unexpected adverse event or suspected adverse reaction refers to an event or reaction that is not listed in the Investigator's Brochure or is not listed at the specificity or severity that has been observed; or, if an Investigator's Brochure is not

required or available, is not consistent with the risk information described in the general investigational plan or elsewhere in the current IND application.

Serious adverse event or suspected adverse reaction refers to an event or reaction that, in the view of either the Investigator or Sponsor, results in any of the following outcomes:

- Death.
- A life-threatening adverse event.
- In-patient hospitalization or prolongation of existing hospitalization.
- A persistent or significant incapacity or substantial disruption of the ability to conduct normal life functions.
- A congenital anomaly or birth defect.

A life-threatening adverse event or suspected adverse reaction is considered "life-threatening" if, in the view of the investigator or sponsor, its occurrence places the patient or subject at immediate risk of death. It does not include an adverse event or suspected adverse reaction that, had it occurred in a more severe form, might have caused death.

Important medical events that may not result in death, be life-threatening, or require hospitalization may be considered serious when, based upon appropriate medical judgment, they may jeopardize the patient or research subject and may require medical or surgical intervention to prevent one of the outcomes listed as serious.

Mandatory Safety Reporting

• Initial reporting: IND application sponsor must report any suspected adverse reaction or adverse reaction to study treatment that is both serious and unexpected.

Unexpected serious suspected adverse reactions suggesting significant risk to human subjects must be reported to FDA as soon as possible but no later than within 15 calendar days following the sponsor's initial receipt of the information. Unexpected fatal or life-threatening suspected adverse reactions represent especially important safety information and must be reported to FDA as soon as possible but no later than 7 calendar days following the sponsor's initial receipt of the information.

• Follow-up reporting: Any relevant additional information obtained by the sponsor that pertains to a previously submitted IND safety report must be submitted as a Follow-up IND Safety Report. Such report should be submitted without delay, as soon as the information is available but no later than 15 calendar days after the sponsor receives the information.

All IND safety reports must be submitted on Form 3500A and be accompanied by Form 1571. The type of report (initial or follow-up) should be checked in the respective boxes on Forms 3500A and 1571.

The submission must be identified as:

- "IND safety report" for 15-day reports or,

- “7-day IND safety report” for unexpected fatal or life-threatening suspected adverse reaction reports or,
- “Follow-up IND safety report” for follow-up information.

The report must be submitted to an appropriate Review division that has the responsibility to review the IND application under which the safety report is submitted. Each submission to this IND must be provided in triplicate (original plus two copies). Send all submissions to the following address:

Food and Drug Administration
Center for Drug Evaluation and Research
Division of Oncology Products 1
5901-B Ammendale Road
Beltsville, MD 20705-1266

8.4.2 Reporting SAEs at Multi-Site/Participating Institutions
SAEs should be reported to the Lead site and BMS.

9. CRITERIA FOR OUTCOME ASSESSMENT/THERAPEUTIC RESPONSE

9.1 Outcome Assessment

All baseline evaluations will be performed as closely as possible to the beginning of treatment (within 7 days). For subsequent evaluations, the method of assessment and techniques will be the same as those used at baseline.

Note: Any bone lesions, however, that are identified on the baseline CT or MRI studies and judged as providing meaningful information about nontarget disease status on the basis of their size or other features should be considered for inclusion in the follow-up assessments of nontarget lesions by the same imaging modality.

- Conventional CT, NM Bone Scan.

  CT Chest, Abdomen, Pelvis and NM Bone scans will be used to measure tumor response as a secondary endpoint per RECIST 1.1 Criteria.

- Tumor markers.

  PSA measurements will be used to assess the primary endpoint.

9.1.1 Primary Endpoint
The primary endpoint is defined as a PSA_{50} response, defined as a ≥ 50% decline in PSA from baseline, confirmed with a second measurement at least 4 weeks later.

9.1.2 Secondary Endpoints

9.1.2.1 Safety
This endpoint is defined as incidence of grade 3-5 toxicities based upon the most current CTCAE standard grading scales.

9.1.2.2 PSA Progression-Free Survival (PFS)
PSA progression (PSA progression-free survival; PSA–PFS) will be defined

per PCWG3 guidelines.

For those subjects showing an initial decline in PSA from baseline, is defined as an increase in PSA that is ≥ 25% and ≥ 2 ng/mL above the nadir, and which is confirmed by a second value ≥ 3 weeks later (i.e. a confirmed rising trend).

For those subjects with no decline in PSA from baseline, is defined as an increase in PSA that is ≥ 25% and ≥ 2 ng/mL after 12 weeks.

9.1.2.3 Progression–free Survival

Time to radiographic or clinical progression or death, whichever comes first.

Based on RECIST version 1.1 and PCWG3 definitions including:

- Progression of soft tissue lesions according to RECIST 1.1 Criteria.
- Progression of bone lesions detected with bone scan according to PCWG3 criteria.
- Radiologically-confirmed spinal cord compression or pathological fracture due to malignant progression, or other clinical event deemed to be cancer-related.

9.1.2.4 Objective Response Rate

Defined as the proportion of patients achieving a complete/partial response in target lesions (RECIST 1.1 Criteria).

9.1.2.5 Durable Progression-free Survival (durable-PFS)

Durable PFS will be defined as the proportion of patients without clinical or radiographic progression, as defined by PCWG3 criteria, or death at 24 weeks from the start of treatment.

9.1.2.6 Overall Survival (OS)

Overall survival will be defined as the time from study enrollment to death or up to 2 years after last dose.

This will be summarized using a Kaplan-Meier curve.

9.2 Therapeutic Response

Response and progression will be evaluated in this study using a combination of the international criteria proposed by the Response Evaluation Criteria in Solid Tumors (RECIST) Committee(42) and the guidelines for prostate cancer endpoints developed by the Prostate Cancer Clinical Trials Working Group (PCWG3).(43)

Patients will need to be re-evaluated for response every cycle according to the guidelines below.

9.2.1 PSA

Perform PSA testing at a minimum of 1-week intervals with the threshold PSA level at 2.0 ng/mL. To report PSA-based outcomes, PCWG3 recommends that the percent of change in PSA from baseline to 12 weeks (or earlier for those who discontinue

therapy) and the maximum decline in PSA that occurs at any point after treatment be reported for each patient using a waterfall plot. PSA measurements obtained during the first 12 weeks should not be used as the sole criterion for clinical decision making.

9.2.2 Measurable Disease

According to RECIST 1.1, measurable disease is defined as at least 1 lesion > 10mm in its longest diameter as measured with conventional techniques (i.e. CT with slice cut of 5 mm, MRI). All tumor measurements will be taken using a ruler or calipers and recorded in millimeters (or decimal fractions of centimeters). To be considered pathologically enlarged and measurable, a lymph node must be ≥ 15 mm in short axis when assessed by CT scan (CT scan slice thickness recommended to be no greater than 5 mm).

9.2.3 Nonmeasurable Disease

Following RECIST 1.1, all other lesions (or sites of disease) will be considered nonmeasurable disease. This includes small lesions (longest diameter < 10 mm using spiral CT scan), lymph nodes (shortest diameter ≥ 10 mm to < 15 mm) and any of the following:

- Bone lesions.
- Ascites.
- Pleural or pericardial effusion.
- Lymphangitis cutis or pulmonis.
- Abdominal masses that are not confirmed and followed by imaging techniques.
- Cystic lesions.
- Lesions occurring within a previously irradiated area unless they are documented as new lesions since the completion of radiation therapy.

Note. If only a single, asymptomatic bone lesion is present at baseline, and will be irradiated, the metastatic nature of this lesion must be confirmed by X-ray, CT, or MRI.

9.2.4 Target (nodal and visceral) Lesions

Following RECIST 1.1 Criteria, progression in a nodal or visceral site (i.e. liver and lung) is sufficient to document disease progression. The presence or absence of nodal and visceral disease before and after treatment should be recorded separately.

All measurable lesions (up to a maximum of 2 lesions per organ and 5 lesions in total) will be identified as target lesions to be measured and recorded at baseline. The target lesions should be representative of all involved organs. Target lesions will be selected on the basis of size (i.e. the largest area) and suitability for accurate, repeated measurements (either by imaging techniques or clinically). The sum of the longest diameter (LD) of all non-nodal and shortest diameter (nodal) target lesions will be calculated and reported as the baseline sum LD. The baseline sum LD will be used as a reference by which to characterize the objective tumor response.

Because small lymph nodes are difficult to measure accurately and may not be malignant, to be considered pathologically enlarged and measurable, a lymph node

must be ≥ 15 mm in short axis when assessed by CT scan (CT scan slice thickness recommended to be no greater than 5 mm).

9.2.5 Bone Lesions

When the bone scan is the sole indicator of progression, disease progression in bone is defined as ≥ 2 new lesions seen on bone scan compared with a prior scan for used trial entry. In situations where scan findings suggest a flare reaction or where new lesion(s) may represent trauma, confirm these results with other imaging modalities (e.g. MRI or fine-cut CT). If many new areas of uptake are observed, confirmation is generally not necessary.

9.2.6 Nontarget Lesions

All other lesions (or sites of disease) will be identified as nontarget lesions and recorded at baseline. Nontarget lesions will include measurable lesions that exceed the maximum number per organ (2) or total of all involved organs (5), as well as nonmeasurable lesions. The presence or absence of these lesions will be recorded on the CRF and should be evaluated at the same assessment time points as all target lesions.

9.2.7 New Lesions

The appearance of new malignant lesions denotes disease progression. There are no specific criteria for the identification of new radiographic lesions; however, the finding of a new lesion should be unequivocal, i.e. not attributable to differences in scanning technique, change in imaging modality or findings thought to represent something other than tumor (for example, some "new" bone lesions may be simply healing or flare of pre-existing lesions).

9.3 Response Criteria for Primary and Secondary Endpoints

9.3.1 Measurable Soft-tissue Lesions

When evaluating soft-tissue lesions, the definitions in Table 4 apply.

Table 4. *RECIST 1.1 Response Criteria for Target Lesions*

Response	Evaluation of Target Lesions
Complete response (CR)	Disappearance of all target lesions. Any pathological lymph nodes (whether target or non-target) must have reduction in short axis to < 10 mm.
Partial response (PR)	At least a 30% decrease in the sum of diameters of target lesions, taking as reference the baseline sum diameters.
Progressive disease (PD)	At least a 20% increase in the sum of diameters of target lesions, taking as reference the smallest sum on study (this includes the baseline sum if that is the smallest on study). In addition to the relative increase of 20%, the sum must also demonstrate an absolute increase of ≥ 5 mm. (Note: the appearance of one or more new lesions is also considered progression).

Stable disease (SD)	Neither sufficient shrinkage to qualify for PR nor sufficient increase to qualify for PD, taking as reference the smallest sum diameters while on study.

In some circumstances, it may be difficult to distinguish residual disease from normal tissue. When the evaluation of complete response depends on this determination, it is recommended that the residual lesion be investigated (e.g. fine needle aspirate or biopsy) before confirming the complete response status.

Changes in nodal and visceral sites should be recorded and reported separately, and lymph nodes in the pelvis must measure ≥ 15 mm in shortest diameter to be considered target lesions. Complete elimination of disease at a particular site should be recorded separately. Any favorable change should be confirmed using a second follow-up scan.

9.3.2 PSA

For each patient, use a waterfall plot to report the percent change in PSA from baseline to 12 weeks (or earlier for those who discontinue therapy) and the maximum decline in PSA that occurs at any point after treatment. We will also report the proportion of patients to achieve a 50% of greater decrease in PSA from baseline (i.e. PSA_{50} response rate).

9.3.3 Bone

Record post-treatment changes as either “no new lesions” or “new lesions.”

In the absence of clearly worsening soft-tissue (nodal and visceral) disease or disease-related symptoms, progression at the first scheduled assessment should be confirmed on a second scan performed ≥ 6 weeks later. In the rare case where visible lesions disappear, this too should be confirmed.

9.3.4 Nontarget Lesions

When assessing nontarget lesions, the definitions in Table 5 will apply.

Table 5. *RECIST 1.1 Response Criteria for Nontarget Lesions*

**Response**	**Evaluation of Nontarget Lesions**
Complete response (CR)	The disappearance of all nontarget lesions and normalization of tumor marker levels. All lymph nodes must be non-pathological in size (< 10 mm short axis).
Non-CR/non-PD	The persistence of one or more nontarget lesions and/or maintenance of tumor marker levels above the normal limits.
Progressive disease (PD)	The appearance of one or more new lesions and/or unequivocal progression of existing nontarget lesions.

9.4 Criteria for Progressive Disease.

9.4.1 Measurable Soft-tissue Lesions
When evaluating soft-tissue lesions, the definitions in Table 4 apply.

In some circumstances, it may be difficult to distinguish residual disease from normal tissue. When the evaluation of complete response depends on this determination, it is recommended that the residual lesion be investigated (e.g. fine needle aspirate or biopsy) before confirming the complete response status.

Progression in a nodal or visceral site should be defined using RECIST 1.1 Criteria (Table 4).

9.4.2 PSA
Per the PCWG3, PSA progression is defined as the date that a 25% or greater increase and an absolute increase of 2 ng/mL or more from the nadir is documented and confirmed by a second value obtained 3 or more weeks later. Where no decline from baseline is documented, PSA progression is defined as a 25% increase from the baseline value along with an increase in absolute value of 2 ng/mL or more after 12 weeks of treatment.

9.4.3 Bone
Progressing disease on bone scan is considered when at least 2 new lesions are observed. Yet, progression remains unconfirmed unless at least 2 *additional* new lesions appear at a subsequent time point.

Unless clinically indicated, there is no need to perform a follow-up bone scan before 12 weeks of treatment. To define disease progression requires a confirmatory scan (which shows additional new lesions compared with the first follow-up scan) performed ≥ 6 weeks later. When further progression is documented on the confirmatory scan, the date of progression recorded for the trial is the date of the first scan that shows the change.

9.4.4 Nontarget Lesions
When assessing nontarget lesions, the definitions in Table 5 apply.

10. DATA REPORTING AND REGULATORY REQUIREMENTS

Multicenter Guidelines
The Protocol Chair

The Protocol Chair, Dr. Mark Markowski is responsible for performing the following tasks:

- Coordinating, developing, submitting, and obtaining approval for the protocol as well as its subsequent amendments.
- Assuring that all participating institutions are using the correct version of the protocol.
- Taking responsibility for the overall conduct of the study at all participating institutions and for monitoring the progress of the study.
- Reviewing and ensuring reporting of Serious Adverse Events (SAEs).
- Reviewing data from all sites.

Lead Center

The Lead Center (Johns Hopkins University) is responsible for performing the following tasks:

- Ensuring that IRB approval has been obtained at each participating site prior to the first patient registration at that site, and maintaining copies of IRB approvals from each site.
- Managing central patient registration.
- Collecting and compiling data from each site.
- Establishing procedures for documentation, reporting and submitting of AEs and SAEs to the Protocol Chair and all other applicable parties.
- Facilitating audits by securing selected source documents and research records from participating sites for audit, or by auditing at participating sites.

Participating PCCTC Sites

Participating sites are responsible for performing the following tasks:

- Following the protocol as written, and the guidelines of Good Clinical Practice (GCP).
- Submitting data to the Lead Center.
- Registering all patients with the Lead Center by submitting patient registration form and signed informed consent promptly.
- Providing sufficient experienced clinical and administrative staff and adequate facilities and equipment to conduct a collaborative trial according to the protocol.
- Maintaining regulatory binders on site and providing copies of all required documents to the Lead Center.
- Collecting and submitting data according to the schedule specified by the protocol.

10.1 Data Entry

Data collected during this study will be entered into a secure database. Staff at Johns Hopkins University will be responsible for the initial study configuration and setup in the CRMS database and for any future changes.

10.1.1 Case Report Forms Completion

Electronic Case report forms will be generated by the coordinating center for the collection of all study data. Investigators will be responsible for ensuring that the CRFs are kept up-to-date.

The paper Eligibility Checklist CRF must be completed using black ink. Any errors must be crossed out so that the original entry is still visible, the correction clearly indicated and then initialed and dated by the individual making the correction.

eCRFs will be completed within 2 weeks of the patient coming to the clinic and all relevant supporting documentation such as scans, progress notes, nursing notes, blood work, pathology reports, etc., will be submitted via email to the SKCCC Coordinating Center Study Manager. All patient names or other identifying information will be removed prior to being sent to the Coordinating Center (SKCCC)

or non-redacted source documents can be sent via a password-protected/secured document transfer based on each institution's guidelines.

Authorized representatives of the Coordinating Center (SKCCC) may visit the satellite sites to perform audits or inspections, including source data verification. The purpose of these audits or inspections is to systematically and independently examine all trial-related activities and documents to determine whether these activities were conducted and data were recorded, analyzed, and accurately reported according to the protocol, Good Clinical Practice (GCP), and any applicable regulatory requirements.

10.1.2 Source Documents

Study personnel will record clinical data in each patient's source documents (i.e. the patient's medical record). Source documentation will be made available to support the patient research record. Study monitors will review entries on the CRFs at regular intervals, comparing the content with source documents.

10.1.3 Record Retention

The Investigator will maintain adequate and accurate records to enable the conduct of the study to be fully documented and the study data to be subsequently verified. After study closure, the Investigator will maintain all source documents, study-related documents, and the CRFs. Because the length of time required for retaining records depends upon a number of regulatory and legal factors, documents should be stored until the Investigator is notified that the documents may be destroyed. In this study, records are to be retained and securely stored for a minimum of 5 years after the completion of all study activities.

10.2 Data Management

10.2.1 Lead Research Program Coordinators

A Lead research program coordinator at the coordinating center will be assigned to the study. A Lead Research Program Coordinator will manage the study activities at each of the participating sites. The responsibilities of the Lead Research Program Coordinator include project compliance, data collection, data entry, data reporting, regulatory monitoring, problem resolution and prioritization, and coordination of the activities of the protocol team.

10.3 Study Monitoring and Quality Assurance

Regularly scheduled registration reports will be generated to monitor patient accruals and the completeness of registration data. Routine data quality reports will be generated to assess missing data and inconsistencies. Accrual rates and the extent and accuracy of evaluations and follow-up will be monitored periodically throughout the study period, and potential problems will be brought to the attention of the principal investigator for discussion and action.

Random-sample data quality and protocol compliance audits will be conducted by the study team at least once a year, more frequently if indicated. Audits by the coordinating center may entail (1) shipping source documents and research records for selected patients from participating sites to the coordinating center for audit, or (2) on-site auditing of selected patient records at participating sites.

All clinical work conducted under this protocol is subject to Good Clinical Practice (GCP) guidelines. This includes inspection of study-related records by the lead site, sponsor, its designee, or health authority representatives at any time.

Data and Safety Monitoring:
This is a DSMP Medium Risk study under the SKCCC Data and Safety Monitoring Plan (6/5/2020). Data Monitoring of this protocol will occur annually. The protocol will be monitored internally at SKCCC by the Principal Investigator. The SKCCC Compliance Monitoring Program will provide external monitoring for JHU affiliated sites in accordance with SKCCC DSMP (Version 6.0, 02/21/2019). The SMC Subcommittee will determine the level of patient safety risk and level/frequency of monitoring.

Additionally, scheduled meetings will take place monthly and will include the protocol Principal Investigator, research nurse, data manager, and, when appropriate, the collaborators, subinvestigators, and biostatistician involved with the conduct of the protocol.

During these meetings the investigators will discuss matters related to: the safety of protocol participants, validity and integrity of the data, enrollment rate relative to expectation, characteristics of participants, retention of participants, adherence to protocol (potential or real protocol violations), data completeness, and progress of data for secondary objectives.

10.4 Clinical Trial Agreement

This trial is being conducted under one or more clinical trial agreements that contain, among other terms, the publication policy, indemnity agreements, and financial arrangements for the study.

11. STATISTICAL CONSIDERATIONS

The primary endpoint will be the proportion of subjects who achieve a PSA response, defined as a 50% or greater decline in PSA from baseline, confirmed on a subsequent measurement at least 4 weeks apart. The treatment regimen would be considered of insufficient activity for further study in this population if the PSA response rate is 25% or less. The sample size is calculated to detect an improved PSA response rate from 25% (null hypothesis) to 45% (alternative). A minimax Simon two stage design is planned. A total of 23 patients will be entered in the first stage. A planned analysis will be performed 12 weeks after the last patient in the first stage receives the first dose of nivolumab. If ≤ 5 subjects have a PSA response, the study will be terminated and we will conclude the regimen is ineffective. If ≥ 6 subjects respond, then an additional 16 patients will be studied, for a total of 39 patients. If a total of ≤ 13 subjects respond in stage one and two combined, we consider this regimen ineffective. If a total of ≥ 14 respond, we conclude the regimen is promising and warrants further study. The maximum sample size will be increased to 44 patients to account for 10% possible dropouts and unevaluable patients.

This design provides 90% power to detect an absolute 20% increase in PSA response rate with a one-sided type I error of 0.1. The probability of early stopping is 0.49 under the null hypothesis that the PSA_{50} response rate is 25%.

11.1 Study Endpoints

11.1.1 Analysis of the Primary Endpoint

The primary endpoint of this study is PSA_{50} response, defined as a decrease in the PSA to 50% less than the baseline PSA upon enrollment in the trial. The decrease must be confirmed by a second measurement at least 4 weeks apart. For purposes of meeting the primary endpoint, patients will be considered to have done so if they have a PSA_{50} response only while on therapy with Testosterone +/- Nivolumab. PSA values will be measured each cycle during the trial. All patients who are administered at least one dose of Testosterone will be considered evaluable for the primary endpoint. If patients do not have follow-up PSAs after initiation of Testosterone therapy due to withdrawing consent or being lost to follow-up for example, then they will be replaced.

We will estimate the PSA_{50} response rate, along with the exact 95% confidence interval, for the population of patients.

11.1.2 Analysis of Secondary Endpoints

11.1.2.1 Safety

Patients will be assessed for toxicities at each clinical evaluation. Toxicities will graded according to the most current CTCAE standardized grading scales. The incidence of grade 3-5 toxicities will be reported. Patients will be assessed for toxicity as long as they are taking Testosterone +/- Nivolumab, and patients will continue to be followed if treatment is discontinued for toxicity until the toxicities improve to grade 1 or resolve.

Toxicities will be reported as a tabulated table by type and grade.

11.1.2.2 PSA Progression-free Survival (PSA-PFS)

A standard definition of PSA progression per PCWG3 will be used. PSA-PFS will be defined as an increase in 25% over a nadir value, confirmed by a follow-up PSA at least 4 weeks apart. If patients are removed from study prior to PSA progression, then they will be censored at that time.

We will use the Kaplan-Meier method to summarize the median PSA PFS.

11.1.2.3 Progression-free Survival (PFS)

Progression-free survival will be measured from the time of first dose to objective tumor progression as defined by RECIST 1.1 for progressive disease or death and summarized using a Kaplan-Meier curve. Progression will be assigned to the earliest observed time. Patients whose disease has not progressed at follow-up will be censored at the date when the last tumor assessment determined a lack of progression.

We will use the Kaplan-Meier method to summarize the median PFS.

11.1.2.4 Objective Response Rate (ORR)

The objective response rate is defined as the percentage of patients who achieve an objective response by RECIST 1.1 criteria (i.e. Complete response or Partial Response) to Testosterone +/- Nivolumab.

We will estimate the objective response rate, along with the exact 95% confidence interval, for the population of patients with RECIST 1.1 evaluable disease.

11.1.2.5 Durable Progression-free Survival

Durable PFS will be defined as the proportion of patients without clinical or radiographic progression or death at 24 weeks from start of treatment.

11.1.2.6 Overall Survival (OS)

Overall survival will be defined as the time from study enrollment to death or up to 2 years after the last dose of study treatment.

This will be analyzed as a Kaplan-Meier plot.

11.1.3 Analysis of Exploratory Endpoints

11.1.3.1 Estimate the Percentage of Patients with a Pathogenic Somatic or Germline Mutation in a HR or MMR Gene Mutation

The somatic DNA mutations present in the tumor will be identified through Foundation Medicine sequencing. Germline DNA mutation testing per standard of care practice will be done using clinical-grade testing in a CLIA-certified laboratory (i.e. COLOR genomics) for consenting subjects only. Patients with a germline or somatic deleterious mutation or deletion in the pre-specified gene list (BRCA1, BRCA2, ATM, CHEK2, NBN, RAD50, RAD51C, RAD51D, PALB2, MRE11, FANCA, FANCB, FANCC, FANCD2, FANCE, FANCF, FANCG, FANCI, FANCL, FANCM, MLH1, MLH3, MSH2, MSH3, MSH6, PMS1 and PMS2) will be considered biomarker positive. For biomarker positive and biomarker negative subjects, we will calculate PSA_{50} response rates with confidence intervals for hypothesis generation.

11.1.3.2 PD-1, PD-L1, CD4, CD8, FOXP3, γH2AX, RAD51, 53BP1 Protein Levels:

Subjects will have pre-treatment, on-treatment (and at progression) tumor biopsy specimens analyzed. IHC staining for PD-1 and PD-L1 will be performed on the pre-treatment samples and scored as <1%, 1-5%, 5-10%, >10% and the results will be associated with responses for both proteins, separately, using descriptive statistics and Fisher's Exact Tests. Immunofluorescence will be used to quantify the median intensity of each gamma-H2AX, RAD51m and 53BP1 foci per cell as well determine the median number of foci per cell on the pretreatment biopsies. Using analytical microscopy, Drs. Meeker and Heaphy have developed imaging software which can isolate single nuclei and quantify both the intensity and number of immunofluorescent foci per nucleus (i.e. per cell). Using FFPE tissue obtained at biopsy, we will examine the change in DNA damage foci number and intensity (reported as median percent change) induced by BAT +/- Nivolumab in PSA_{50} responders versus non-responders using a T-test.

Using imaging software, we will count the number of CD4+ and CD8+ lymphocytes in pre-treatment and on-treatment biopsies. We will correlate PSA_{50} responses with CD4+ and CD8+ lymphocyte tumor infiltration.

11.1.3.3 MANAs/TAAs Assay

Whole-exome sequencing will be performed on pre-treatment samples, after 12 weeks of BAT lead-in therapy, and post-progression tumor and matched normal samples. Exome data will be applied in a neoantigen prediction pipeline that evaluates antigen processing, MHC binding, and gene expression to generate neoantigens specific to the patient's HLA haplotype. Truncal neoantigens will be identified by correcting for tumor purity and ploidy. Putative neoantigens will then be used to generate peptides and stimulate autologous T cells, followed by TCR next-generation sequencing.

11.1.3.4 Cytokines and Chemokines Analysis

Milliplex human cytokine/chemokine Immunology Multiplex Assay (Millipore-Sigma) will be used to assay acute effects of BAT on production of cytokines and chemokines. Plasma samples are obtained at prior to the first BAT injection on C1D1, 3 hours after the first BAT injection (C1D1), 3 days after the first BAT injection (C1D4), prior to C2D1, and prior to C7D1. Samples will be processed and stored in the Denmeade Laboratory and analyzed by the Immune Processing Core Lab at Johns Hopkins.

11.2 Analysis Populations

11.2.1 Intent-to-treat/Response-to-treatment/Evaluable Population

All patients who meet eligibility criteria and receive at least 1 dose of testosterone will be included in the main analysis of the response rate, even if there are major protocol deviations (e.g. incorrect treatment schedule or drug administration). Each patient will be assigned to one of the following categories:

Table 6. *Categories for Response to Treatment*

Category	Response
1	Complete response
2	Partial response
3	Stable disease
4	Progressive disease
5	Early death from malignant disease
6	Early death from toxicity
7	Early death from other causes
8	Unknown (not assessable/insufficient data)

NOTE: By arbitrary convention, category 8 designates unknown status in a clinical database. Patients in response categories 4 to 6 will be considered to have treatment failure (disease progression) Patients in response categories 7 and 8 will be censored (i.e. stable disease) or considered disease progression at the discretion of the PI.

Conclusions are to be based on the population of all eligible patients. Subanalyses may be performed on various subsets of patients, such as those with no major protocol deviations or those who continued in the study for the entire treatment period (i.e. did not withdraw prematurely). Subanalysis will not serve as the basis for drawing conclusions concerning treatment efficacy.

11.2.2 Safety Population

All patients enrolled in the study will be included in the safety analysis population and considered evaluable for toxicity and safety from the time of their first dose. Demographic and baseline characteristics for the safety population will be summarized by number and percent for categorical data (e.g. sex, race/ethnicity) and by descriptive statistics for continuous data (e.g. weight, vital signs, EKG readings, disease status).

11.3 Safety Analysis

11.3.1 Evaluation of Adverse Events

Treatment-emergent adverse events will be translated from investigator terms to MedDRA v.20.1 terminology and summarized (number and percentage of patients) for all patients who receive at least 1 dose. Adverse event summaries will be organized by body system, frequency of occurrence, intensity (i.e. severity grade), and causality or attribution. Patients who experience an adverse event more than once will be counted only once. The occurrence with the maximum severity will be used to calculate intensity.

11.3.2 Evaluation of Serious Adverse Events and Premature Withdrawals

Adverse events deemed serious and those resulting in treatment withdrawal or death will be summarized separately. Narrative paragraphs will be generated to describe the circumstances surrounding each SAE and death.

11.3.3 Evaluation of Laboratory Parameters and Assays

Selected clinical laboratory parameters will be summarized and clinically significant changes from baseline will be discussed.

11.3.4 Extent of Exposure

Treatment exposure will be summarized for all patients, including dose administration, number of cycles, dose modifications or delays, and duration of therapy.

12. PROTECTION OF HUMAN SUBJECTS

12.1 *Ethical Considerations*

This study will be conducted in compliance with the protocol, GCP guidelines established by the International Conference on Harmonization, and the ethical standards set forth in the Declaration of Helsinki 2004 (available at: www.laakariliitto.fi/e/ethics/helsinki.html).

12.2 *Protocol Amendments*

Before starting the study, the protocol must be approved by each institution's IRB or Independent Ethics Committee (IEC). Amendments to the protocol may be made only with consent of the lead site/sponsor and principal investigator and are subject to IRB approval before instituting.

12.3 *Written Informed Consent*

Before obtaining consent, members of the study team will review the rationale for the treatment program with the patient. The discussion will review the alternatives available (including hormonal therapy, chemotherapy, or supportive care as appropriate), the potential benefits of this program, the risks and the probability of their occurrence, and the procedures to minimize these risks. Should an adverse event occur, the provisions available to ensure medical intervention will also be reviewed. Why the risks are reasonable in relation to the anticipated benefits, incentives, or costs that will or may be incurred as a result of participating in the study, as well as the efforts to maintain confidentiality, will also be discussed with the patient.

Patients will be required to sign and date (in duplicate) a statement of informed consent that meets the requirements of the Code of Federal Regulations (Federal Register Vol. 46, No. 17, January 27, 1981, part 50) and the IRB. The medical record will include a statement that written informed consent was obtained (and document the date that it was obtained) before the patient is enrolled in the study. The original signed document will become part of the patient's medical record, a copy will be forwarded to the lead site/sponsor pursuant to sponsor registration, and a copy will be sent home with each patient.

The consent form will include the following:

- The nature and objectives, potential toxicities, and benefits of the intended study.
- The length of therapy and likely follow-up required.
- Alternatives to the proposed therapy (including available standard and investigational therapies).
- The name of the investigator(s) responsible for the protocol.
- The right of the patient to accept or refuse treatment and to withdraw from participation in this study.
- Text regarding the coordinating center should be added to all institutional informed consent documents.

12.4 *Protection of Privacy*

Patients will be informed of the extent to which their confidential health information generated from this study may be used for research purposes. After this discussion, they will be asked to sign a Notice of Privacy Practice research authorization/HIPAA form. The original signed documents will become part of the patient's medical records, and each patient will receive a copy of the signed documents. The use and disclosure of protected health information will be limited to the individuals described in the research authorization form. The research authorization form must be completed by the principal investigator and approved by the IRB.

12.5 *Terminating or Modifying the Study*

Adverse event and laboratory data from this trial will be assessed by the medical monitor (Dr. Mark Markowski) on an ongoing basis. At least quarterly, data from the clinical database will be reviewed. The results of this review will be shared with all investigators either in writing or as part of a teleconference. SAEs will be reviewed as they are reported to the lead site/sponsor, and the medical monitor will make an assessment regarding the safety of continuing or modifying the study. This assessment will be shared with the investigators either in writing or as part of a teleconference as well as with BMS. Should the assessment of either the lead site/sponsor or the principal investigator be that the study should be terminated, the study will be closed to further accrual. Patients who are receiving Testosterone +/- Nivolumab will be assessed individually by the investigator to see if it is in the patients' best interest to continue, which might be the case for a patient that is responding to the intervention. Follow-up safety assessments will be performed for all patients who are terminated from the study prematurely. Any planned data disclosures will also be discussed with BMS.

13. REFERENCES

1. Siegel RL, Miller KD, Jemal A. Cancer statistics, 2016. CA: a cancer journal for clinicians 2016;66(1):7-30.
2. Scher HI, Morris MJ, Stadler WM, Higano C, Basch E, Fizazi K, Antonarakis ES, Beer TM, Carducci MA, Chi KN, Corn PG, de Bono JS, Dreicer R, George DJ, Heath EI, Hussain M, Kelly WK, Liu G, Logothetis C, Nanus D, Stein MN, Rathkopf DE, Slovin SF, Ryan CJ, Sartor O, Small EJ, Smith MR, Sternberg CN, Taplin ME, Wilding G, Nelson PS, Schwartz LH, Halabi S, Kantoff PW, Armstrong AJ. Trial Design and Objectives for Castration-Resistant Prostate Cancer: Updated Recommendations From the Prostate Cancer Clinical Trials Working Group 3. J Clin Oncol 2016;34(12):1402-1418.
3. Langeler EG, van Uffelen CJ, Blankenstein MA, van Steenbrugge GJ, Mulder E. Effect of culture conditions on androgen sensitivity of the human prostatic cancer cell line LNCaP. The Prostate 1993;23(3):213-223.
4. Kokontis JM, Hay N, Liao S. Progression of LNCaP prostate tumor cells during androgen deprivation: hormone-independent growth, repression of proliferation by androgen, and role for p27Kip1 in androgen-induced cell cycle arrest. Molecular endocrinology 1998;12(7):941-953.
5. Isaacs JT, D'Antonio JM, Chen S, Antony L, Dalrymple SP, Ndikuyeze GH, Luo J, Denmeade SR. Adaptive auto-regulation of androgen receptor provides a paradigm shifting rationale for bipolar androgen therapy (BAT) for castrate resistant human prostate cancer. The Prostate 2012;72(14):1491-1505.
6. Litvinov IV, Vander Griend DJ, Antony L, Dalrymple S, De Marzo AM, Drake CG, Isaacs JT. Androgen receptor as a licensing factor for DNA replication in androgen-sensitive prostate cancer cells. Proceedings of the National Academy of Sciences of the United States of America 2006;103(41):15085-15090.
7. Vander Griend DJ, Litvinov IV, Isaacs JT. Stabilizing androgen receptor in mitosis inhibits prostate cancer proliferation. Cell cycle 2007;6(6):647-651.
8. Hu R, Lu C, Mostaghel EA, Yegnasubramanian S, Gurel M, Tannahill C, Edwards J, Isaacs WB, Nelson PS, Bluemn E, Plymate SR, Luo J. Distinct transcriptional programs mediated by the ligand-dependent full-length androgen receptor and its splice variants in castration-resistant prostate cancer. Cancer research 2012;72(14):3457-3462.
9. Thelen P, Heinrich E, Bremmer F, Trojan L, Strauss A. Testosterone boosts for treatment of castration resistant prostate cancer: an experimental implementation of intermittent androgen deprivation. The Prostate 2013;73(15):1699-1709.
10. Haffner MC, Aryee MJ, Toubaji A, Esopi DM, Albadine R, Gurel B, Isaacs WB, Bova GS, Liu W, Xu J, Meeker AK, Netto G, De Marzo AM, Nelson WG, Yegnasubramanian S. Androgen-induced TOP2B-mediated double-strand breaks and prostate cancer gene rearrangements. Nature genetics 2010;42(8):668-675.
11. Bastus NC, Boyd LK, Mao X, Stankiewicz E, Kudahetti SC, Oliver RT, Berney DM, Lu YJ. Androgen-induced TMPRSS2:ERG fusion in nonmalignant prostate epithelial cells. Cancer research 2010;70(23):9544-9548.
12. Dong H, Strome SE, Salomao DR, Tamura H, Hirano F, Flies DB, Roche PC, Lu J, Zhu G, Tamada K, Lennon VA, Celis E, Chen L. Tumor-associated B7-H1 promotes T-cell apoptosis: a potential mechanism of immune evasion. Nature medicine 2002;8(8):793-800.
13. Wintterle S, Schreiner B, Mitsdoerffer M, Schneider D, Chen L, Meyermann R, Weller M, Wiendl H. Expression of the B7-related molecule B7-H1 by glioma cells: a potential mechanism of immune paralysis. Cancer research 2003;63(21):7462-7467.

14. Dong H, Chen L. B7-H1 pathway and its role in the evasion of tumor immunity. Journal of molecular medicine 2003;81(5):281-287.
15. Pardoll DM. The blockade of immune checkpoints in cancer immunotherapy. Nature reviews Cancer 2012;12(4):252-264.
16. Thompson RH, Gillett MD, Cheville JC, Lohse CM, Dong H, Webster WS, Krejci KG, Lobo JR, Sengupta S, Chen L, Zincke H, Blute ML, Strome SE, Leibovich BC, Kwon ED. Costimulatory B7-H1 in renal cell carcinoma patients: Indicator of tumor aggressiveness and potential therapeutic target. Proceedings of the National Academy of Sciences of the United States of America 2004;101(49):17174-17179.
17. Thompson RH, Gillett MD, Cheville JC, Lohse CM, Dong H, Webster WS, Chen L, Zincke H, Blute ML, Leibovich BC, Kwon ED. Costimulatory molecule B7-H1 in primary and metastatic clear cell renal cell carcinoma. Cancer 2005;104(10):2084-2091.
18. Thompson RH, Kuntz SM, Leibovich BC, Dong H, Lohse CM, Webster WS, Sengupta S, Frank I, Parker AS, Zincke H, Blute ML, Sebo TJ, Cheville JC, Kwon ED. Tumor B7-H1 is associated with poor prognosis in renal cell carcinoma patients with long-term follow-up. Cancer research 2006;66(7):3381-3385.
19. Ohigashi Y, Sho M, Yamada Y, Tsurui Y, Hamada K, Ikeda N, Mizuno T, Yoriki R, Kashizuka H, Yane K, Tsushima F, Otsuki N, Yagita H, Azuma M, Nakajima Y. Clinical significance of programmed death-1 ligand-1 and programmed death-1 ligand-2 expression in human esophageal cancer. Clinical cancer research : an official journal of the American Association for Cancer Research 2005;11(8):2947-2953.
20. Wu C, Zhu Y, Jiang J, Zhao J, Zhang XG, Xu N. Immunohistochemical localization of programmed death-1 ligand-1 (PD-L1) in gastric carcinoma and its clinical significance. Acta histochemica 2006;108(1):19-24.
21. Nomi T, Sho M, Akahori T, Hamada K, Kubo A, Kanehiro H, Nakamura S, Enomoto K, Yagita H, Azuma M, Nakajima Y. Clinical significance and therapeutic potential of the programmed death-1 ligand/programmed death-1 pathway in human pancreatic cancer. Clinical cancer research : an official journal of the American Association for Cancer Research 2007;13(7):2151-2157.
22. Zitvogel L, Tesniere A, Kroemer G. Cancer despite immunosurveillance: immunoselection and immunosubversion. Nature reviews Immunology 2006;6(10):715-727.
23. Taube JM, Anders RA, Young GD, Xu H, Sharma R, McMiller TL, Chen S, Klein AP, Pardoll DM, Topalian SL, Chen L. Colocalization of inflammatory response with B7-h1 expression in human melanocytic lesions supports an adaptive resistance mechanism of immune escape. Science translational medicine 2012;4(127):127ra137.
24. Iwai Y, Terawaki S, Honjo T. PD-1 blockade inhibits hematogenous spread of poorly immunogenic tumor cells by enhanced recruitment of effector T cells. International immunology 2005;17(2):133-144.
25. Hirano F, Kaneko K, Tamura H, Dong H, Wang S, Ichikawa M, Rietz C, Flies DB, Lau JS, Zhu G, Tamada K, Chen L. Blockade of B7-H1 and PD-1 by monoclonal antibodies potentiates cancer therapeutic immunity. Cancer research 2005;65(3):1089-1096.
26. Li B, VanRoey M, Wang C, Chen TH, Korman A, Jooss K. Anti-programmed death-1 synergizes with granulocyte macrophage colony-stimulating factor--secreting tumor cell immunotherapy providing therapeutic benefit to mice with established tumors. Clinical cancer research : an official journal of the American Association for Cancer Research 2009;15(5):1623-1634.
27. Curran MA, Montalvo W, Yagita H, Allison JP. PD-1 and CTLA-4 combination blockade expands infiltrating T cells and reduces regulatory T and myeloid cells within B16

melanoma tumors. Proceedings of the National Academy of Sciences of the United States of America 2010;107(9):4275-4280.
28. Brendler H CW, Scott WW. Prostatic Cancer: Further investigation of hormone relationships. Arch Surg 1949:433-440.
29. Prout GR, Jr., Brewer WR. Response of men with advanced prostatic carcinoma to exogenous administration of testosterone. Cancer 1967;20(11):1871-1878.
30. Donati RM, Ellis H, Gallagher NI. Testosterone potentiated 32P therapy in prostatic carcinoma. Cancer 1966;19(8):1088-1090.
31. Johnson DE, Haynie TP. Phosphorus-32 for intractable pain in carcinoma of prostate. Analysis of androgen priming, parathormone rebound, and combination therapy. Urology 1977;9(2):137-139.
32. Manni A, Bartholomew M, Caplan R, Boucher A, Santen R, Lipton A, Harvey H, Simmonds M, White-Hershey D, Gordon R, et al. Androgen priming and chemotherapy in advanced prostate cancer: evaluation of determinants of clinical outcome. Journal of clinical oncology : official journal of the American Society of Clinical Oncology 1988;6(9):1456-1466.
33. Szmulewitz R, Mohile S, Posadas E, Kunnavakkam R, Karrison T, Manchen E, Stadler WM. A randomized phase 1 study of testosterone replacement for patients with low-risk castration-resistant prostate cancer. European urology 2009;56(1):97-103.
34. Morris MJ, Huang D, Kelly WK, Slovin SF, Stephenson RD, Eicher C, Delacruz A, Curley T, Schwartz LH, Scher HI. Phase 1 trial of high-dose exogenous testosterone in patients with castration-resistant metastatic prostate cancer. European urology 2009;56(2):237-244.
35. Behre HM, Nieschlag E. Testosterone preparations for clinical use in males. In: Testosterone: Action,Deficiency. Cambridge University Press 2012;4th edition:309-335.
36. Schweizer MT, Antonarakis ES, Wang H, Ajiboye AS, Spitz A, Cao H, Luo J, Haffner MC, Yegnasubramanian S, Carducci MA, Eisenberger MA, Isaacs JT, Denmeade SR. Effect of bipolar androgen therapy for asymptomatic men with castration-resistant prostate cancer: results from a pilot clinical study. Science translational medicine 2015;7(269):269ra262.
37. Hussain MH, Pienta KJ, Redman BG, Cummings GD, Flaherty LE. Oral etoposide in the treatment of hormone-refractory prostate cancer. Cancer 1994;74(1):100-103.
38. Teply BA, Kachhap S, Eisenberger MA, Denmeade SR. Extreme Response to High-dose Testosterone in BRCA2- and ATM-mutated Prostate Cancer. European urology 2017;71(3):499.
39. Beer TM, Kwon ED, Drake CG, Fizazi K, Logothetis C, Gravis G, Ganju V, Polikoff J, Saad F, Humanski P, Piulats JM, Gonzalez Mella P, Ng SS, Jaeger D, Parnis FX, Franke FA, Puente J, Carvajal R, Sengelov L, McHenry MB, Varma A, van den Eertwegh AJ, Gerritsen W. Randomized, Double-Blind, Phase III Trial of Ipilimumab Versus Placebo in Asymptomatic or Minimally Symptomatic Patients With Metastatic Chemotherapy-Naive Castration-Resistant Prostate Cancer. Journal of clinical oncology : official journal of the American Society of Clinical Oncology 2017;35(1):40-47.
40. Kwon ED, Drake CG, Scher HI, Fizazi K, Bossi A, van den Eertwegh AJ, Krainer M, Houede N, Santos R, Mahammedi H, Ng S, Maio M, Franke FA, Sundar S, Agarwal N, Bergman AM, Ciuleanu TE, Korbenfeld E, Sengelov L, Hansen S, Logothetis C, Beer TM, McHenry MB, Gagnier P, Liu D, Gerritsen WR, Investigators CA. Ipilimumab versus placebo after radiotherapy in patients with metastatic castration-resistant prostate cancer that had progressed after docetaxel chemotherapy (CA184-043): a multicentre, randomised, double-blind, phase 3 trial. The lancet oncology 2014;15(7):700-712.

41. McCarthy EF. CT-guided needle biopsies of bone and soft tissue tumors: a pathologist's perspective. Skeletal radiology 2007;36(3):181-182.
42. Therasse P, Arbuck SG, Eisenhauer EA, Wanders J, Kaplan RS, Rubinstein L, Verweij J, Van Glabbeke M, van Oosterom AT, Christian MC, Gwyther SG. New guidelines to evaluate the response to treatment in solid tumors. European Organization for Research and Treatment of Cancer, National Cancer Institute of the United States, National Cancer Institute of Canada. Journal of the National Cancer Institute 2000;92(3):205-216.
43. Morris MJ, Scher HI, Stadler WM, Higano C, Basch E, Fizazi K, Antonarakis ES, Beer TM, Carducci MA, Chi KN, Corn PG, de Bono JS, Dreicer R, George DJ, Heath EI, Hussain M, Kelly WK, Liu G, Logothetis C, Nanus D, Stein MN, Rathkopf DE, Slovin SF, Ryan CJ, Sartor O, Small EJ, Smith MR, Sternberg CN, Taplin ME, Wilding G, Nelson PS, Schwartz LH, Halabi S, Kantoff PW, Armstrong AJ, Prostate Cancer Clinical Trials Working G. Trial Design and Objectives for Castration-Resistant Prostate Cancer: Updated Recommendations From the Prostate Cancer Clinical Trials Working Group 3. Journal of clinical oncology : official journal of the American Society of Clinical Oncology 2016;34(12):1402-1418.

APPENDIX A: MANAGEMENT ALGORITHM FOR IMMUNO-ONCOLOGY AGENTS

These general guidelines constitute guidance to the Investigator. The guidance applies to all immuno-oncology agents and regimens.

A general principle is that differential diagnoses should be diligently evaluated according to standard medical practice. Non-inflammatory etiologies should be considered and appropriately treated.

Corticosteroids are a primary therapy for immuno-oncology drug-related adverse events. The oral equivalent of the recommended IV doses may be considered for ambulatory patients with low-grade toxicity. The lower bioavailability of oral corticosteroids should be taken into account when switching to the equivalent dose of oral corticosteroids.

For subjects expected who require more than 4 weeks of corticosteroids or other immunosuppressants to manage an adverse event, consider the following recommendations:

- Antimicrobial/antifungal prophylaxis per institutional guidelines to prevent opportunistic infections such as Pneumocystis jiroveci (PJP) and fungal infections.

- Early consultation with an infectious disease specialist should be considered. Depending on the presentation, consultation with a pulmonologist for bronchoscopy or a gastroenterologist for endoscopy may also be appropriate.

- In patients who develop recurrent adverse events in the setting of ongoing or prior immunosuppressant use, an opportunistic infection should be considered in the differential diagnosis.

- Consultation with a medical or surgical specialist, especially prior to an invasive diagnostic or therapeutic procedure, is recommended.

The frequency and severity of the related adverse events covered by these algorithms will depend on the immuno-oncology agent or regimen being used.

GI Adverse Event Management Algorithm

Rule out non-inflammatory causes. If non-inflammatory cause is identified, treat accordingly and continue I-O therapy. Opiates/narcotics may mask symptoms of perforation. Infliximab should not be used in cases of perforation or sepsis.

Grade of Diarrhea/ Colitis (NCI CTCAE v4)	Management	Follow-up
**Grade 1**<br>Diarrhea: < 4 stools/day over baseline;<br>Colitis: asymptomatic	• Continue I-O therapy per protocol<br>• Symptomatic treatment	• Close monitoring for worsening symptoms.<br>• Educate patient to report worsening immediately<br>If worsens:<br>• Treat as Grade (G) 2 or 3/4
**Grade 2**<br>Diarrhea: 4-6 stools per day over baseline; IV fluids indicated <24 hours (hrs); not interfering with ADL<br>Colitis: abdominal pain; blood in stool	• Delay I-O therapy per protocol<br>• Symptomatic treatment	If improves to grade 1:<br>• Resume I-O therapy per protocol<br>If persists > 5-7 days or recur:<br>• 0.5-1.0 mg/kg/day methylprednisolone or oral equivalent<br>• When symptoms improve to grade 1, taper steroids over at least 1 month, consider prophylactic antibiotics for opportunistic infections, and resume I-O therapy per protocol.<br>If worsens or persists > 3-5 days with oral steroids:<br>• Treat as grade 3/4
**Grade 3-4**<br>Diarrhea (G3): ≥7 stools per day over baseline; incontinence; IV fluids ≥24 hrs; interfering with activities of daily living (ADL)<br>Colitis (G3): severe abdominal pain, medical intervention indicated, peritoneal signs<br>G4: life-threatening, perforation	• Discontinue I-O therapy per protocol<br>• 1.0 to 2.0 mg/kg/day methylprednisolone IV or IV equivalent<br>• Add prophylactic antibiotics for opportunistic infections<br>• Consider lower endoscopy	If improves:<br>• Continue steroids until grade 1, then taper over at least 1 month<br>If persists > 3-5 days, or recurs after improvement:<br>• Add infliximab 5 mg/kg (if no contraindication). Note: Infliximab should not be used in cases of perforation or sepsis

Patients on IV steroids may be switched to an equivalent dose of oral corticosteroids (e.g. prednisone) at start of tapering or earlier, once sustained clinical improvement is observed. Lower bioavailability of oral corticosteroids should be taken into account when switching to the equivalent dose of oral corticosteroids.

Renal Adverse Event Management Algorithm

Rule out non-inflammatory causes. If non-inflammatory cause, treat accordingly and continue I-O therapy

Grade of Creatinine Elevation (NCI CTCAE v4)	Management	Follow-up
**Grade 1**<br>Creatinine > upper limit of normal (ULN) and > than baseline but ≤ 1.5x baseline	• Continue I-O therapy per protocol<br>• Monitor creatinine weekly	<u>If returns to baseline</u>:<br>•Resume routine creatinine monitoring per protocol<br><u>If worsens:</u><br>•Treat as Grade 2 or 3/4
**Grade 2-3**<br>Creatinine > 1.5x baseline to ≤ 6x ULN	• Delay I-O therapy per protocol<br>• Monitor creatinine every 2-3 days<br>• 0.5 to 1.0 mg/kg/day methylprednisolone IV or oral equivalent<br>• Consider renal biopsy	<u>If returns to Grade 1:</u><br>•Taper steroids over at least 1 month, consider prophylactic antibiotics for opportunistic infections, and resume I-O therapy and routine creatinine monitoring per protocol<br><br><u>If elevations persist > 7 days or worsen:</u><br>•Treat as Grade 4
**Grade 4**<br>Creatinine > 6x ULN	• Discontinue I-O therapy per protocol<br>• Monitor creatinine daily<br>• 1.0-2.0 mg/kg/day methylprednisolone IV or IV equivalent<br>• Consult nephrologist<br>• Consider renal biopsy	<u>If returns to Grade 1</u>: Taper steroids over at least 1 month and add prophylactic antibiotics for opportunistic infections

Patients on IV steroids may be switched to an equivalent dose of oral corticosteroids (e.g. prednisone) at start of tapering or earlier, once sustained clinical improvement is observed. Lower bioavailability of oral corticosteroids should be taken into account when switching to the equivalent dose of oral corticosteroids.

Pulmonary Adverse Event Management Algorithm

Rule out non-inflammatory causes. If non-inflammatory cause, treat accordingly and continue I-O therapy. Evaluate with imaging and pulmonary consultation.

Grade of Pneumonitis (NCI CTCAE v4)	Management	Follow-up
**Grade 1**<br>Radiographic changes only	• Consider delay of I-O therapy<br>• Monitor for symptoms every 2-3 days<br>• Consider Pulmonary and Infectious Disease (ID) consults	• Re-image at least every 3 weeks<br><u>If worsens:</u><br>• Treat as Grade 2 or 3-4
**Grade 2**<br>Mild to moderate new symptoms	• Delay I-O therapy per protocol<br>• Pulmonary and ID consults<br>• Monitor symptoms daily, consider hospitalization<br>• 1.0 mg/kg/day methyl-prednisolone IV or oral equivalent<br>• Consider bronchoscopy, lung biopsy	• Re-image every 1-3 days<br><u>If improves:</u><br>• When symptoms return to near baseline, taper steroids over at least 1 month and then resume I-O therapy per protocol and consider prophylactic antibiotics<br><u>If not improving after 2 weeks or worsening:</u><br>• Treat as Grade 3-4
**Grade 3-4**<br>Severe new symptoms;<br>New/worsening hypoxia;<br>Life-threatening	• Discontinue I-O therapy per protocol<br>• Hospitalize<br>• Pulmonary and ID consults<br>• 2-4 mg/kg/day methylprednisolone IV or IV equivalent<br>• Add prophylactic antibiotics for opportunistic infections<br>• Consider bronchoscopy, lung biopsy	<u>If improves to baseline:</u><br>• Taper steroids over at least 6 weeks<br><u>If not improving after 48 hours or worsening:</u><br>• Add additional immunosuppression (e.g. infliximab, cyclophosphamide, intravenous immunoglobulin (IVIG), or mycophenolate mofetil)

Patients on IV steroids may be switched to an equivalent dose of oral corticosteroids (e.g. prednisone) at start of tapering or earlier, once sustained clinical improvement is observed. Lower bioavailability of oral corticosteroids should be taken into account when switching to the equivalent dose of oral corticosteroids.

Hepatic Adverse Event Management Algorithm

Rule out non-inflammatory causes. If non-inflammatory cause, treat accordingly and continue I-O therapy. Consider imaging for obstruction.

Grade of Liver Test Elevation (NCI CTCAE v4)	Management	Follow-up
**Grade 1**<br>AST or ALT > ULN to 3.0 x ULN and/or Total bilirubin (T. bili) > ULN - 1.5 x ULN	• Continue I-O therapy per protocol	• Continue liver function tests (LFT) monitoring per protocol<br>If worsens:<br>• Treat as Grade 2 or 3-4
**Grade 2**<br>AST or ALT > 3.0 to ≤ 5 x ULN and/or T. bili > 1.5 to ≤ 3 x ULN	• Delay I-O therapy per protocol<br>• Increase frequency of monitoring to every 3 days	If returns to baseline:<br>•Resume routine monitoring, resume I-O therapy per protocol<br><br>If elevations persist > 5-7 days or worsen :<br>• 0.5-1 mg/kg/day methylprednisolone or oral equivalent and when LFT returns to grade 1 or baseline, taper steroids over at least 1 month, consider prophylactic antibiotics for opportunistic infections, and resume I-O therapy per protocol
**Grade 3-4**<br>AST or ALT > 5 x ULN and /or T.bili >3 x ULN	• Discontinue I-O therapy*<br>• Increase frequency of monitoring to every 1-2 days<br>• 1.0 to 2.0 mg/kg/day methylprednisolone IV or IV equivalent**<br>• Add prophylactic antibiotics for opportunistic infections<br>• Consult gastroenterologist	If returns to grade 2:<br>• Taper steroids over at least 1 month<br><br>If does not improve in >3-5 days, worsens or rebounds:<br>• Add mycophenolate mofetil 1 gram (g) twice daily (BID)<br>• If no response within an additional 3-5 days, consider other immunosuppressants per local guidelines

Patients on IV steroids may be switched to an equivalent dose of oral corticosteroids (e.g. prednisone) at start of tapering or earlier, once sustained clinical improvement is observed. Lower bioavailability of oral corticosteroids should be taken into account when switching to the equivalent dose of oral corticosteroids.

***I-O therapy may be delayed rather than discontinued if AST/ALT ≤ 8 x ULN and T.bili ≤ 5 x ULN.**

**The recommended starting dose for grade 4 hepatitis is 2 mg/kg/day methylprednisolone IV.

Endocrinopathy Management Algorithm

Rule out non-inflammatory causes. If non-inflammatory cause, treat accordingly and continue I-O therapy. Consider visual field testing, endocrinology consultation, and imaging.

Asymptomatic thyroid stimulating hormone (TSH) elevation

→

- Continue I-O therapy per protocol
- If TSH < 0.5 x lower limit of normal (LLN), or TSH > 2 x ULN, or consistently out of range in 2 subsequent measurements: include free thyroxine (fT4) at subsequent cycles as clinically indicated; consider endocrinology consult

Symptomatic endocrinopathy

→

- Evaluate endocrine function
- Consider pituitary scan

Symptomatic with abnormal lab/pituitary scan:
- Delay I-O therapy per protocol
- 1-2 mg/kg/day methylprednisolone IV or by mouth (PO) equivalent
- Initiate appropriate hormone therapy

No abnormal lab/pituitary MRI scan but symptoms persist:
- Repeat labs in 1-3 weeks /MRI in 1 month

→

If improves (with or without hormone replacement):
- Taper steroids over at least 1 month and consider prophylactic antibiotics for opportunistic infections
- Resume I-O therapy per protocol
- Patients with adrenal insufficiency may need to continue steroids with mineralocorticoid component

Suspicion of adrenal crisis (e.g. severe dehydration, hypotension, shock out of proportion to current illness

→

- Delay or discontinue I-O therapy per protocol
- Rule out sepsis
- Stress dose of IV steroids with mineralocorticoid activity
- IV fluids
- Consult endocrinologist
- If adrenal crisis ruled out, then treat as above for symptomatic endocrinopathy

Patients on IV steroids may be switched to an equivalent dose of oral corticosteroids (e.g. prednisone) at start of tapering or earlier, once sustained clinical improvement is observed. Lower bioavailability of oral corticosteroids should be taken into account when switching to the equivalent dose of oral corticosteroids.

Skin Adverse Event Management Algorithm

Rule out non-inflammatory causes. If non-inflammatory cause, treat accordingly and continue I-O therapy.

Grade of Rash (NCI CTCAE v4)	Management	Follow-up
**Grade 1-2**<br>Covering ≤ 30% body surface area (BSA)*	• Symptomatic therapy (e.g. antihistamines, topical steroids)<br>• Continue I-O therapy per protocol	<u>If persists > 1-2 weeks or recurs:</u><br>• Consider skin biopsy<br>• Delay I-O therapy per protocol<br>• Consider 0.5-1.0 mg/kg/day methylprednisolone IV or oral equivalent. Once improving , taper steroids over at least 1 month, consider prophylactic antibiotics for opportunistic infections, and resume I-O therapy per protocol<br><u>If worsens:</u><br>• Treat as Grade 3-4
**Grade 3-4**<br>Covering >30% BSA; Life threatening consequences*	• Delay or discontinue I-O therapy per protocol<br>• Consider skin biopsy<br>• Dermatology consult<br>• 1.0-2.0 mg/kg/day IV methylprednisolone IV or IV equivalent	<u>If improves to Grade 1:</u><br>• Taper steroids over at least 1 month and add prophylactic antibiotics for opportunistic infections<br>• Resume I-O therapy per protocol

Patients on IV steroids may be switched to an equivalent dose of oral corticosteroids (e.g. prednisone) at start of tapering or earlier, once sustained clinical improvement is observed. Lower bioavailability of oral corticosteroids should be taken into account when switching to the equivalent dose of oral corticosteroids.
*Refer to NCI CTCAE v4 for term-specific grading criteria.

Neurological Adverse Event Management Algorithm

Rule out non-inflammatory causes. If non-inflammatory cause, treat accordingly and continue I-O therapy.

Grade of Neurological Toxicity (NCI CTCAE v4)	Management	Follow-up
**Grade 1**<br>Asymptomatic or mild symptoms; Intervention not indicated	• Continue I-O therapy per protocol	Continue to monitor the patient.<br><u>If worsens:</u><br>• Treat as Grade 2 or 3-4
**Grade 2**<br>Moderate symptoms; Limiting instrumental ADL	• Delay I-O therapy per protocol<br>• Treat symptoms per local guidelines<br>• Consider 0.5 to 1.0 mg/kg/day methylprednisolone IV or PO equivalent	<u>If improves to baseline</u>:<br>•Resume I-O therapy per protocol when improved to baseline<br><u>If worsens</u>:<br>• Treat as Grade 3-4
**Grade 3-4**<br>Severe symptoms; Limiting self-care ADL; Life-threatening	• Discontinue I-O therapy per protocol<br>• Obtain neurology consult<br>• Treat symptoms per local guidelines<br>• 1.0-2.0 mg/kg/day IV methylprednisolone IV or IV equivalent<br>• Add prophylactic antibiotics for opportunistic infections	<u>If improves to Grade 2</u>:<br>• Taper steroids over at least 1 month<br><br><u>If worsens or atypical presentation:</u><br>• Consider IVIG or other immunosuppressive therapies per local guidelines

Patients on IV steroids may be switched to an equivalent dose of oral corticosteroids (e.g. prednisone) at start of tapering or earlier, once sustained clinical improvement is observed. Lower bioavailability of oral corticosteroids should be taken into account when switching to the equivalent dose of oral corticosteroids.

APPENDIX B: PERFORMANCE STATUS CRITERIA

ECOG Performance Status Scale		Karnofsky Performance Scale	
**Grade**	**Description**	**%**	**Description**
0	Normal activity. Fully active, able to continue all predisease performance without restriction.	100	Normal, no complaints, no evidence of disease.
		90	Able to carry on normal activity, minor signs or symptoms of disease.
1	Symptoms, but ambulatory. Restricted in physically strenuous activity but ambulatory and able to carry out work of a light or sedentary nature (e.g. light housework, office work).	80	Normal activity with effort, some signs or symptoms of disease.
		70	Cares for self, unable to carry on normal activity or to do active work.
2	In bed < 50% of the time. Ambulatory and capable of all self-care but unable to carry out any work activities. Up and about more than 50% of waking hours.	60	Requires occasional assistance but is able to care for most needs.
		50	Requires considerable assistance and frequent medical care.
3	In bed > 50% of the time. Capable of only limited self-care, confined to bed or chair > 50% of waking hours.	40	Disabled, requires special care and assistance.
		30	Severely disabled, hospitalization indicated. Death not imminent.
4	100% bedridden. Completely disabled, cannot carry on any self-care, totally confined to bed or chair.	20	Very sick, hospitalization indicated. Death not imminent.
		10	Moribund, fatal processes progressing rapidly.
5	Dead.	0	Dead.

APPENDIX C: LABORATORY MANUAL

Pathology will be reviewed and processed centrally at Johns Hopkins. Biopsy specimens will be handled and sampled in a uniform fashion. The lead pathologist at Johns Hopkins University, Dr. Angelo De Marzo, will perform the central review and prepare tissue for correlative analysis.

***For Outside Sites ONLY*: Tumor tissue cores for FFPE will each be prepared into separate blocks (≥ 2 cores) and the cores for flash freezing will be combined into 1 block (≥ 2 cores). Specimens will be sent for prior review to the De Marzo Laboratory on dry ice to the following address:**

De Marzo Laboratory
Koch Cancer Research Building II
1550 Orleans Street, Room 138
Baltimore, Maryland 21231

Please email a copy of the Tissue Sample Information Form (Appendix G) and the shipment tracking number to the Lead Site coordinator on the day of shipment. Be sure to include a copy inside of the shipping container.

Tumor Tissue Specimens:

- 5 unstained slides and 1 H&E slide representative of tumor collected during the Screening period only, will be prepared and sent to Foundation Medicine for FoundationOne testing. Tumor will be placed in the FoundationOne specimen kit and shipped to the following address by the De Marzo Laboratory:

  Foundation Medicine, Inc.
  7010 Kit Creek Road
  Morrisville, NC 27560
  Phone: 888.988.3639

- 20 unstained slides representative of tumor will be prepared from all collected biopsies and sent to the De Marzo Laboratory for PD-1, PD-L1, CD4, CD8, Ki-67, gamma-H2AX, 53BP1, and RAD51 analysis.

- 5 unstained slides representative of tumor will be prepared from all collected biopsies and banked in the De Marzo Laboratory for optional tissue banking to be utilized for future research.

JHU Only: Page Tracy Jones (3-3186) for pick-up, processing, and transport of all tumor tissue to the De Marzo Laboratory.

MANAs/TAAs assay blood collection for JHU patients ONLY:

1. Blood will be drawn into ten 10mL purple top (EDTA) tubes for a total volume of 100mL for the MANAs/TAAs assay at C1D1, C4D1, and at progression (EOT).
2. Tubes will be sent to the Johns Hopkins Immune Processing Core Lab for PBMC processing & plasma isolation for immune studies.
3. Call the Immune Processing Core Lab (410-283-0693) for pick-up, processing, and transport.
4. Once processing is complete, the Immune Processing Core Lab will contact Eugene Shenderov, MD and/or Mark Markowski, MD within 24-48 hours for transfer to long-term storage.

Processing Instructions of MANAs/TAAs assay blood collection:

1. Uncap purple tops and pour blood into 50mL conicals.
2. Spin conicals at 2500rpm for 15min, 4°C, decel on 1.
3. Collect plasma layer and immediately freeze in 2mL cryovials at -80°C; 1.5mL per vial.
4. Dilute remaining blood 1 in 2 with PBS, gently mix up and down with a 10mL pipette.
5. Allow blood to sit at room temperature on a rocker for ~30min to bring up temperature.
6. Split diluted blood so there is ~35mL in each 50mL conical.
7. Continue processing according to Leucosep protocol.
8. Spin at 2200rpm for 20 minutes with accel and decel on "1" or off, depending on the centrifuge.
9. Collect the buffy coat layer into 50mL conicals, ensuring no more than 20mL of buffy coat layer goes into each conical.
10. Fill up remaining volume in the tubes with PBS.
11. Spin at 1400rpm for 10min.
12. Decant supernatant, break up pellet, and add 10mL PBS to each tube.
13. Spin at 1000rpm for 10min.
14. Assess the pellet. If there are a lot of RBCs, remove a few microliters and add 4mL ACK lysis buffer.
15. Fill 50mL conical with PBS and spin for 5min at 1400rpm.
16. Continue to decant off supernatant, break up pellet, and resuspend in 10mL PBS (30mL PBS for large blood draws or relevant volume depending on volume of blood received).
17. Count cells, spin at 1400rpm for 5min, continue to decant off supernatant.
18. Resuspend PBMC in pre-chilled AIM V media at a concentration of $10x10^6$ cells/mL.
19. Transfer 0.5mL aliquots ($5x10^6$ cells) to pre-labeled cryovials. Labels should contain patient lab ID, study number, time point, total number of cells, and date.
20. Quickly add 0.5mL 10% AIM V to each vial and cap. Gently rotate upside down twice.
21. Place in a slow freeze container at -80°C. Leave undisturbed overnight or for a minimum of 12 hours and a maximum of 24 hours.
22. Transfer into liquid nitrogen for long-term storage.

Chemokines Assay blood collection for JHU patients ONLY:

1. Blood will be drawn into one 10mL EDTA purple top tube at C1D1 predose, 3 hours post BAT injection on C1D1, 3 days after the first BAT injection (C1D4), C2D1 predose, and C7D1 predose.
2. Tubes should be sent to the Denmeade Laboratory for PBMC processing & plasma isolation for chemokine studies.
3. Call Marc Rosen (410-502-3825/443-275-8982/410-502-8810) or Mark Markowski, MD for pickup when collection is complete.
4. The Denmeade Laboratory will process the specimens and transport them to the Immune Processing Core Lab for analysis.

Processing instructions of Chemokines assay:

1. Uncap purple top tubes and transfer blood into 15mL conicals.
2. Spin conicals at 2500rpm for 15min, 4°C, decel on “1”.
3. Collect plasma layer and immediately freeze in 2mL cryovials at -80°C; 1mL per vial.

Rectal swab collection for JHU patients ONLY:

1. Rectal swabs will be collected via culture swab predose at C1D1, C4D1, and at C7D1.
2. Culture swabs will be sent to the Sfanos Laboratory for processing.
3. Call Sarah Ernst (443-386-8057) for pickup when collection is complete.

Collection instructions of Rectal swab cultures:

1. Write the subject ID, cycle visit, and the date and time of collection on the culture tubes for identification purposes.
2. Gently insert each swab about 1.2 inches (3cm) into anus and swirl the swab in a circular motion for 15-30 seconds.
3. Slowly remove each swab from anus without touching the skin and insert back into the tube.

APPENDIX D: GLOSSARY OF ABBREVIATIONS AND ACRONYMS

17-AAG	17-allylamino-17-demethoxygeldanamycin
17-DMAG	17-dimethylaminoethylamino-17-demethoxygeldanamycin
2-MPPA	2-(3-mercaptopropyl) Pentanedioic acid
AdEERS	Adverse Event Expedited Reporting System
ADR	Adverse drug reaction
ADT	Androgen-deprivation therapy
AE	Adverse event
AGA	Androgenetic alopecia
AI	Accumulation index
ALT	Alanine aminotransferase
AML	Acute myeloid leukemia
ANC	Absolute neutrophil count
ANOVA	Analysis of variance
APTT	Activated partial thromboplastin time
AR	Androgen receptor
ASAEL	Agent Specific Adverse Event List
AST	Aspartate aminotransferase
AUC	Area under the plasma concentration-time curve
AUC(INF)	Area under the concentration-time curve from time zero extrapolated to infinite time
AUC(0-T)	Area under the concentration-time curve from time zero to the time of the last quantifiable concentration
AUC(TAU)	Area under the concentration-time curve in one dosing interval
AUMC(INF)	Area under the moment concentration time curve extrapolated to infinity
A-V	Atrioventricular
β-HCG	Beta-human chorionic gonadotrophin
%BE	Percent biliary excretion
bid	bis in die (twice a day)
BLQ	Below limit of quantification
BMI	Body mass index
BP	Blood pressure
BSA	Body Surface Area
BUN	Blood urea nitrogen
C	Celsius
Ca++	Calcium
caBIG	Cancer Biomedical Informatics Grid
CAEPR	Comprehensive Adverse Event and Potential Risks
CALGB	Cancer and Leukemia Group B

CBC	Complete blood count
CCC	Clinical Consortium Committee
CCD	Central Consortium Database
CDE	Common data element
CDUS	Clinical Data Update System
CFR	Code of Federal Regulations
CI	Confidence interval
Cl-	Chloride
Clcr	Creatinine clearance
CLNR	Nonrenal clearance
CLR	Renal clearance
CLT	Total body clearance
CLT/F	Apparent total body clearance
Cm	Centimeter
Cmax	Maximum plasma concentration
Cmin	Trough observed concentration
COSTART	Coding Symbols for Thesaurus of Adverse Reaction Terms
CNS	Central nervous system
CR	Complete response
CRC	Clinical Research Center
CRDB	Clinical Research Database
CRF	Case report form
CRMIS	Clinical Research Management Information System
CRPC	Castration resistant prostate cancer
CT	Computerized tomography
CTC	Circulating tumor cell
CTCAE	Common Terminology Criteria for Adverse Events
CTEP	Cancer Therapy Evaluation Program
CTMS	Clinical Trials Monitoring Service
CTO	Clinical Trials Office
CV	Coefficient of variation
CYP	Cytochrome p-450
DCTD	Division of Cancer Treatment and Diagnosis
DEV	Deviation from the nominal value
%DEV	Percent deviation
dL	Deciliter
DHEA	Dehydroepiandrosterone
DHEA-S	Dehydroepiandrosterone sulfate
DHT	Dihydrotestosterone
DLT	Dose-limiting toxicity

DSM	Data and safety monitoring
EA	Extent of absorption
ECG	Electrocardiogram
ECOG	Eastern Cooperative Oncology Group
EDC	Electronic data capture
EEG	Electroencephalogram
EKG	Electrocardiogram
EORTC	European Organization for Research and Treatment of Cancer
ESF	Eligibility screening form
ESR	Expedited safety report
F	Bioavailability
FDA	Food and Drug Administration
FDG-PET	2-[18F]fluoro-2-deoxyglucose positron emitting tomography
FDHT	18-fluoro-dehydrotestosterone
%FE	percent fecal excretion
FISH	Fluorescence in situ hybridization
FSH	Follicle stimulating hormone
GAPDH	Glyseraldehayde-3-phophate dehydrogenase
GC	Gas chromatography
GCP	Good clinical practice
GCPII	Glutamate carboxypeptidase II enzyme
GFR	Glomerular filtration rate
GGT	Gamma-glutamyl transferase
GnRH	Gonadotropin-releasing hormone
HAT	Histone acetyltransferases
HCO3-	Bicarbonate
HDAC	Histone deacetylase
HIPAA	Health Insurance Portability and Accountability Act
HIV	Human immunodeficiency virus
HL7	American National Standards Institute's Health Level Seven
HPF	High power field
HPLC	High-performance liquid chromatography
HR	Heart rate
HRPC	Hormone-refractory prostate cancer
HRT	Hormone replacement therapy
HSP90	Heat-shock protein 90
ICD	International Classification of Diseases
ICH	International Conference on Harmonization
IEC	Independent Ethics Committee
IHC	Immunochemical

IM	Intramuscular
IMSL	International Mathematical Statistical Library
IND	Investigational new drug
INR	International normalized ratio
IP	Intraperitoneal
IRB	Institutional Review Board
ITT	Intent-to-treat population
IV	Intravenous
K	Slope of the terminal phase of the log concentration-time curve
K+	Potassium
K3EDTA	Potassium ethylenediaminetetraacetic acid
KLK1	Kallikrein 1
LBD	Ligand-binding domain
LC	Liquid chromatography
LCM	Laser capture microdissection
LC-MS	Liquid chromatography/mass spectrometry
LD	Longest diameter
LDH	Lactate dehydrogenase
LLQ	Lower limit of quantitation
ln	Natural logarithm
LOCF	Last observation carried forward
LOI	Letter of intent
LPF	Low power field
MAD	Maximum administered dose
MDS	Myelodysplasia
MedDRA	Medical Dictionary for Regulatory Activities
MIC	Minimum inhibitory concentration
MMP	Matrix metalloproteinase
MRI	Magnetic resonance imaging
MRT	Mean residence time
MRT(INF)	Mean residence time adjusted for infusion time
MRT(PO)	Mean residence time following oral administration
MRT(SS)	Mean residence time at steady-state
MSKCC	Memorial Sloan-Kettering Cancer Center
MS	Mass spectrometry
MTD	Maximum tolerated dose
N	Number of subjects or observations
NA	Not applicable
N/A	Not available
NBN	National Biospecimen Network

NCI	National Cancer Institute
NIH	National Institutes of Health
NOAEL	No observed adverse effect level
NOS	Not otherwise specified
NSAID	Nonsteroidal anti-inflammatory drug
NTX	N-telopeptide cross-link
NVB	Neurovascular bundle
OCR	Office of Clinical Research at MSKCC
PCCTC	Prostate Cancer Clinical Trials Consortium
PCRP	Department of Defense Prostate Cancer Research Program
PD	Progressive disease
PET	Positron emission tomography
PFS	Progression-free survival
PI	Principal investigator
PIN	Prostatic intraepithelial neoplasia
PK	Pharmacokinetics
PMB	Pharmaceutical Management Branch
PO	per os (by mouth)
PR	Partial response
PSA	Prostate-specific antigen
PSA-DT	Prostate-specific antigen doubling time
PSMA	Prostate specific membrane antigen
PT	Prothrombin time
PTT	Partial thromboplastin time
QC	Quality control
qd	quaque die (every day)
qRT-PCR	Quantitative reverse transcription-polymerase chain reaction
QOL	Quality of life
RBC	Red blood cell
RC	Research Council
RDBMS	Relational Database Management System
RDRC	Radioactive Drug Research Committee
RECIST	Response Evaluation Criteria in Solid Tumors
RP	Radical prostatectomy
RPC	eResearch Program Coordinator
RSA	Research Study Assistant
RSD	Relative standard deviation
%RSD	Percent relative standard deviation
SAE	Serious adverse event
SAHA	Suberoylanilide hydroxamic acid

SC	Subcutaneous
SD	Standard deviation
SD	Stable disease
Seq	Sequence
SHBG	Sex hormone binding globulin
SKI	Sloan-Kettering Institute for Cancer Research
SMD	Stable metabolic disease
SOP	Standard Operating Procedures
SPORE	Specialized Programs of Research Excellence
STAR	Symptom Tracking and Reporting
SUV	Standardized uptake value
t	Temperature
t1/2	Terminal half-life
T	Time
TAUC(TAU)	Trapezoidal area under the concentration-time curve in one dosing interval
TAUC(0-T)	Trapezoidal area under the concentration-time curve from time zero to the time of the last quantifiable concentration
TDP	Time to disease progression
TGP	Prostate-specific transglutaminase
tid	ter in die (3 times a day)
TMA	Tissue microarray
Tmax	Time of maximum observed concentration
TMPRSS2	Transmembrane protease, serine 2
TNM	Tissue, lymph node, metastases
TX	Treatment
ULN	Upper limit of normal
ULQ	Upper limit of quantitation
UR	Urinary recovery
VEGF	Vascular endothelial growth factor
Vss	Volume of distribution at steady-state
WBC	White blood cell
WHO	World Health Organization

APPENDIX E: ACCEPTABLE BIRTH CONTROL METHODS

Subjects with partners of childbearing potential, who are sexually active, must agree to the use of TWO highly effective forms of contraception (defined as a method that can achieve a failure rate of < 1% per year when used consistently and correctly) in combination [as listed below], throughout the period of taking study treatment and for at least 7 months after last dose of study drug(s), or they must totally/truly abstain from any form of sexual intercourse [see below].

Highly Effective Non-hormonal birth control methods include:

- Total sexual abstinence. Abstinence must continue for the total duration of study treatment and for at least 1 month after the last dose. Periodic abstinence (e.g. calendar ovulation, symptothermal post ovulation methods) and withdrawal are not acceptable methods of contraception.
- Vasectomised sexual partner PLUS male condom. With participant assurance that partner received post-vasectomy confirmation of azospermia.
- Tubal occlusion PLUS male condom.
- IUD PLUS male condom. Provided coils are copper-banded.

Highly Effective hormonal methods:

- Normal and low dose combined oral pills PLUS male condom.
- Cerazette (desogestrel) PLUS male condom. Cerazette is currently the only highly efficacious progesterone based pill.
- Hormonal shot or injection (e.g. Depo-Provera) PLUS male condom.
- Etonogestrel implants (e.g. Implanon, Norplant) PLUS male condom.
- Norelgestromin / EE transdermal system PLUS male condom.
- Intrauterine system [IUS] device (e.g. levonorgestrel releasing IUS - Mirena®) PLUS male condom.
- Intravaginal device (e.g. EE and etonogestrel) PLUS male condom.

APPENDIX F: ACTIONS REQUIRED IN CASES OF COMBINED INCREASE OF AMINOTRANSFERASE AND TOTAL BILIRUBIN – HY'S LAW

1. INTRODUCTION

During the course of the study the Investigator will remain vigilant for increases in liver biochemistry. The investigator is responsible for determining whether a patient meets potential Hy's Law (PHL) criteria at any point during the study. The Investigator participates, together with BMS clinical project representatives, in review and assessment of cases meeting PHL criteria to agree whether Hy's Law (HL) criteria are met. HL criteria are met if there is no alternative explanation for the elevations in liver biochemistry other than Drug Induced Liver Injury (DILI) caused by the Investigational Medicinal Product (IMP). The Investigator is responsible for recording data pertaining to PHL/HL cases and for reporting Adverse Events (AE) and Serious Adverse Events (SAE) according to the outcome of the review and assessment in line with standard safety reporting processes.

2. DEFINITIONS

<u>Potential Hy's Law (PHL):</u>

- Aspartate Aminotransferase (AST) or Alanine Aminotransferase (ALT) ≥ 3x Upper Limit of Normal (ULN) and Total Bilirubin (TBL) ≥ 2xULN at any point during the study irrespective of an increase in Alkaline Phosphatase (ALP).

<u>Hy's Law (HL):</u>

- AST or ALT ≥ 3xULN and TBL ≥ 2xULN, where no other reason, other than the IMP, can be found to explain the combination of increases, e.g. elevated ALP indicating cholestasis, viral hepatitis, another drug.

3. IDENTIFICATION OF POTENTIAL HY'S LAW CASES

In order to identify cases of PHL it is important to perform a comprehensive review of laboratory data for any patient who meets any of the following identification criteria in isolation or in combination:

- ALT ≥ 3xULN
- AST ≥ 3xULN
- TBL ≥ 2xULN

The Investigator will without delay review each new laboratory report and if the identification criteria are met will:

- Determine whether the patient meets PHL criteria (see Section 2 of this Appendix for definition) by reviewing laboratory reports from all previous visits.
- Promptly enter the laboratory data into the laboratory CRF.

4. FOLLOW-UP

Potential Hy's Law Criteria not met
If the patient does not meet PHL criteria the Investigator will:

- Perform follow-up on subsequent laboratory results according to the guidance provided in the Clinical Study Protocol.

Potential Hy's Law Criteria met
If the patient does meet PHL criteria the Investigator will:

- Notify the BMS representative who will then inform the central Study Team.
- The Study Physician contacts the Investigator, to provide guidance, discuss and agree an approach for the study patients' follow-up and the continuous review of data. Subsequent to this contact the Investigator will:
    - Monitor the patient until liver biochemistry parameters and appropriate clinical symptoms and signs return to normal or baseline levels, or as long as medically indicated.
    - Investigate the etiology of the event and perform diagnostic investigations as discussed with the Study Physician.
    - Complete the three Liver CRF Modules as information becomes available.
    - If at any time (in consultation with the Study Physician) the PHL case meets serious criteria, report it as an SAE using standard reporting procedures.

5. REVIEW AND ASSESSMENT OF POTENTIAL HY'S LAW CASES

The instructions in this Section should be followed for all cases where PHL criteria are met. No later than 3 weeks after the biochemistry abnormality was initially detected, the Study Physician contacts the Investigator in order to review available data and agree on whether there is an alternative explanation for meeting PHL criteria other than DILI caused by the IMP. BMS and the Principal Investigator will also be involved in this review together with other subject matter experts as appropriate. According to the outcome of the review and assessment, the Investigator will follow the instructions below:

If there is an agreed alternative explanation for the ALT or AST and TBL elevations, a determination of whether the alternative explanation is an AE will be made and subsequently whether the AE meets the criteria for a SAE:

- If the alternative explanation is not an AE, record the alternative explanation on the appropriate CRF.
- If the alternative explanation is an AE/SAE, record the AE/SAE in the CRF accordingly and follow BMS standard processes.

If it is agreed that there is no explanation that would explain the ALT or AST and total bilirubin level elevations other than the IMP:

- Report an SAE (report term 'Hy's Law') according to BMS standard processes.
    - The 'Medically Important' serious criterion should be used if no other serious criteria apply.
    - As there is no alternative explanation for the HL case, a causality assessment of "related" should be assigned.

If, there is an unavoidable delay of over 3 weeks in obtaining the information necessary to assess whether or not the case meets the criteria for HL, then it is assumed that there is no alternative explanation until such time as an informed decision can be made:

- Report an SAE (report term "Potential Hy's Law") applying serious criteria and causality assessment as per above.
- Continue follow-up and review according to agreed plan. Once the necessary supplementary information is obtained, repeat the review and assessment to determine whether HL criteria are met. Update the SAE report according to the outcome of the review.

6. ACTIONS REQUIRED FOR REPEAT EPISODES OF POTENTIAL HY'S LAW

This section is applicable when a patient meets PHL criteria on study treatment and has already met PHL criteria at a previous on study treatment visit.

The requirement to conduct follow-up, review and assessment of a repeat occurrence(s) of PHL is based on the nature of the alternative cause identified for the previous occurrence. The investigator should determine the cause for the previous occurrence of PHL criteria being met and answer the following question:

- Was the alternative cause for the previous occurrence of PHL criteria being met chronic or progressing malignant disease?

    - If No: follow the process described in Section 4 of this Appendix.
    - If Yes: Determine if there has been a significant change in the patient's condition# compared with when PHL criteria were previously met.
        - If there is no significant change no action is required.
        - If there is a significant change follow the process described in Section 4 of this Appendix.

A "significant" change in the patient's condition refers to a clinically relevant change in any of the individual liver biochemistry parameters (ALT, AST or total bilirubin) in isolation or in combination, or a clinically relevant change in associated symptoms. The determination of whether there has been a significant change will be at the discretion of the Investigator, this may be in consultation with the Study Physician if there is any uncertainty.

7. REFERENCES

FDA Guidance for Industry (issued July 2009) 'Drug-induced liver injury: Premarketing clinical evaluation':
http://www.fda.gov/downloads/Drugs/GuidanceComplianceRegulatoryInformation/Guidances/UCM174090.pdf

J1812 – COMbination of Bipolar Androgen Therapy and Nivolumab in Men with Metastatic Castration-Resistant Prostate Cancer [COMBAT-CRPC]

Johns Hopkins University Multi-center Phase II Clinical Trial

PI: Mark Markowski, MD, Ph.D

Appendix G: Tumor Sample Information Form

Subject No.: ________________________ Site No.: ____________________

Study Visit: ☐ Screening Period ☐ Cycle 4 Day 1 ☐ Progression/EOT

Date of Collection: __________________

FFPE Tissue:

No. of cores: ___________________

Consent obtained to allow storage of biospecimens for Future Research? ☐ Yes ☐ No

Flash Frozen Tissue:

No. of cores: ____________________ ☐ Not applicable for site

Label all cores and/or containers with the Fixative used (i.e. FFPE, Flash Frozen), Subject ID #, Cycle visit, & Date of collection.

Shipping Address:

De Marzo Laboratory
Koch Cancer Research Building II
1550 Orleans Street, Room 138
Baltimore, MD 21231

Please email a copy of this form and the shipment tracking number to the Lead Site coordinator on the day of shipment. Include a copy inside the shipping container.

All tumor specimens should be shipped on dry ice.
